# Combining Photochemical Oxyfunctionalization and Enzymatic Catalysis for the Synthesis of Chiral Pyrrolidines and Azepanes

**DOI:** 10.1021/acs.joc.4c02228

**Published:** 2025-01-08

**Authors:** Maria Logotheti, Susanne Gehres, Alexandre S. França, Uwe T. Bornscheuer, Rodrigo O. M. A. de Souza, Matthias Höhne

**Affiliations:** † Department of Biotechnology & Enzyme Catalysis, Institute of Biochemistry, University of Greifswald, Felix-Hausdorff-Str., 4, 17487 Greifswald, Germany; ‡ Biocatalysis and Organic Synthesis Group, Federal University of Rio de Janeiro, Chemistry Institute, 21941909 Rio de Janeiro, Brazil; § Institute of Chemistry, Technical University of Berlin, Straße des 17. Juni 115, 10623 Berlin, Germany

## Abstract

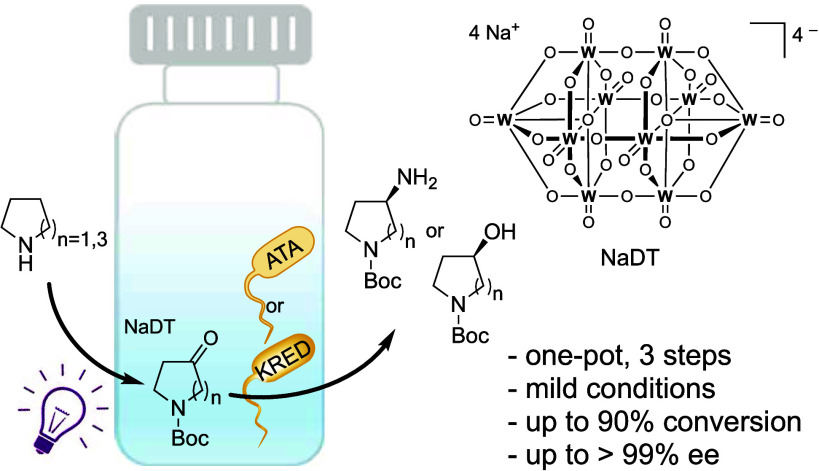

Chiral heterocyclic alcohols and amines are frequently used building blocks in the synthesis of fine chemicals and pharmaceuticals. Herein, we report a one-pot photoenzymatic synthesis route for *N*-Boc-3-amino/hydroxy-pyrrolidine and *N*-Boc-4-amino/hydroxy-azepane with up to 90% conversions and >99% enantiomeric excess. The transformation combines a photochemical oxyfunctionalization favored for distal C–H positions with a stereoselective enzymatic transamination or carbonyl reduction step. Our study demonstrates a mild and operationally simple asymmetric synthesis workflow from easily available starting materials.

## Introduction

**Figure 1:**
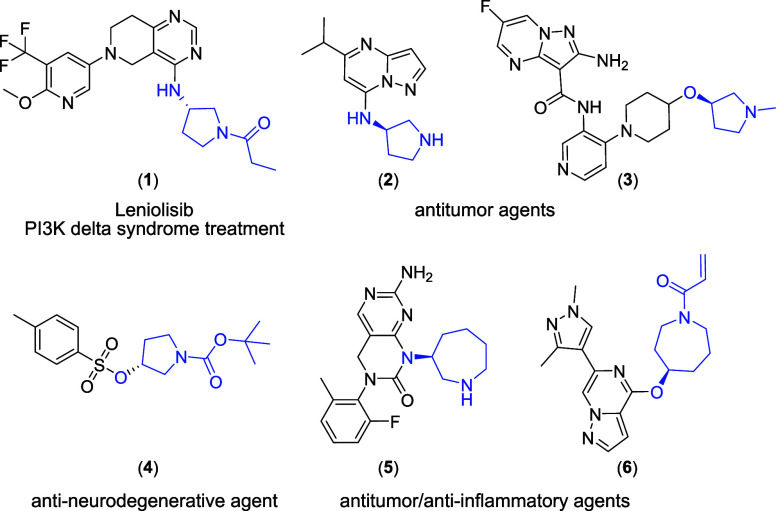
Examples of bioactive compounds containing a 3-amino/hydroxy-pyrrolidine or 4-amino/hydroxy-azepane moiety (highlighted in blue).

Chiral heterocyclic amines and alcohols are widespread building blocks needed in the synthesis of pharmaceuticals, natural products, and fine chemicals.^1,2^ Among them, 3-*N*-substituted pyrrolidines represent core motifs of many pharmacologically active ingredients, such as **1**–**4** (Figure 1). They are frequently investigated as scaffolds for inhibitor design in the development of antibacterials, antiproliferative substances, immunomodulators, and other types of therapeutic agents.^3^ For instance, Ceftobiprole^4^ and Leniolisib (**1**),^5^ as well as several clinical candidates, such as **2**, contain an enantiomeric 3-*N*-aminopyrrolidine moiety.^3^ Similarly, 3-*N*-hydroxypyrrolidine is a substructural motif of the drugs Darifenacin and Barnidipine^6^ as well as the bioactive compounds **3** and **4**.^7^ In recent years, azepane-based compounds gained momentum for a variety of pharmacological properties and represented an increasing number of promising clinical candidates.^8^ Enantiopure 4-*N*-aminoazepane- and 4-*N*-hydroxyazepane have been reported as synthetic intermediates for the preparation of several of these compounds, such as the kinase inhibitors (**5**) and (**6**)^9^ (Figure 1). Notably, *N*-Boc-protected enantiopure 3-amino/hydroxy-pyrrolidines and 4-amino/hydroxyazepanes are frequently used as intermediates in the synthesis of these compounds, as illustrated by molecules **1**–**6** (Figure 1), for which the *N*-Boc group is consistently used in earlier synthetic steps.^3,5,7,9^

Standard synthetic procedures for chiral amines and alcohols include mainly transition-metal-catalyzed asymmetric hydrogenation of imines and enamines, asymmetric transfer hydrogenation of ketones, or the kinetic resolution of racemates. The main limitations of these procedures are the high cost and potential for metal contamination, which are associated with the use of noble metals and large amounts of–sometimes harmful–organic solvents, as well as the often-compromised yields and optical purities, and long reaction times or procedural steps.^10−12^ Therefore, the development of environmentally friendlier synthetic procedures for the production of these chiral amine and alcohol synthons is highly desirable.^13^

**Figure 2:**
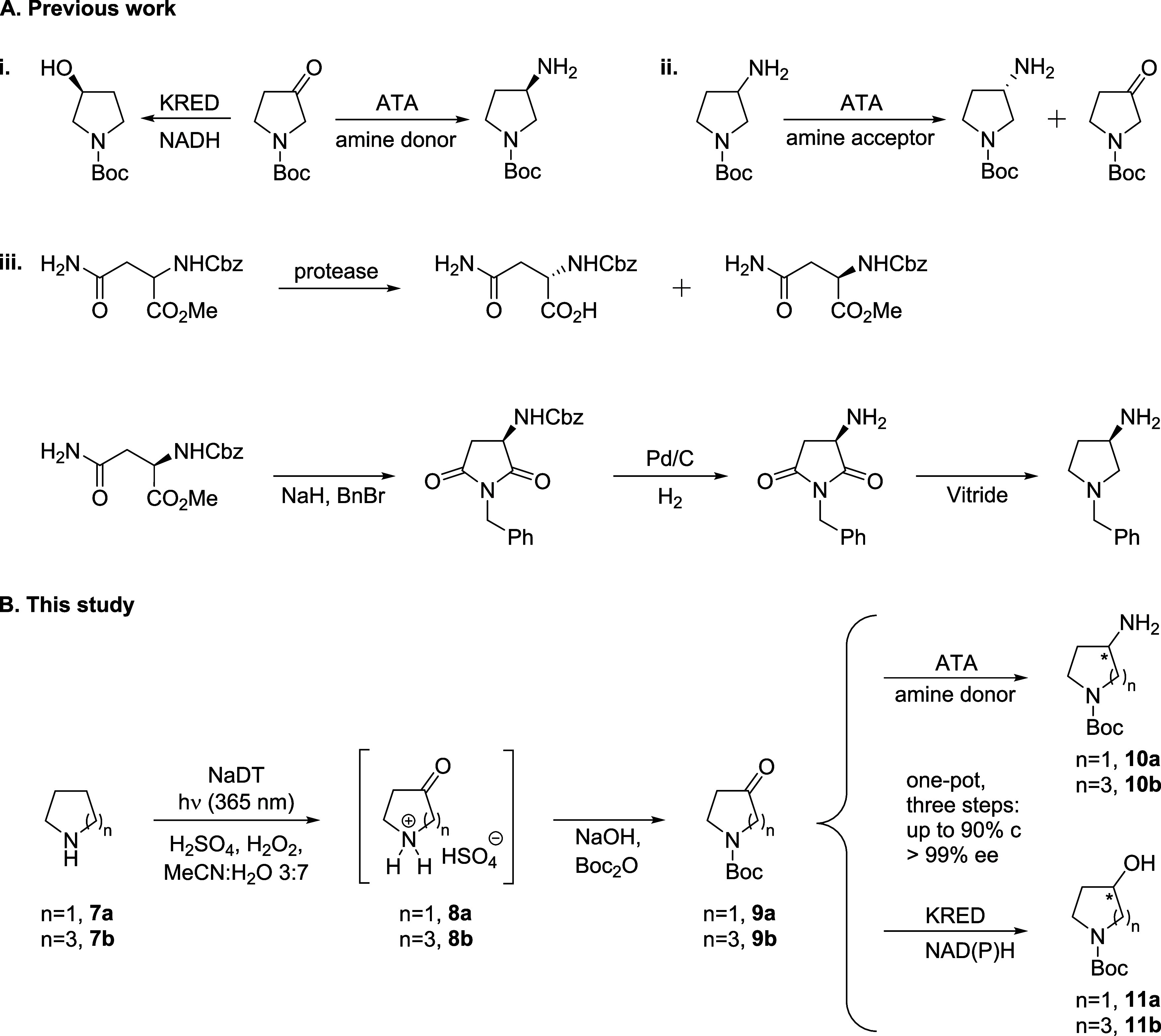
A. Examples of literature-reported biocatalytic syntheses of enantioenriched 3-aminopyrrolidines and 3-hydroxypyrrolidines. (i). Amine-transaminase (ATA)-catalyzed transamination or keto reductase (KRED)-catalyzed carbonyl reduction of *N*-protected-3-pyrrolidinone,^17^ (ii). ATA-mediated kinetic resolution of racemic *N*-Boc-3-aminopyrrolidine,^12^ (iii). Chemoenzymatic multipot preparation of chiral *N*-benzyl-3-aminopyrrolidine, with racemic resolution of *N*-protected-d,l-asparagine esters with proteases as the key step.^11^ B. Summarized reaction scheme of the one-pot photoenzymatic conversion of pyrrolidine or azepane into chiral *N*-Boc-protected 3-amino/hydroxypyrrolidines and 4-amino/hydroxyazepanes.

In this context, biocatalytic processes have been investigated with several reported applications in the synthesis of aliphatic heterocyclic amines and alcohols (Figure 2A).

In those cases, amine transaminases (ATAs)^14^ or keto reductases (KREDs)^15^ have been employed for the stereoselective transamination or carbonyl reduction of prochiral ketones, respectively.^16,17^ Kinetic resolution of racemates toward enantioenriched *N*-Boc-3-aminopyrrolidine, catalyzed by ATAs or hydrolases, has also been reported (Figure 2A).^11,12^ Constructing molecular complexity from readily available and inexpensive starting materials is a key goal in synthetic workflows designed to align with green chemistry principles.^18^ The application of successive selective catalytic transformations in one pot is a promising methodology as this eliminates intermediate isolation and purification procedures, which are often resource-intensive and time-consuming, contributing significantly to overall costs. This approach can significantly enhance cost and eco-efficiency, which are two key objectives when adapting a synthesis route for industrial applications.^19^

The design of synthesis routes that integrate chemocatalytic (metal-, photo-, organocatalytic) and enzymatic reaction steps is becoming more and more attractive as such an approach is harvesting the activation potential of chemocatalysis together with the high selectivity of enzymes.^20^ However, the frequent incompatibility between conditions of chemo- and biocatalysis remains a main bottleneck for merging these two worlds of catalysis.^21−25^ Several examples of chemoenzymatic syntheses have been reported in the literature, the majority of which refer to multipot procedures.^25^ The past decade has witnessed a significant increase in the number of reported one-pot chemoenzymatic synthesis examples, with a growing emphasis on both reaction and reactor engineering.^26^

Herein, we present a direct approach to accessing 3-amino- and hydroxypyrrolidines, starting from unfunctionalized pyrrolidine (**7a**). This method integrates a regioselective photooxyfunctionalization to generate 3-pyrrolidinone (**8a**), an *in situ N*-protection step to afford *N*-Boc-3-pyrrolidinone (**9a**), and a stereoselective biocatalytic transamination or carbonyl reduction in the same pot to provide the optically pure *N*-Boc-3-aminopyrrolidines (**10a**) or *N*-Boc-3-hydroxypyrrolidines (**11a**), respectively. We further applied this workflow to the chiral synthesis of 4-amino-and hydroxyazepanes (**10b** and **11b**), starting from unfunctionalized azepane (**7b**) (Figure 2B). In this way, we demonstrate a selective alternative route toward these building blocks. For this workflow, the photooxyfunctionalization step was based on the combined deactivation of proximal C–H bonds via amine protonation and decatungstate photocatalysis via H atom transfer (HAT), mediated through the decatungstate anion ([W_10_O_32_]^4–^).^27^ Schultz et al. reported regioselective oxyfunctionalizations of several aliphatic amines, at positions distal to the nitrogen atom with moderate to excellent selectivity and moderate yields, upon *N*-protection (Figure S2A).^28^

## Results and Discussion

We initiated this study by applying the workflow on the β-oxygenation of **7a** according to Schultz et al.^28^ employing a commercial photoreactor and a 365 nm LED lamp (Figures S2 and S3). We obtained good assay yields of **9a** (>**80**%), which were higher than the ones reported,^28^ and we attributed this to the different light setup and substrate concentrations used in this study (Table S9). To combine this transformation with the biocatalytic step as a one-pot sequence, the reaction conditions were rendered compatible. Though the reported conditions of the photo-oxygenation step offered a good starting point due to the use of a MeCN/water 1:1 mixture, the following workup involved a filtration step and the addition of Boc_2_O to the crude oxyfunctionalization product as a solution in DCM.^28^ This protection step was conducted due to the low stability of the 3-pyrrolidinone. However, most ATAs and KREDs are not stable at high concentrations of organic solvents or in biphasic systems.^14,15,29^ Based on this, as well as aiming for more sustainable solvents^30^ and a less laborious procedure, we went on modifying the solvent conditions of the oxygenation and protection step so that the biocatalytic transformation can follow in the same pot.

We initially studied the N-protection efficiency in different solvents. Among different protection strategies, Boc-protection was chosen due to the good solubility of Boc_2_O in water and the general good enzymatic acceptance of the Boc-substituent.^12^ For this, we mixed the more stable surrogate substrate 2-pyrrolidinone (**8c**) with 1.1 equiv of Boc_2_O in water/organic cosolvent mixtures, followed by incubation at RT. The reactions proceeded to completion within 2–3 h for several solvent compositions (Table S9).

The efficiency of the photo-oxygenation step was not significantly altered, while the MeCN content was decreased from 50 to 30%, with >80% conversions obtained for both low (100 mM) and high (400 mM) substrate concentrations. Reaction time increased and product yield dropped significantly at high substrate concentration and MeCN levels below 30%, whereas, at low substrate concentration, the MeCN level could be lowered below 20% (v/v) without a significant impact on the product yield or the reaction time. Alternative organic cosolvents were investigated; however, the highest yields were achieved in mixtures of MeCN/H_2_O (Table S9).

Based on these results, the final conditions of the photochemical transformation consisted of the substrate photo-oxygenation in 30% (v/v) MeCN, followed by basification with aqueous NaOH solution (0.7 equiv) and solvent-free addition of Boc_2_O (1.1 equiv). Under these conditions, the photo-oxygenation step was completed within 3 h for 0.1 mmol substrate, and within 5-6 h for 1.4 mmol substrate, at 400 mM concentration. Boc-protection was completed within 2 h, at low or high substrate concentration (Figure S2B).

The candidate enzymes were selected based on reported information or previous in-house knowledge on their substrate scope and consisted of both wild-type enzymes and engineered variants (Table S1). ATA activity toward **9a** was studied via activity assays and biocatalytic setups, using crude *Escherichia coli* cell extract. Biocatalytic conversion of **9a** was studied in the presence of isopropylamine (IPA) or d/l-alanine/GDH as amino donors. The highest activities were determined for *Rpo*TA(3HMU), *Cvi*TA, *Cvi*TA_M1, and *CD5*TA_M1 (all (*S*)-selective) and for ATA-117-Rd11 ((*R*)-selective) (Tables S10 and S11). These ATAs converted in the first setups >70% of **9a** into the respective chiral amine within 20 h with >99% ee and ≥98% ee for 3HMU and ATA-117-Rd11, respectively (Table S11). Furthermore, these biocatalysts could also be used in the form of resting or lyophilized cells with similar performance (Table S12). For cost and practicality reasons, we avoided using the alanine/GDH system for the biocatalytic transamination in the final setup, and instead we chose an excess of the amino donor IPA. We then determined the optimal composition for the biocatalytic reactions via separately varying cofactor and amino donor concentrations, cosolvent percentage, pH value and type of buffer. KRED activity towards **9a** was screened using resting or lyophilized cells (Table S13). Among them, *Fs*KRED and *Ls*KRED gave the highest conversions (>70% of **9a** in first setups) with >99% ee and ≥98% ee, using an excess of isopropanol (*i*-PrOH). From different NAD(P)H recycling options, we chose an excess of *i*-PrOH as a hydride donor for the final setup. We went on to determine the composition of the biocatalytic reactions via separately varying cofactor concentration, *i*-PrOH/DMSO levels, and type of buffer.

Next, we investigated whether components of the first reaction steps negatively impacted the biocatalysis outcome, and we examined the potential effect of MeCN and chemical components of the first two reaction steps on the transaminase activity (Tables 1, S14 and S15).

**Table 1:** Compatibility of ATA and KRED toward Components from the Photocatalytic Production of **9a**

entry	variation from standard condition^c^	conversion (%)			
		3HMU	ATA-117-Rd11	*Fs*KRED	*Ls*KRED
1	none^a^	72 ± 2	81 ± 10	83 ± 1	79 ± 2
2	7% DMSO	81 ± 10	n/a	73 ± 5	n/a
3	+Boc_2_O (10 mM)^b^	78 ± 1	n/a	70 ± 5	n/a
4	+Boc_2_O (100 mM)^b^	49 ± 5	n/a	42 ± 2	n/a
5	+NaDT (1 mM), Boc_2_O (10 mM)^b^	71 ± 1	n/a	75 ± 2	n/a
6	2.5% MeCN	62 ± 2	95 ± 4	n/a	n/a
7	5% MeCN	70 ± 1	n/a	83 ± 3	76 ± 1
8	7% MeCN	72 ± 4	93 ± 1	13 ± 2	80 ± 2
9	10% MeCN	49 ± 2	96 ± 0.4	n/a	72 ± 2

a Standard conditions of the transamination: 0.1 mg/mL crude cell extract containing overexpressed 3HMU or ATA-117-Rd11, 1 M IPA, 0.1 mM PLP, 20 mM **9a** and 2% (v/v) DMSO in 50 mM HEPES (=pH 8); standard conditions of the ketoreduction: 50% (v/v) resting cells of *Fs*KRED or *Ls*KRED overexpressed cell culture (final OD_600_: 10), 5% (v/v) glucose, 20 mM **9a** and 0.4% (v/v) DMSO, in 50 mM NaPi (= pH 7 (*Ls*KRED) or 8 (*Fs*KRED)).

b Entries 3–5 refer to setups containing 7% (v/v) DMSO in addition to the tested reagent.

c In all shown cases, 0.5 mL-reactions were carried out at 30 °C. Reactions were extracted after 20 h, followed by GC-MS analysis. No effect on ee values from a variation of these conditions was observed.

3HMU tolerated up to 7% (v/v) MeCN, ATA-117-Rd11 and *Ls*KRED even 10% (v/v), whereas *Fs*KRED lost activity at concentrations >5% (v/v) MeCN. To decipher, whether NaDT and Boc_2_O have a negative effect on the biocatalysis step, these reagents were added at concentrations within and above the theoretical residual level in crude product **9a**, but no notable impact on the biocatalytic reactions was concluded. Based on the above results, we decided to adjust the MeCN level of crude **9a** to 4–5% (v/v) for the biocatalytic transformation to take place in the same pot via a 6–7.6-fold increase of the total reaction volume. The photoenzymatic reaction was performed with 400 mM **7a**, which was converted to **8a** within 3–6 h, while irradiated at 365 nm (Figure S2B). Afterward, the vial was removed from the light source, and following a basification step with 10 M NaOH to pH 9, it was charged with neat Boc_2_O (1.1 equiv) and incubated for 2 h at RT under stirring. As a next step, the crude chemical reaction product was diluted with buffer, and the reaction cocktail for the biocatalytic transamination or reduction, which contained the respective ATA- or KRED-containing lyophilized cells, thereby reducing MeCN levels to 4–5% (v/v) (see Sections 6.1 and 6.2, and Figure S3 in the Supporting Information). After a 20 h incubation at 30 °C, >80% conversion and up to >99% ee were determined for **10a** and **11a**. The products were isolated with up to 45% overall yields (Table 2). After demonstrating the feasibility of this one-pot approach with the model substrate pyrrolidine, we applied the workflow to substrate azepane (**7b**) to explore its potential for scope expansion. This way, we were able to obtain the products **10b** and **11b** with up to 80% conversions and up to >99% ee. In this case, the conversion of **7b** to **9b** was >90%. The ATAs 3HMU (*R*-selective) and ATA-117 (*S*-selective) and the KREDs *Ls*KRED (*S*-selective) and Codexis KRED-NADH-101 (*R*-selective) performed best among the investigated biocatalysts (Tables 2, S11, S13, and S16). All biocatalysts were applied as lyophilized cells (except for KRED-NADH-101, which was provided as a lyophilized enzyme). Interestingly, inversion of each biocatalyst’s standard enantiopreference was observed for substrate **9b**, which was determined via the use of authentic enantiomerically pure standards. As the transamination of **9b** is reported here for the first time, and the switch was observed with both (*R*)- and (*S*)-selective ATAs, similar to the behavior of the two KREDs, we speculate that the differences in the adopted conformations of the seven- and five-membered ketones **9a** and **9b** lead to a different positioning of these ketones in the active sites, thus resulting in the switch of the enantiopreference.

**Table 2:**
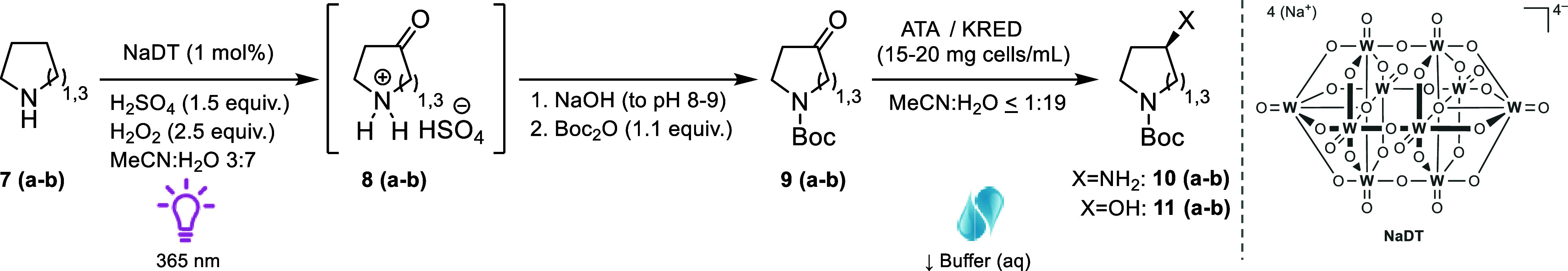

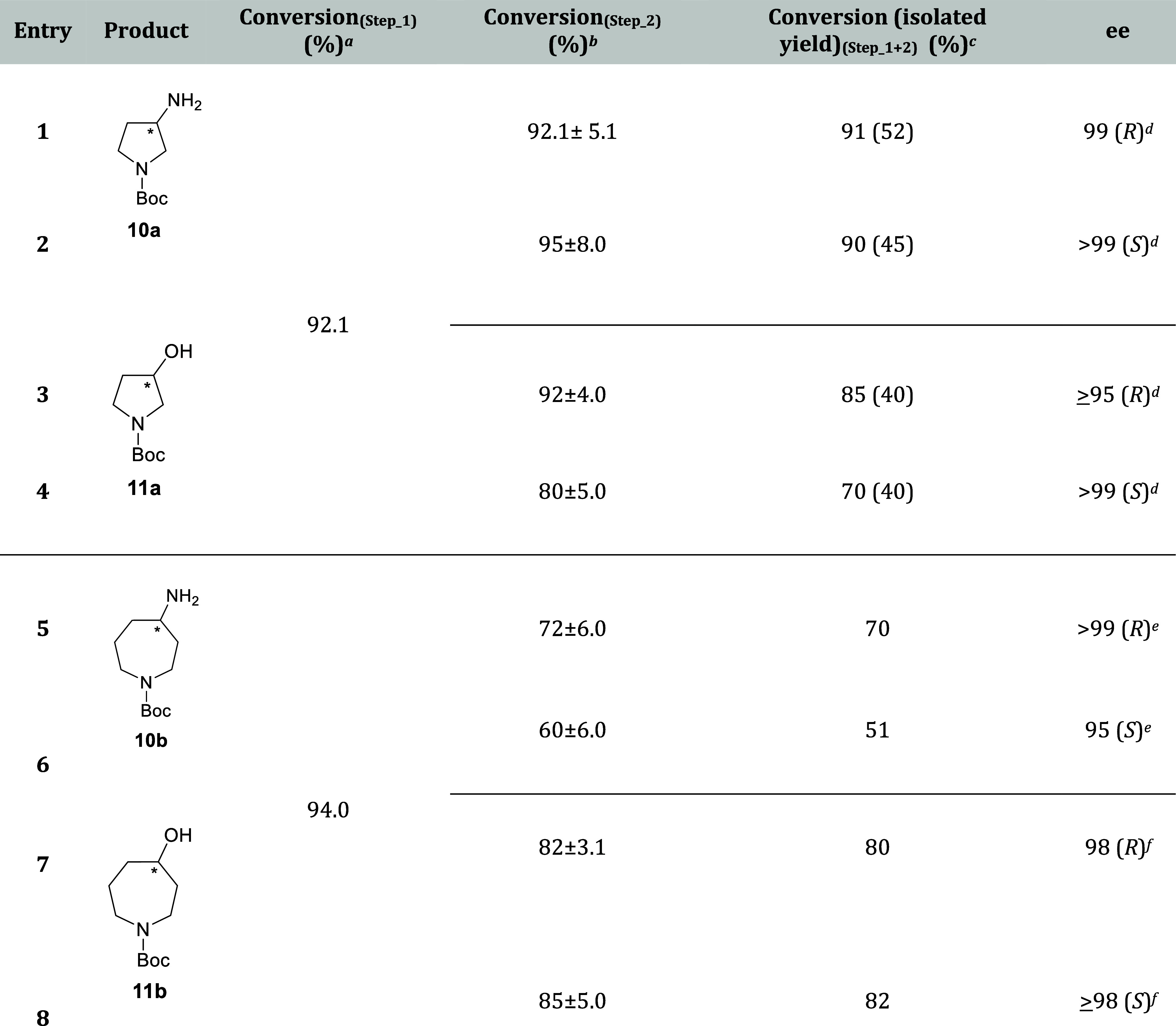
Summary of Photochemical and Biocatalytic Conversions, Isolated Yields, and ee Values^g^

a **7a**/**7b** % conversions **into 9a**/**9b** upon photooxyfunctionalization and following *N*-protection (step 1), as determined via GC-MS analysis.

b **9a**/**9b** biocatalytic % conversions into the final amine or alocohol products (step 2), as determined via GC-MS analysis.

c **7a**/**7b** total % conversions into the final amine or alcohol products, as determined via GC-MS analysis; isolated yields are shown in parentheses. % ee was determined via

d chiral GC-MS analysis,

e chiral HPLC analysis, or

f chiral GC-FID analysis. % Conversions of step 1 were reproducible in three independent experiments and showed a standard deviation <10%. % Conversions of step 2 are shown with standard deviations calculated based on triplicate measurements. % Conversions of step 3 are the average of 3 independent setups and showed a standard deviation <10%.

g Reaction conditions refer to 1.42 mmol (400 mM) starting pyrrolidine/azepane substrate (**7a**/**7b**). The crude *N*-Boc-3-pyrrolidinone/ *N*-Boc-hexahydro-1*H*-azepin-4-one (**9a**/**9b**) intermediate obtained in the first step was diluted 6-fold with 50 mM HEPES, pH = 8 , and the reaction cocktail for biocatalytic transamination. For the biocatalytic keto reduction, the **9a**/**9b** intermediate was diluted with 50 mM potassium phosphate buffer, pH = 7 or 8, and the reaction cocktail for biocatalytic ketoreduction. The final reaction medium contained in addition: 1 mM PLP and 1 M IPA, or 0.5 mM NAD(P)^+^ and 10% *i*-PrOH, for ATA- or KRED-biocatalysis, respectively. When the Codexis enzyme KRED-NADH-101 was applied, the conditions were partly modified (see section 6.2 in the Supporting Information).

Together, the above results indicate that the photoenzymatic synthesis workflow developed here provides direct access to the above enantiopure azacyclic alcohols and amines, which serve as frequent synthetic intermediates toward the synthesis of pharmaceuticals. The presented approach allows high to good conversions of the initial starting material into the final chiral product in a telescopic mode, whereas it makes use of inexpensive starting materials and components (catalysts, additives). At the same time, use of hazardous solvents and toxic metals is avoided, compared to other reported procedures.^10^ This method is stereoselective, as well as regioselective, toward the synthesis of enantiopure **10a** and **11a** and the–less studied in biocatalysis–**10b** and **11b**. The given photoenzymatic synthetic workflow was not applied to the six-membered-ring substrate piperidine. Although it was converted with similar efficiency, the photooxidation yielded a mixture of the 4- and 3-keto products in a 2:1 ratio.^28^ To the best of our knowledge, this workflow represents the first one-pot chemobiocatalytic asymmetric synthesis method for the aforementioned building blocks. The substrate scope of this approach could be further expanded in the future, as the reported substrate scope of the photooxyfunctionalization reaction^28^ and preliminary chemical (Table S17) and biocatalytic experiments of our study indicate. Moreover, we expect that catalysts’ immobilization and application of this concept under flow conditions, will further increase its efficiency.^28,31^

## Data Availability

The data underlying this study are available in the published article and its Supporting Information.

## Supporting Information Available

Contains the mentioned experimental section and additional experimental and analytical data, including NMR spectra (PDF)

## Abbreviations

ATA: amine transaminase
DCM: dichloromethane
DMSO: dimethyl sulfoxide
GDH: glucose dehydrogenase
KRED: keto reductase
MeCN: acetonitrile
NAD(P)H: β-nicotinamide ade nine dinucleotide (phosphate), reduced disodium salt hydrate
PLP: pyridoxal-5′-phosphate
NaDT: sodium decatungstate
