# Supplementary material for: Combining Photochemical Oxyfunctionalization and Enzymatic Catalysis for the Synthesis of Chiral Pyrrolidines and Azepanes
Source: J Org Chem. 2025 Jan 8;90(2):1036–43. doi: 10.1021/acs.joc.4c02228 (PMC11744798; doi:10.1021/acs.joc.4c02228)
Supplement: Supplementary file 1 — jo4c02228_si_001.pdf [file jo4c02228_si_001.pdf]

Supporting Information

Combining Photochemical Oxyfunctionalization and Enzymatic Catalysis for the Synthesis of Chiral Pyrrolidines and Azepanes

Maria Logotheti[1], Susanne Gehres[1], Alexandre Franca[2], Uwe T. Bornscheuer[1], Rodrigo O. M. A. de Souza[2*], Matthias Höhne[3*]

[1]Department of Biotechnology & Enzyme Catalysis, Institute of Biochemistry, University of Greifswald, Felix-Hausdorff-Str. 4, 17487 Greifswald, Germany

[2]Biocatalysis and Organic Synthesis Group, Federal University of Rio de Janeiro, Chemistry Institute, 21941909 Rio de Janeiro, Brazil

[3]Institute of Chemistry, Technical University of Berlin, Straße des 17. Juni 115, 10623 Berlin, Germany

Table of contents

1. General experimental information and materials 3

2. Gene expression and preparation of biocatalysts 4

3. Activity assays 10

4. Chemical synthesis workflows 11

5. Biocatalytic synthesis workflows..13

6. One-pot regio-and stereoselective synthesis of amines and alcohols from pyrrolidine or azepane..14

7. Analytical methods..16

8. Product characterization...20

9. Study of reaction conditions for aliphatic amine photo-oxyfunctionalization and *N*-protection, before coupling to biocatalysis...23

10. Biocatalyst activity screening...24

11. Photoenzymatic setup evaluation...27

12. Stereochemistry assignment via GC- and HPLC analysis...28

13. Standard compound GC-spectra...37

14. MS-spectra...46

15. NMR-spectra..47

16. References...53

1. General experimental information and materials

All chemicals were used without further purification. Hydrogen peroxide, 30% solution (w/v) was purchased from Fisher Scientific GmbH (Schwerte, Germany), β-nicotine amide adenine dinucleotide phosphate disodium salt (NADP) and β-nicotine amide adenine dinucleotide (NAD), as well as their reduced forms, were purchased from Carl Roth GmbH & Co. KG (Karlsruhe, Germany), acetophenone from Fluka™ Honeywell International Inc. (Charlotte, NC, USA), phenylmethylsulfonyl fluoride (PMSF) from abcr GmbH (Karlsruhe, Germany) and HEPES from Carl Roth GmbH & Co. KG (Karlsruhe, Germany). (*S*)-*N*-Boc-4-aminoazepane and (*R*)-*N*-Boc-4-hydroxyazepane of 95% purity were purchased as standards from abcr GmbH (Karlsruhe, Germany), *N*-Boc- 2-pyrrolidinone of 95% purity was purchased as standard from Merck KGaA (Darmstadt, Germany), organic solvents were of HPLC grade and derived from Riedel-de-Haën™ Honeywell International Inc. (Charlotte, NC, USA). *E. coli* DH5α cells and *E. coli* BL21 (DE3) strains were purchased from GenScript Biotech Co., Ltd (Nanjing, China). Cell growth media were purchased from Merck KGaA (Darmstadt, Germany). Commercial enzymes were purchased from Merck KGaA (Darmstadt, Germany) or Thermo Fisher Scientific (Waltham, MA, USA). The rest of chemicals and supplies were purchased from Merck KGaA (Darmstadt, Germany), and were of > 98 purity. The CODEX® KRED SCREENING KIT (Catalog No. PRO-006-04) was used according to the manufacturer's instructions (Codexis, Inc., Redwood City, US)

ALUGRAM® SIL G/UV254 TLC plates were used and purchased from Macherey-Nagel (Düren, Germany), Silica gel 60 was purchased from Merck KGaA (Darmstadt, Germany).

Gas-chromatography (GC) analytics were performed on a Nexis GC-2030 with flame ionization detection (Shimadzu Deutschland GmbH, Germany, Duisburg). GC-mass spectrometry (MS) was conducted on a GC-2010 Plus equipped with a QP2010 SE detector (Shimadzu Deutschland GmbH, Germany, Duisburg). High performance liquid chromatography (HPLC) analytics were performed on a VWR® -Hitachi LaChrom ELITE® HPLC with an L-2400 UV detector.

NMR-spectroscopy was performed using an Avance II 300 (Bruker Daltonics GmbH & Co. KG, Germany, Bremen) or a JOCFT-NMR Spectrometer Avance III 400 MHz (Bruker BioSpin GmbH, Rheinstet, Germany). Chemical shifts are given in parts per million (ppm) and were calibrated with deuterated solvents: $CDCl_3$ (^{1}H 7.26 ppm, ^{13}C 77.16 ±0.06 ppm), DMSO-d_6 (^{1}H 2.05 ppm, ^{13}C 39.52 ±0.06 ppm) or with tetramethylsilane (TMS) (^{1}H 0.0 ppm ,^{13}C 0.0 ppm) as internal standard.[1] Proton multiplicities are abbreviated as following: s (singlet), d (doublet), dd (doublet of a doublet), ddd (doublet of a doublet of a doublet), t (triplet), dt (doublet of a triplet), ddt (doublet of a doublet of a triplet), q (quartet), dq (doublet of a quartet), p (quintet), h (hextet), m (multiplet). Coupling constants (J) are shown in Hz (Hertz).

All catalytic reactions, including all light-promoted reactions were performed in clear glass (borosilicate) vials (screw top, clear glass, 1.5 mL or Wheaton sample vials, clear, 8 or 22 mL) with pressure release caps or screw caps, from Merck KGaA (Darmstadt, Germany). Magnetic stirring was performed with MR 3001 K, Heidolph (Schwabach, Germany). For photochemical reactions, the commercial light device EvoluChem PhotoRedOx Box™ and a 365 nm LED lamp (18 W, EvoluChem 365PF), from HepatoChem Inc. (Beverly, MA, USA) were employed. Spectrophotometric assays were performed using an Infinite® 200 PRO (TECAN) plate reader and 96-well UV-transparent (UV-Star, Greiner Bio-One GmbH)- or standard polystyrene (PS, clear, Greiner Bio-One GmbH) microplates. Cell lyophilization was performed with a Christ Freeze Dryer Alpha 1-2 (230 V. 50 Hz. 700 W) and a rotary vane pump (RZ 2. 2,2 m³/h. 0,0004 mbar, 230 V. 50 Hz), purchased from Martin Christ Freeze Dryers GmbH (Osterode am Harz, Germany) and Vacuubrand GmbH + Co KG (Wertheim, Germany), respectively.

Substrates

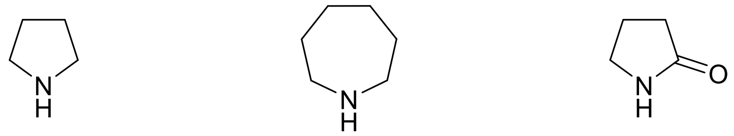

7a **7b** **7c**

Intermediates

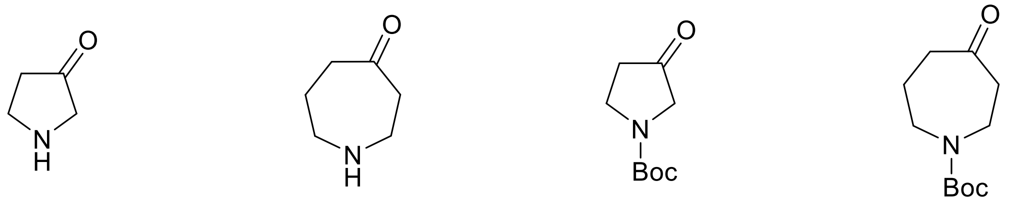

8a **8b** **9a** **9b**

Products

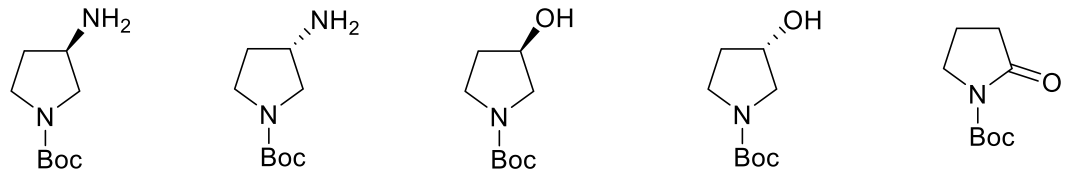

(*R*)-**10a** (*S*)-**10a** (*R*)-**11a** (*S*)-**11a** **9c**

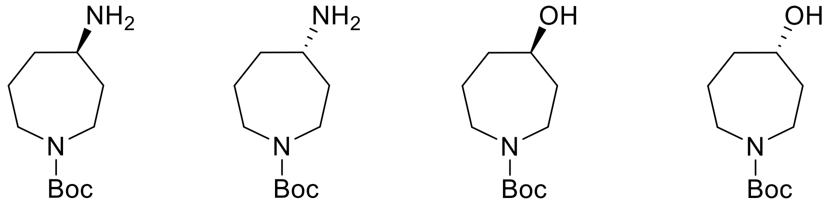

(*R*)-**10b** (*S*)-**10b** (*R*)-**11b** (*S*)-**11b**

Figure S1. List of substrates, intermediates, and products mentioned in this study.

2. Gene expression and preparation of biocatalysts

2.1 Bacterial strains and gene constructs

The following *E. coli* strains were used for cloning and expression, respectively*: E. coli* TOP10 [*F- mcr*A Δ*(mrr-hsd*RMS-*mcr*BC*) φ80lac*ZΔM15 Δ*lac*X74 *rec*A1 *ara*D139 Δ*(araleu)*7697 *gal*U *gal*K *rps*L *(Str*R*) end*A1 *nup*G] and *E. coli* BL21 (DE3) [*fhu*A2 *lon omp*T *gal (λ* DE3*) dcm* Δ*hsd*S *λ* DE3 = *λ sBam*HI*o* Δ*Eco*RI-B *int::(lac*I*::Plac*UV5*::T7 gene*1*)* i21 Δ*nin*5]

Table S1. List of biocatalysts and respective gene constructs examined in this study

Entry	TA	Source	Vector	Accession	Reference
1	3HMU	*Ruegeria pomeroyi*	pET-28b[2]	3HMU_A	6
2	ATA-117	*Arthrobacter* sp. KNK168	pET-28b	3WWH_A	7-9
3	AfTA	*Aspergillus fumingatus*	pET-28b	4CHI	10-11
4	AtTA	*Aspergillus terreus*	pET-28b	4CE5	12-13
5	VflTA	*Vibrio fluvialis*	pET-24b[3]	4E3Q	14-15
6	CviTA	*Chromobacterium vio-laceum*	pET-24b	4A6T	16-17
7	TA-01	*Bilophilia wadsworthia*	pET-28b[4]	MT828894	18
8	TA-05	*Halomonas elongata*	pET-28b	MT828898	
9	TA-10	*Burkholderia multivorans*	pET-28b	MT828903	
10	CD5ATA	*Caulobacter* sp. D5	pET-28a[5]	WP_110132 869.1	19
11	*CD5*ATA-D44E/Y138F/P192Q/T377V/A448R (*CD5*ATA_M1)		pET-28a	N/A	20
12	ATA-117-Rd11	*Arthrobacter* sp. KNK168	pET-228b	3WWJ_A	7,8
13	*Cvi*TA-Y88L (*Cvi*TA_M1)	*Chromobacterium vio-laceum*	pET-28a	N/A	In house
14	*Cvi*TA-Y88L/D418L (*Cvi*TA_M2)		pET-28a	N/A	In house
15	TA-v2	*Pseudomonas* sp. FP2335	pET-28a	N/A	21
16	*At*TA-F115H/E117C (*At*TA_M1)	*Aspergillus terreus*	pET-28a	N/A	13
17	*At*TA-H55Y/F115H/E117C (*At*TA_M2)			N/A	
18	*At*TA-H55R/F115H/E117C (*At*TA_M3)			7XG5	
**Entry**	**KRED**	**Source**	**Vector**	**Accession**	**Reference**
19	*Fs*KRED	*Flavobacterium* sp.	pET-28a		22
20	*Fp*KRED	*Flavobacterium psychro-philum*	pET-28a		
21	*Ls*KRED	*Leifsonia* sp.	pET-28a		23, 24
22	*Lk*KRED	*Lactobacillus kefir*	pET-28b		25
23	*Lb*KRED	*Lactobacillus brevis*	pEG-180		26, 27
24	KRED-NADH-101	N/A	N/A	N/A	Codexis, Inc. (Redwood City, US)

2.2. Nucleotide and amino acid sequences of the biocatalysts used in the final setup of this study.

3HMU nucleotide sequence (restriction sites *Nde*I/*Bam*HI are not shown)

ATGAGCCTGGCGACCATTACGAACCACATGCCGACGGCGGAACTGCAAGCCCTGGATGCTGCCCACCACCTGCAC
CCGTTTAGCGCAAACAATGCACTGGGTGAAGAAGGCACCCGTGTTATTACGCGTGCTCGCGGTGTCTGGCTGAAC
GATAGCGAAGGCGAAGAAATTCTGGACGCCATGGCAGGTCTGTGGTGCGTCAATATCGGTTATGGTCGTGATGAA
CTGGCAGAAGTGGCAGCACGTCAGATGCGTGAACTGCCGTATTACAACACCTTTTTCAAAACCACGCATGTTCCG
GCTATTGCGCTGGCCCAAAAACTGGCAGAACTGGCTCCGGGCGATCTGAATCACGTGTTTTTCGCCGGCGGTGGC
AGCGAAGCAAACGACACCAATATCCGTATGGTGCGCACGTATTGGCAGAACAAAGGTCAACCGGAAAAAACCGTT
ATTATCAGCCGTAAAAATGCGTACCATGGCTCTACGGTCGCAAGCTCTGCACTGGGTGGCATGGCTGGTATGCAC
GCGCAGTCTGGCCTGATTCCGGATGTGCATCACATCAACCAACCGAATTGGTGGGCCGAAGGTGGCGATATGGAC
CCGGAAGAATTTGGTCTGGCACGTGCTCGCGAACTGGAAGAAGCAATTCTGGAACTGGGTGAAAACCGTGTGGCA
GCTTTCATTGCTGAACCGGTTCAGGGTGCGGGTGGCGTGATCGTTGCACCGGATTCATATTGGCCGGAAATTCAA
CGCATCTGCGATAAATACGACATTCTGCTGATCGCGGACGAAGTTATTTGTGGTTTTGGCCGTACCGGTAATTGG
TTCGGCACCCAGACGATGGGTATCCGCCCGCATATTATGACGATCGCAAAAGGTCTGAGTTCCGGCTATGCTCCG
ATTGGTGGCTCAATCGTGTGTGATGAAGTCGCACACGTGATTGGCAAAGACGAATTTAACCATGGTTATACCTAC
TCGGGTCACCCGGTGGCAGCAGCAGTTGCACTGGAAAATCTGCGTATTCTGGAAGAAGAAAACATCCTGGATCAT
GTTCGCAATGTCGCTGCGCCGTATCTGAAAGAAAAATGGGAAGCACTGACCGACCACCCGCTGGTCGGTGAAGCC
AAAATTGTGGGCATGATGGCATCCATCGCTCTGACCCCGAACAAAGCGTCACGCGCCAAATTTGCATCGGAACCG
GGTACGATTGGCTACATCTGCCGTGAACGCTGTTTCGCGAACAATCTGATTATGCGTCATGTCGGCGATCGCATG
ATTATCAGTCCGCCGCTGGTGATTACCCCGGCCGAAATCGATGAAATGTTCGTTCGTATCCGCAAATCCCTGGAC
GAAGCGCAGGCCGAAATTGAAAAACAGGGTCTGATGAAATCTGAAGGTAGTCACCACCACCACCACCACTAA

3HMU amino acid sequence

MSLATITNHMPTAELQALDAAHHLHPFSANNALGEEGTRVITRARGVWLNDSEGEEILDAMAGLWCVNIGYGRDE
LAEVAARQMRELPYYNTFFKTTHVPAIALAQKLAELAPGDLNHVFFAGGGSEANDTNIRMVRTYWQNKGQPEKTV
IISRKNAYHGSTVASSALGGMAGMHAQSGLIPDVHHINQPNWWAEGGDMDPEEFGLARARELEEAILELGENRVA
AFIAEPVQGAGGVIVAPDSYWPEIQRICDKYDILLIADEVICGFGRTGNWFGTQTMGIRPHIMTIAKGLSSGYAP
IGGSIVCDEVAHVIGKDEFNHGYTYSGHPVAAAVALENLRILEEENILDHVRNVAAPYLKEKWEALTDHPLVGEA
KIVGMMASIALTPNKASRAKFASEPGTIGYICRERCFANNLIMRHVGDRMIISPPLVITPAEIDEMFVRIRKSLD
EAQAEIEKQGLMKSEGSHHHHHH

***Cvi*_TA nucleotide sequence (restriction sites *Nco*I/*Hind*III are not shown)**

ATGGGCAGCAGCCATCATCATCATCATCACAGCAGCGGCCTGGTGCCGCGCGGCAGCCATATGGCTAGCCAGAAA
CAGCGTACCACCTCTCAGTGGCGTGAACTGGATGCAGCACATCATCTGCATCCGTTTACCGATACCGCAAGCCTG
AATCAGGCAGGTGCACGTGTTATGACCCGTGGTGAAGGTGTTTATCTGTGGGATAGCGAAGGCAACAAAATTATT
GATGGTATGGCAGGTCTGTGGTGTGTTAATGTTGGTTATGGTCGCAAAGATTTTGCAGAAGCAGCACGTCGTCAG
ATGGAAGAACTGCCGTTTTATAATACCTTTTTTAAAACCACCCATCCGGCAGTTGTTGAACTGAGCAGCCTGCTG
GCCGAAGTTACACCGGCAGGTTTTGATCGTGTGTTTTATACCAATAGCGGTAGCGAAAGCGTTGATACCATGATT
CGCATGGTTCGTCGTTATTGGGATGTTCAGGGCAAACCGGAAAAAAAAACCCTGATCGGTCGTTGGAATGGTTAT
CATGGTAGCACCATTGGTGGTGCCAGCCTGGGTGGTATGAAATATATGCATGAACAGGGTGATCTGCCGATTCCG
GGTATGGCACATATTGAACAGCCGTGGTGGTATAAACATGGCAAAGATATGACACCGGATGAATTTGGTGTTGTT
GCAGCACGTTGGCTGGAAGAAAAAATTCTGGAAATTGGTGCCGATAAAGTTGCAGCATTTGTGGGTGAACCGATT
CAGGGTGCAGGTGGTGTTATTGTTCCGCCTGCAACCTATTGGCCTGAAATTGAACGTATCTGCCGCAAATATGAT
GTTCTGCTGGTTGCCGATGAAGTTATTTGTGGTTTTGGTCGTACCGGTGAATGGTTTGGTCATCAGCATTTTGGT
TTTCAGCCGGACCTGTTTACCGCAGCCAAAGGCTTATCTTCTGGCTATCTGCCGATTGGTGCAGTTTTTGTTGGT
AAACGTGTTGCAGAAGGTCTGATTGCAGGCGGTGATTTTAATCATGGCTTTACCTATAGCGGTCATCCGGTTTGT
GCAGCAGTTGCACATGCAAATGTTGCAGCACTGCGTGATGAAGGTATTGTTCAGCGCGTGAAAGATGATATTGGT
CCGTATATGCAGAAACGTTGGCGTGAAACCTTTAGCCGTTTTGAACATGTTGATGATGTTCGTGGTGTTGGTATG
GTTCAGGCATTTACCCTGGTGAAAAACAAAGCAAAACGCGAACTGTTTCCGGATTTTGGTGAAATTGGCACCCTG
TGCCGTGATATTTTTTTTCGCAATAATCTGATTATGCGTGCCTGTGGTGATCACATTGTTAGCGCACCGCCTCTG
GTGATGACCCGTGCCGAAGTTGATGAAATGCTGGCCGTTGCAGAACGCTGTCTGGAAGAATTTGAACAGACCCTG
AAAGCACGTGGTCTGGCCTAA

_Cvi__TA amino acid sequence

MGSSHHHHHHSSGLVPRGSHMASQKQRTTSQWRELDAAHHLHPFTDTASLNQAGARVMTRGEGVYLWDSEGNKII
DGMAGLWCVNVGYGRKDFAEAARRQMEELPFYNTFFKTTHPAVVELSSLLAEVTPAGFDRVFYTNSGSESVDTMI
RMVRRYWDVQGKPEKKTLIGRWNGYHGSTIGGASLGGMKYMHEQGDLPIPGMAHIEQPWWYKHGKDMTPDEFGVV
AARWLEEKILEIGADKVAAFVGEPIQGAGGVIVPPATYWPEIERICRKYDVLLVADEVICGFGRTGEWFGHQHFG
FQPDLFTAAKGLSSGYLPIGAVFVGKRVAEGLIAGGDFNHGFTYSGHPVCAAVAHANVAALRDEGIVQRVKDDIG
PYMQKRWRETFSRFEHVDDVRGVGMVQAFTLVKNKAKRELFPDFGEIGTLCRDIFFRNNLIMRACGDHIVSAPPL
VMTRAEVDEMLAVAERCLEEFEQTLKARGLA

ATA-117-Rd11 nucleotide sequence (restriction sites _Nde_I/_Bam_HI are not shown)

ATGGCATTCAGCGCAGATACGCCGGAAATCGTCTACACCCATGACACGGGCCTGGACTACATTACCTACTCGGAC
TACGAACTGGACCCGGCAAACCCGCTGGCTGGCGGTGCAGCATGGATTGAGGGTGCGTTTGTGCCGCCGAGTGAA
GCCCGTATTTCCATCTTTGATCAGGGTTTCTATACGTCTGACGCAACCTACACCACGTTTCATGTTTGGAACGGT
AATGCTTTCCGTCTGGGCGACCACATTGAACGCCTGTTCAGCAATGCAGAATCTATTCGCCTGATCCCGCCGCTG
ACGCAAGATGAAGTCAAAGAAATCGCGCTGGAACTGGTGGCCAAAACCGAACTGCGTGAAGCCATGGTCACCGTG
ACGATTACCCGCGGCTATAGCTCTACGCCGTTTGAACGTGATATCACCAAACATCGCCCGCAGGTGTATATGAGT
GCGTGCCCGTACCAATGGATTGTTCCGTTCGATCGTATCCGCGACGGTGTGCACCTGATGGTTGCACAGAGCGTC
CGTCGCACCCCGCGTAGTTCCATTGATCCGCAGGTGAAAAACTTTCAATGGGGCGACCTGATTCGTGCAATCCAA
GAAACCCATGATCGCGGTTTCGAACTGCCGCTGCTGCTGGATTGTGACAACCTGCTGGCTGAAGGTCCGGGCTTT
AATGTGGTTGTCATCAAAGATGGTGTGGTTCGTAGCCCGGGTCGTGCAGCTCTGCCGGGTATTACGCGCAAAACC
GTTCTGGAAATCGCGGAATCTCTGGGCCACGAAGCGATTCTGGCCGATATCACGCCGGCAGAACTGTACGATGCT
GACGAAGTTCTGGGTTGCTCAACCGGCGGTGGCGTCTGGCCGTTCGTTTCGGTCGATGGTAATTCAATTTCGGAC
GGTGTGCCGGGTCCGGTTACCCAGAGCATTATCCGTCGCTACTGGGAACTGAATGTTGAACCGTCATCGCTGCTG
ACCCCGGTCCAATACACTAA

ATA-117-Rd11 amino acid sequence

MAFSADTPEIVYTHDTGLDYITYSDYELDPANPLAGGAAWIEGAFVPPSEARISIFDQGFYTSDATYTTFHVWNG
NAFRLGDHIERLFSNAESIRLIPPLTQDEVKEIALELVAKTELREAMVTVTITRGYSSTPFERDITKHRPQVYMS
ACPYQWIVPFDRIRDGVHLMVAQSVRRTPRSSIDPQVKNFQWGDLIRAIQETHDRGFELPLLLDCDNLLAEGPGF
NVVVIKDGVVRSPGRAALPGITRKTVLEIAESLGHEAILADITPAELYDADEVLGCSTGGGVWPFVSVDGNSISD
GVPGPVTQSIIRRYWELNVEPSSLLTPVQYTDPNSSSVDKLAAALEHHHHHH

ATA-117 nucleotide sequence (restriction sites _Nde_I/_Bam_HI are not shown)

ATGGCTTTTTCAGCGGACACCTCGGAAATTGTTTACACCCACGATACCGGCCTGGATTACATTACCTACTCGGAC
TACGAACTGGACCCGGCAAACCCGCTGGCCGGCGGTGCAGCATGGATTGAAGGTGCATTTGTGCCGCCGAGTGAA
GCTCGTATTTCCATCTTCGATCAGGGCTATCTGCATTCTGACGTTACCTACACGGTGTTTCACGTTTGGAACGGT
AATGCTTTCCGTCTGGATGACCATATTGAACGCCTGTTTAGCAATGCAGAATCTATGCGCATTATCCCGCCGCTG
ACCCAAGATGAAGTGAAAGAAATCGCACTGGAACTGGTTGCTAAAACGGAACTGCGTGAAGCCTTCGTCTCAGTG
TCGATTACCCGCGGCTATAGCTCTACGCCGGGTGAACGTGATATCACCAAACATCGCCCGCAGGTTTATATGTAC
GCGGTCCCGTACCAATGGATTGTGCCGTTTGATCGTATCCGCGACGGTGTCCACGCAATGGTGGCACAGAGCGTT
CGTCGCACCCCGCGTAGTTCCATTGATCCGCAGGTGAAAAACTTTCAATGGGGTGACCTGATCCGTGCGGTTCAA
GAAACGCATGATCGCGGCTTCGAAGCACCGCTGCTGCTGGATGGTGACGGTCTGCTGGCAGAAGGCAGCGGTTTC
AATGTGGTTGTCATTAAAGACGGTGTGGTTCGTTCTCCGGGTCGTGCAGCTCTGCCGGGTATTACCCGCAAAACG
GTGCTGGAAATCGCCGAAAGTCTGGGTCACGAAGCAATTCTGGCTGATATCACCCTGGCGGAACTGCTGGATGCC
GACGAAGTCCTGGGTTGCACCACGGCGGGCGGTGTTTGGCCGTTTGTTTCAGTCGATGGCAACCCGATTTCGGAC
GGTGTCCCGGGTCCGGTGACCCAGAGCATCATCCGCCGCTACTGGGAACTGAATGTTGAATCATCGTCGCTGCTG
ACCCCGGTGCAATACTAA

ATA-117 amino acid sequence

MAFSADTSEIVYTHDTGLDYITYSDYELDPANPLAGGAAWIEGAFVPPSEARISIFDQGYLHSDVTYTVFHVWNG
NAFRLDDHIERLFSNAESMRIIPPLTQDEVKEIALELVAKTELREAFVSVSITRGYSSTPGERDITKHRPQVYMY
AVPYQWIVPFDRIRDGVHAMVAQSVRRTPRSSIDPQVKNFQWGDLIRAVQETHDRGFEAPLLLDGDGLLAEGSGF
NVVVIKDGVVRSPGRAALPGITRKTVLEIAESLGHEAILADITLAELLDADEVLGCTTAGGVWPFVSVDGNPISD
GVPGPVTQSIIRRYWELNVESSSLLTPVQY

***Fs*KRED nucleotide sequence (restriction sites *Nde*I/*Xho*I not shown)**

ATGAACTTCACCGATAAAAATATTATCATCACCGGTGGTAGTGCAGGCATTGGTCTGGCAACCGCAAAAACCTTC
ATTGCCAAAGGCGCAAATGTGCTGATTACCGGTCGTAATACCAATAGCCTGCAGACCGCCAGCGAAAAAATTAAT
AGTCCGAAACTGAAAACCTTAGCAAGCGATATTAGTAAACTGGAAGATATTGCCACCCTGGAAAAAGAAATTGCC
GAAAGCGGCAATAAAGTTGATGTGCTGGTTCTGAATGCAGGTATTGCCAAACAGTTCAGTATTGAAGAAACCACC
GAAGAAGTGTTCGATGATCTGTTCAATATTAATGTTAAGGGTCTGTTCTTCACCCTGCAGAAACTGATTCCGCAT
CTGGCAGAAGGTGCCAGCGTTATTCTGATTAGCAGCGGCGTGAGTGTGAGCGGCTATGCACAGATGGGTGCATAC
GCTGCCACCAAAAGCGCAGTTGATGCCATTGCACGTACCGCCGCCATTGAACTGGCAGATCGCAAAATTCGCGTG
AATACCGTGGCCCCGGGTCTGACCGATACCCCGATGAATCATCAGACCCCGGAAGATATTAAAAATGCCATTGCA
GCCGCAGTTCCGCTGAAACGTATTGGCGAAGCAGAAGAAATTGCAAATGCCATTGTGTTCCTGGCCAGTGAAGAA
GCCAGTTATATTAGTGGTAGCTATCTGAGCGTTGATGGCGGCGTTACCATTCGCCGCTAA

***Fs*KRED amino acid sequence**

MGSSHHHHHHSSGLVPRGSHMNFTDKNIIITGGSAGIGLATAKTFIAKGANVLITGRNTNSLQTASEKINSPKLK
TLASDISKLEDIATLEKEIAESGNKVDVLVLNAGIAKQFSIEETTEEVFDDLFNINVKGLFFTLQKLIPHLAEGA
SVILISSGVSVSGYAQMGAYAATKSAVDAIARTAAIELADRKIRVNTVAPGLTDTPMNHQTPEDIKNAIAAAVPL
KRIGEAEEIANAIVFLASEEASYISGSYLSVDGGVTIRR

***Ls*KRED nucleotide sequence (restriction sites *Nde*I/*Xho*I not shown)**

ATGGCACAGTATGATGTTGCAGATCGTAGCGCAATTGTTACCGGTGGTGGTAGCGGTATTGGTCGTGCAGTTGCA
CTGACCCTGGCTGCAAGCGGTGCAGCAGTTCTGGTTACCGATCTGAATGAAGAACATGCACAGGCAGTTGTTGCA
GAAATTGAAGCAGCAGGCGGAAAAGCCGCTGCACTGGCTGGTGATGTTACCGATCCGGCATTTGGTGAAGCAAGC
GTTGCAGGTGCAAATGCACTGGCTCCGCTGAAAATTGCAGTTAACAATGCCGGTATTGGTGGTGAAGCAGCAACC
GTTGGTGATTACAGCCTGGATAGCTGGCGTACCGTTATTGAAGTTAATCTGAATGCCGTGTTTTATGGTATGCAG
CCGCAGCTGAAAGCAATGGCAGCAAATGGTGGTGGTGCCATTGTTAATATGGCAAGCATTCTGGGTAGCGTTGGT
TTTGCAAATAGCAGCGCCTATGTGACCGCAAAACATGCACTGCTGGGTCTGACACAGAATGCAGCACTGGAATAT
GCAGCAGATAAAGTTCGTGTTGTTGCAGTTGGTCCGGGTTTTATTCGTACACCGCTGGTTGAAGCAAATCTGAGC
GCAGATGCACTGGCCTTTCTGGAAGGTAAACATGCCCTGGGTCGTCTGGGTGAACCGGAAGAAGTTGCAAGCCTG
GTTGCATTTCTGGCCTCTGATGCAGCAAGCTTTATTACCGGTAGCTATCATCTGGTTGATGGTGGTTATACCGCA
CAGTAA

***Ls*KRED amino acid sequence**

MAQYDVADRSAIVTGGGSGIGRAVALTLAASGAAVLVTDLNEEHAQAVVAEIEAAGGKAAALAGDVTDPAFGEAS
VAGANALAPLKIAVNNAGIGGEAATVGDYSLDSWRTVIEVNLNAVFYGMQPQLKAMAANGGGAIVNMASILGSVG
FANSSAYVTAKHALLGLTQNAALEYAADKVRVVAVGPGFIRTPLVEANLSADALAFLEGKHALGRLGEPEEVASL
VAFLASDAASFITGSYHLVDGGYTAQ

2.3. Transaminase gene expression in deep-well-plates (DWP) and cell-free extract preparation for activity screening

Pre-cultures of all tested enzymes were prepared in DWP by inoculating 1 mL of Lysogeny Broth (LB) medium, containing the respective antibiotic, with cell material from a glycerol stock of the desired *Escherichia coli* BL21(DE3) transformant. The cultures were allowed to grow overnight at 30°C, 400 rpm. *E. coli* BL21(DE3) harboring the respective empty plasmid, served as a reference in control reactions. Expression was performed in 24-well deep well plates (DWP) from BL21(DE3) cells and in 5 mL terrific broth (TB) per well. The medium was supplemented with ampicillin to the final concentration of 100 µg/mL) or kanamycin to the final concentration of 50 µg/mL, and then inoculated with 50 µL of the overnight culture. Cultures were incubated for 3 h at 37 °C and 400 rpm in an incubator shaker (Incubator Hood TH 30, Edmund Bühler GmbH, Germany). Induction was done with isopropyl-β-D-thiogalactoside (IPTG) to the final concentration of 1 mM or L-rhamnose to the final concentration of 10 mM), followed by incubation at 20 °C overnight. Next, cells were harvested (2,500 *g,* 40 min, 4 °C), washed with buffer (50 mM HEPES, pH 7.5, 150 mM NaCl), and pelleted again under the same conditions.
For cell lysis, 1 mL of lysis buffer per well (0.1 mM PLP, 1 mg/mL lysozyme, 1 µg/mL DNase, 3 mg/mL PMSF in 50 mM HEPES, pH 7.5) was used for cell pellets' resuspension. The plate was then placed again in the shaker and incubated for 90 min at 30 °C and 600 rpm. Afterwards, the plate was centrifuged (2,500 *g* 40 min, 4 °C) to remove cell debris, and each supernatant (cell-free extract) was collected separately and stored at 4°C until further use within the same day.

2.4. Expression of selected TAs and KREDs in culture flasks

The selected TA and KRED constructs were expressed in BL21 (DE3) cells, which were grown in baffled flasks containing 20-50 or 300 mL of TB medium for small or large-scale expression, respectively. Media were supplemented with ampicillin or kanamycin, as described above, and then inoculated with the overnight culture to a final 1% v/v concentration. The cells were incubated at 37 °C and 300 rpm (Multitron, Infors HT) until an OD_{600} of 0.6-0.8, and then gene expression was induced with IPTG at a final concentration of 0.5 mM. The cultures were incubated at 20 °C overnight and harvested (4,000 x *g,* 30 min, 4 °C) 20 h post-induction.

The obtained cell pellets were washed with HEPES buffer (50 mM HEPES, pH 7.5, 150 mM NaCl) in case of TAs, and phosphate buffer (50 mM sodium phosphate, pH 7.5) in case of KREDs and pelleted again under the same conditions. Further processing followed according to the aimed use, as described below.

2.5. Crude cell extract preparation from shake flask cultures

Cell pellets were resuspended in lysis buffer (0.1 mM PLP, 1 mg/mL lysozyme, 1 µg/mL DNase, 3 mg/mL PMSF in 50 mM HEPES, pH 7.5 in case of TAs, or 1 mg/mL lysozyme, 1 µg/mL DNase, 3 mg/mL PMSF in 50 mM phosphate buffer, pH 7.5 in case of KREDs), to an OD_{600} of 10, with the following steps being as described above. The lysate was centrifuged (4000 x *g,* 40 min, 4 °C), and the supernatant (crude cell extract) was collected and aliquoted in brown 1.5 mL Eppendorf tubes. The extracts were stored at -20 °C until further use.

2.6. Resting cells preparation

Harvested cells were resuspended in HEPES buffer (50 mM HEPES, 1 mM PLP, pH 8) in case of TAs or 50 mM phosphate buffer, pH 7 in case of KREDs, to an OD_{600} 10 or 20.

2.7. Whole cell lyophilisates preparation

Harvested cells were resuspended (0.5 g wet cell pellet/10 mL buffer) in 50 mM HEPES buffer, pH 8 (TAs) or 50 mM sodium phosphate buffer, pH 7 (KREDs) and stored at -80 °C for at least 4 h. The frozen cell suspension was lyophilized for 24 h at 0.1 mbar and -40 °C. The resulting dried cells were stored at -20 °C until further use.

2.8. Estimation of protein concentration in crude cell extracts and estimation of expression levels

The protein concentration in the prepared crude cell extracts was estimated using NanoDrop® (Thermo Fisher Scientific GmbH, Germany, Dreieich) and the respective MW and extinction coefficient was calculated based on ProtParam.[28] Expression levels were verified by sodium dodecyl sulfate-polyacrylamide gel electrophoresis (SDS-PAGE).

3. Activity assays

3.1. Measurement of the specific activity of transaminases using the acetophenone assay

The specific activity of ATA towards the substrate(s) of interest, as well as under different operational conditions, was studied using the acetophenone assay, which was adapted from Schätzle et al.[30] and was applied on crude cell extracts for this study. The assay was performed using the microplate reader and UV-transparent microplate plates. To each monitored well, 198 µL of the master mix (2.5 mM (*S*)- or (*R*)-PEA, 2.5 mM pyruvate or substrate of interest, 0.1 mM PLP in 50 mM HEPES buffer, pH 8) was added. The final DMSO concentration was 0.24% (v/v) in case of pyruvate or 1% (v/v) in case of other amine acceptor substrates. The plate was preincubated at 30 °C for 5 min, before addition of 2 µL of the crude cell extract per well. The plate was shaken for 5 s and then inserted to the microplate reader. Absorbance increase at 245 nm correlating to acetophenone level increase was recorded for 1 h (at 10 s intervals) at 30 °C. The following controls were used: sample not containing crude cell extract (only lysis buffer, blank), sample containing crude cell extract from cells transformed with the empty plasmid, sample not containing amine acceptor. One unit (U) activity was defined as the formation of 1 µmol formed acetophenone per minute. All measurements were performed in triplicates.

3.2. Measurement of transaminase specific activity under different pH- and solvent conditions

For a fast screening of TA activity under different pH- or solvent conditions, the enzyme-containing cell extracts were added without prior incubation in the tested condition.
To determine the activity of the transaminases at different pH values, the assay was performed as described in 3.1 using pyruvate as amino acceptor, but in a buffer of a pH ranging from 5 to 10 (50 mM HEPES buffer). The pH was adjusted with addition of 2M HCl or 5M NaOH.
To assess the activity of the transaminases in the presence of different co-solvents in water (acetonitrile, methanol, DMSO), biocatalytic reactions were set up as described in chapter 5.1.1., but instead of DMSO, they contained the respective co-solvent at a varying percentage.

3.3. Measurement of ketoreductase specific activity using the NAD(P)H assay

Ketoreductase specific activity towards the substrate(s) of interest, as well as under different operational conditions, was studied using the NAD(P)H assay, which was adapted by Truppo et al.[30], and applied on crude cell extracts for this study. The assay was performed using the microplate reader and 96-well polystyrene microplates (as mentioned in section 2.1). To each monitored well, 198 µL of the master mix (0.1 mM NAD(P)H and 1 mM substrate of interest dissolved in 50 mM phosphate buffer, pH 7.5) was added. The final DMSO concentration was 0.4 % (v/v). The plate was preincubated at 30 °C for 5 min, before addition of 2 µL of the respective crude cell extracts or Codexis KRED enzymes (from 1 mg/mL stocks in 50 mM sodium phosphate buffer, pH 7). The plate was shaken for 5 s and then inserted to the microplate reader. Absorbance decrease at 340 nm correlating to NAD(P)H level decrease was recorded for 1 h (in 10 s intervals) at 30 °C. The following controls were used: a sample not containing crude cell extract/lyophilized enzyme (only lysis buffer, blank), a sample containing crude cell extract cells transformed with the empty plasmid, a sample not containing amine acceptor. The enzyme activity (U/mL) is defined as the amount of enzyme that catalyzes the oxidation of 1 µmol NAD(P)H per minute per mL reaction volume. All measurements were performed in triplicates.

3.4. Measurement of ketoreductase specific activity under different pH- and solvent conditions

For a fast screening of KRED activity under different pH- or solvent conditions, the enzyme-containing cell extracts were added without prior incubation in the tested condition.
To determine KRED activity at different pH values, the assay was performed as described in 3.3, using the well-accepted substrate anisole, but in a buffer of a pH ranging from 5 to 10 (50 mM phosphate buffer). The pH was adjusted with addition of 2 M HCl or 5 M NaOH.
To assess KRED activity in the presence of different co-solvents in water (acetonitrile, methanol, DMSO), biocatalytic reactions were setup as described in 5.2.1., but instead of DMSO, they contained the respective co-solvent at a varying percentage.

4. Chemical synthesis workflows

4.1. Synthesis of sodium decatungstate

Sodium decatungstate was synthesized as described in Sarver et al.[31]

4.2. Oxyfunctionalization of remote C-H bonds of aliphatic amines via decatungstate photocatalysis

The synthesis of the oxyfunctionalized amines was performed via modification of the procedure described in Schultz et al. (Fig. S2).[33]

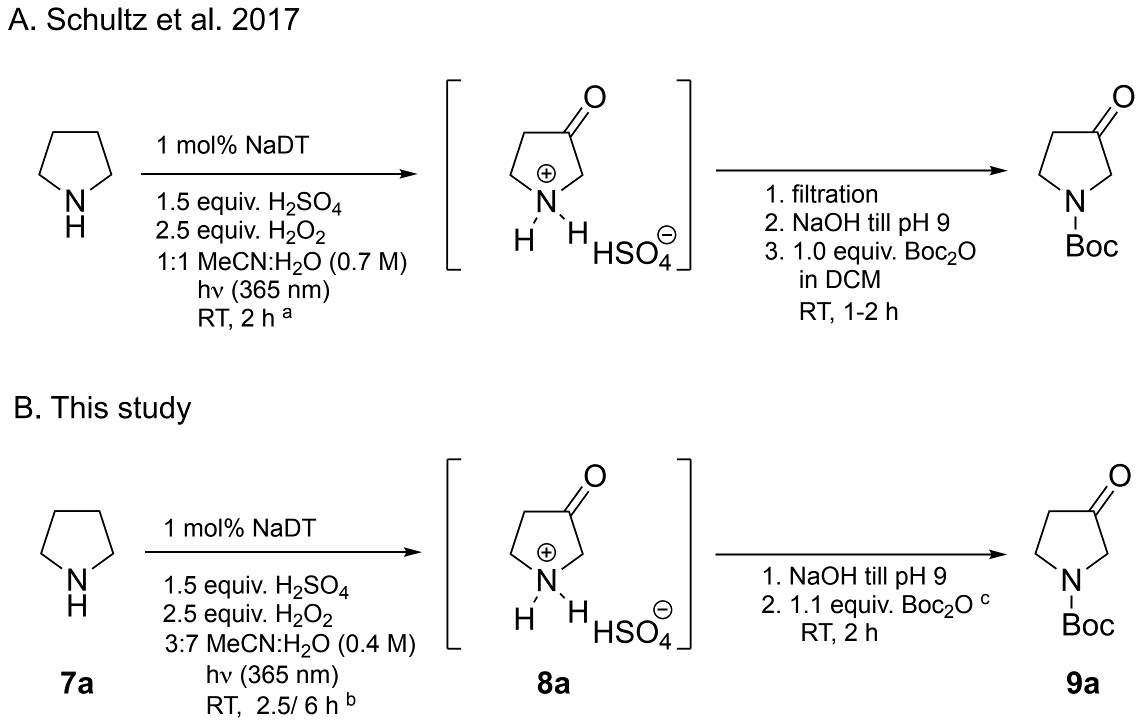

Figure S2. Photooxyfunctionalization of aliphatic amines at remote C-H bonds and following *N*-protection via Boc_2O addition. The pyrrolidine substrate (**7a**) is shown indicatively. **A.** Conditions developed by Schultz et al.[32]; the photooxyfunctionalization step takes places in 50% MeCN in water, whereas Boc_2O is added as a solution in DCM, upon filtration and basification of the crude intermediate **8a** in a second reaction pot. **B.** Conditions optimized for this study; the photooxyfunctionalization step takes places in 30% MeCN in water, the *N*-protection step takes place in same pot, without filtration and upon basification of the crude intermediate **8a,** by adding 1.1 equiv. of solvent-free Boc_2O.

[a] no total reaction volume/substrate mass is mentioned.

[b] 2.5 h for 0.1 mmol-setup during which 8 mL-clear glass vials were used, 5 h for 1.4 mmol setup, during which 22 mL-clear glass vials were used. In general, it was observed, that reaction duration also depended notably on reaction-vial diameter, with reaction time increasing as vial diameter increased.

[c] solvent-free.

4.2.1. Analytical scale (0.07 mmol)

For establishing the photobiocatalytic reaction and investigating conditions, the photo-oxyfunctionalization reaction was first set up at analytical scale. In this case, a 22 mL Wheaton sample vial with a pressure release cap was charged with the amine (99.4 mM, 0.07 mmol, 1 equiv.) and MeCN/water in a 3:10 (v/v) ratio. While keeping on ice, H_2SO_4 (1.5 equiv.) was added dropwise resulting in a slight temperature rise. The vial was then charged with NaDT (1.71 mg, 1 mol%) and aqueous H_2O_2 (30% solution,

2.5 equiv.) before being equipped with a stir bar and sealed. Finally, the vial was irradiated at 365 nm until the color of the reaction mixture turned intense clear blue, indicating deactivation of the catalyst and end of the reaction. Reaction was complete within 3 h. As a next step, the vial was removed from the photoreactor and while keeping on ice, the crude reaction was neutralized and basified (to pH 9) with NaOH (10 M stock, 0.7 equiv.). Liquid Boc_2O (1.1 equiv.) was added to the crude mixture and vortexed. The vial was flushed with Argon for 5 s, sealed and covered with aluminium foil, before being left to stir on a magnetic stirrer for 1.5-2 h at RT and 600 rpm.

4.2.2. Preparative scale (1.42 mmol)

A 22 mL Wheaton sample vial with a pressure release cap was charged with the amine substrate (400 mM, 1.42 mmol, 1 equiv.) and MeCN/water in a 3:10 (v/v) ratio. While keeping on ice, H_2SO_4 (1.5 equiv) was added dropwise resulting in a slight in a slight temperature rise. The vial was then charged with NaDT (32.4 mg, 1 mol%) and aqueous H_2O_2 (30% solution, 2.5 equiv) before being equipped with a stir bar and sealed. Finally, the vial was irradiated at 365 nm until the color of the reaction mixture turned intense clear blue, indicating deactivation of the catalyst and end of the reaction. Reaction was complete within 5-6 h.
As a next step, the vial was removed from the lamp and while keeping on ice, the crude reaction was neutralized and basified (to pH 9) with NaOH (10 M stock, 0.7 equiv.). Liquid boc-anhydride (1.1 equiv.) was added to the crude mixture and vortexed. The vial was flushed with Argon for 5 s, sealed and covered with aluminium foil, before being placed on a magnetic stirrer for 2 h at RT and 600 rpm (**Fig. S3**).

4.2.3. Extraction before GC-analysis

Once the amine protection step was complete, the crude reaction was further basified with 10 M NaOH until a pH of 12, followed by extraction with equal volume of extraction solvent (ethyl acetate containing 1 mM dodecane as internal standard). The organic layer was collected and dried with anhydrous $MgSO_4$. After centrifugation (13,000 *g*, 12 min, RT), the supernatant was diluted 1:10 with the extraction solvent and finally analyzed with GC-MS or GC-FID.

4.2.4. Extraction of the preparative-scale reaction for ketone product isolation

Once the amine protection step was complete, the crude reaction was further basified with 10 M NaOH until a pH of 12 and extracted thrice with equal volume of ethyl acetate. The organic layers were pooled together and washed twice with brine. The resulting organic phase was additionally dried with anhydrous $MgSO_4$, followed by filtering through a 0.22 um Whatman filter, and solvent removal *in vacuo.* Purification of the crude product was performed via silica flash column chromatography using a gradient of ethyl acetate in hexane (0-30% v/v). Fractions containing the product of interest were identified by TLC analysis and pooled. After concentration under reduced pressure, the isolated yield was determined by weight and product identity by NMR-spectroscopy.

5. Biocatalytic synthesis workflows

For reaction establishment and activity studies, the reactions were first performed in 1.5-mL (brown) Eppendorf tubes at 0.5 mL total reaction volume. The ketone substrate was added from a 500-fold concentrated stock solution in DMSO. The final DMSO concentration in the transamination and ketoreduction reactions, was 2% and 0.4% (v/v), respectively.

5.1. Biocatalytic transamination of *N*-Boc-3-pyrrolidinone (9a) and *N*-Boc-hexahydro-1*H*-azepin-4-one (9b)

5.1.1. Reaction setup with cell-free extracts

An Eppendorf tube was charged with HEPES buffer (50 mM HEPES, 1 M isopropylamine (IPA), pH adjusted with 2 M HCl to pH 8, and 1 mM PLP). The respective crude cell extract was added to a final concentration of 0.1 mg protein/ mL reaction volume, before the addition of the substrate to a final concentration of 20 mM. Reactions were incubated for 20 h at 30°C and 600 rpm in an Eppendorf ThermoMixer® C device (Eppendorf SE, Germany, Hamburg).

5.1.2. Reaction setup with resting cells

In a 1.5-mL Eppendorf tube, HEPES buffer (50 mM HEPES,1 M IPA pH=8, pH-adjusted with 2 M HCl, and 1 mM PLP) was mixed in a 1:1 ratio with the respective resting-cell suspension to a final OD_{600} of 5 or 10. The ATA-containing cell suspension had been prepared as described in 2.4. Finally, the substrate was added to a final concentration of 20 mM and reactions were incubated for 20 hours at 30°C and 600 rpm in the thermomixer.

5.1.3. Reaction setup with dried whole cells

In a 1.5-mL Eppendorf tube, a cell suspension deriving from resuspension of lyophilized cells in HEPES buffer (50 mM HEPES,1 M IPA pH=8, pH-adjusted with 2 M HCl, and 1 mM PLP) to a final concentration of 15 mg/mL, was added. The ATA-containing dried cells had been prepared as described in 2.5. Finally, the substrate was added to a final concentration of 20 or 50 mM. Reactions were incubated for 20 h, at 30 °C and 600 rpm, in a thermomixer. For coupling to the chemocatalytic step, the optimized conditions contained 5% MeCN instead of 2 % (v/v) DMSO.

5.2. Biocatalytic ketone reduction of 9a and 9b

5.2.1. Reaction setup with cell-free extracts

A 1.5 mL- Eppendorf tube was charged with 50 mM phosphate buffer pH 7 and the following components at the given final concentrations: 0.1 mM NADP, 25 mM D-glucose and 10 U GDH. The respective crude cell extract was added to a final concentration of 0.5 mg protein/ mL reaction volume, before the addition of the substrate to a final concentration of 20 mM in a 0.5 mL total reaction volume. Reactions were incubated for 20 h at 30°C and 600 rpm in an Eppendorf ThermoMixer® C device (Eppendorf SE, Germany, Hamburg).

5.2.2. Reaction setup with resting cells

Harvested cells containing *Fp*KRED, *Lb*KRED, *Lk*KRED or *Fs*KRED were resuspended in 50 mM phosphate buffer pH 7 to an OD_{600} of 10, and glucose was added to a final concentration of 10 % (v/v), following a 1 h incubation on ice. The cell suspension was distributed in 1.5-mL Eppendorf tubes per 0.5 mL, and substrate was added to a final concentration of 20 mM. Reactions were incubated for 20 h, at 30 °C and 600 rpm, in a thermomixer.

5.2.3. Reaction setup with dried whole cells

Lyophilized cells containing *Ls*KRED or *Fs*KRED were resuspended in 50 mM phosphate buffer pH 7 or 8, respectively, to a final concentration of 20 mg dried cell weight/mL. Following, isopropanol (*i*-PrOH) to a final concentration of 10 % (v/v), NAD^+ or $NADP^+$ respectively, to a final concentration of 1 mM, and substrate to a final concentration of 50 mM, were added. Reactions were incubated for 20 h, at 30 °C

and 600 rpm, in the thermomixer. For coupling to the chemocatalytic step, the optimized conditions contained 4-5% MeCN instead of 0.4 % (v/v) DMSO.

6. One-pot synthesis of amines and alcohols from pyrrolidine or azepane

6.1. Preparative scale photoenzymatic synthesis of the amines 10a and 10b

A 22 mL Wheaton sample vial with a pressure release cap was charged with the amine substrate, pyrrolidine or azepane (1.42 mmol, 1 equiv), to achieve a final concentration of 400 mM in a MeCN/water (3:10) mixture. While the reaction mixture was kept on ice, H_2SO_4 (1.5 equiv) was added dropwise, causing a slight temperature rise. The vial was then charged with NaDT (32.4 mg, 1 mol%) and aqueous H_2O_2 (30% solution, 2.5 equiv), equipped with a stir bar, and sealed. The reaction mixture was irradiated at 365 nm until it turned an intense clear blue, signaling catalyst deactivation and the end of the reaction. Completion was achieved within 5-6 h.
As the next step, the vial was removed from the lamp and kept on ice while the crude reaction mixture was neutralized and basified (to pH 9) with NaOH (10 M stock, 0.7 equiv). Liquid Boc-anhydride (1.1 equiv) was then added to the mixture. The vial was sealed, covered with aluminum foil, and placed on a magnetic stirrer for 2 h at room temperature (RT) and 600 rpm.

For the biocatalytic conversion, the crude reaction mixture was diluted 3-fold with 50 mM HEPES buffer (pH 8). A sample (50-100 µL) was taken for GC-MS analysis. For coupling with the transaminases, lyophilized cells containing the ATA of interest were resuspended in the biocatalytic cocktail consisting of 2 M IPA and 2 mM PLP in 50 mM HEPES buffer, resulting in a final 6-fold dilution of the crude *N*-Boc-ketone. The magnetic stirrer bar was then removed, and the cell suspension was added slowly to the diluted crude reaction mixture while stirring. The final reaction setup contained approximately 5% MeCN, 1 M IPA, 1 mM PLP and 15-20 mg/mL of cells (Fig. S3). The vial was sealed, wrapped in aluminum foil, and incubated for 20 h at 30 °C and 600 rpm in an incubator shaker.

6.2. Preparative scale photoenzymatic synthesis of the alcohols 11a and 11b

The photooxyfunctionalization and *N*-protection of the amine substrate were performed similarly to the workflow described in Section 6.1. For the coupling in the same vial, the crude reaction mixture was diluted 3-fold for *Ls*KRED with 50 mM sodium phosphate buffer, pH 7, which contained 10% *i*-PrOH, or 3.8-fold for *Fs*KRED with 50 mM sodium phosphate buffer, pH 8, which contained 10% *i*-PrOH. Upon dilution, a sample (50-100 µL) was taken for GC-MS analysis. For coupling to the ketoreductases, lyophilized cells containing the KRED of interest were resuspended in the biocatalytic reaction mix, which consisted of 10% *i*-PrOH, 1 mM NAD+ (for *Ls*KRED) or NADP+ (for *Fs*KRED) in 50 mM sodium phosphate buffer (pH 7 or 8, respectively), resulting in a final 6-or 7.6-fold dilution of the crude *N*-Boc-ketone. The magnetic stirrer bar was then removed, and the cell suspension was added slowly to the diluted crude reaction mixture while stirring. The final reaction setup contained approximately 10% *i*-PrOH, 5% or 4% MeCN, 0.5 mM $NAD(P)^+$ and 15-20 mg/mL of cells (Fig. S3). The vial was sealed, wrapped in aluminum foil, and incubated for 20 h at 30 °C and 600 rpm in an incubator shaker.

For the lyophilized enzyme KRED-NADH-101, the same procedure was followed, but in this case the enzyme was resuspended in a buffer prepared from the Codexis dry KRED Recycle Mix N. This buffer contained 2 mM NAD^+, 3.2 mM $MgSO_4$, 152 mM D-glucose, 8.2 units/mL GDH, and 9% DMSO in 500 mM NaPi, pH 7.0. The crude chemical product was diluted 3-fold with 250 mM NaPi, pH 7.0. Upon addition of the enzyme suspension, the final reaction setup contained approximately 5% MeCN, 4.5% DMSO, 1 mM NAD^+, 1.6 mM $MgSO_4$, 76 mM D-glucose, 4.1 units/ mL GDH and 15-20 mg/mL enzyme.

6.3. Photochemoenzymatic reaction work-up

6.3.1. Amine products

Upon centrifugation (4,000 *g*, 10 min, RT) and separation from cell pellet, the crude chemoenzymatic reaction was extracted thrice with equal volume of hexane. The pooled organic phases were discarded, and the aqueous phase was basified to pH 12-14 using 10 M NaOH, while keeping the vessel on ice. Next, the basified aqueous phase was extracted thrice with equal volume of ethyl acetate, and the pooled organic phases were further washed twice with equal volume of brine. The resulting organic phase was additionally dried with anhydrous $MgSO_4$, followed by filtering through a 0.22 um Whatman filter, and solvent removal *in vacuo.* Purification of the crude product was performed by silica flash column chromatography. Fractions containing the product of interest were identified by TLC analysis and pooled. After concentration under reduced pressure, isolated yield was determined by weight, and product identity by NMR-spectroscopy.

6.3.2. Alcohol products

Upon centrifugation (4,000 *g*, 10 min, RT) and separation from cell pellet, the crude chemoenzymatic reaction was extracted thrice with equal volumes of ethyl acetate, and the pooled organic phases were further washed twice with equal volumes of brine. The resulting organic phase was additionally dried with anhydrous $MgSO_4$, followed by filtering through a 0.22 um Whatman filter, and solvent removal *in vacuo.* Purification of the crude product was performed by silica flash column chromatography. Fractions containing the product of interest were identified by TLC analysis and pooled. After concentration under reduced pressure, isolated yield was determined by weight and product identity by NMR-spectroscopy.

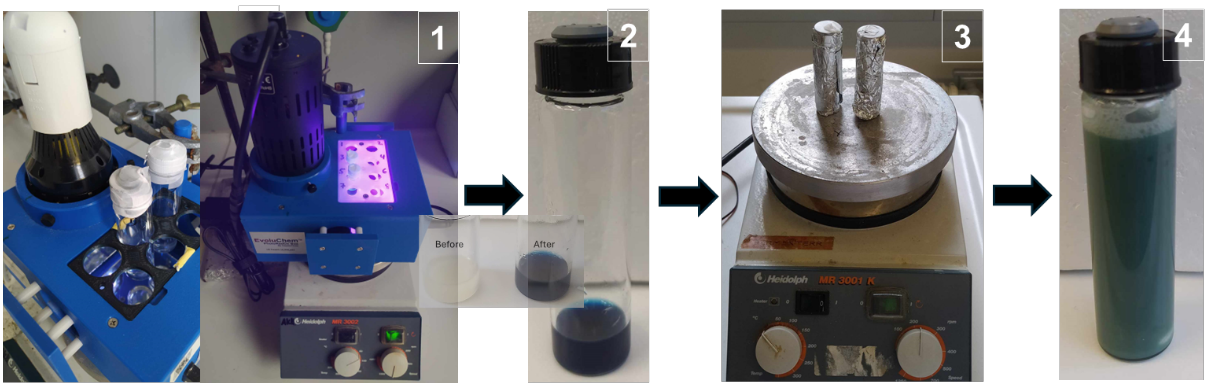

Figure S3. Photocatalytic setup and photoenzymatic synthesis workflow applied in this study, indicatively shown for the synthesis of (*S*)-**10a**.**1**. Hepatochem photoredox box device, with incorporated 365 nm-LED lamp, 6- or 8-position sample holder and fan system. The device is placed on a magnetic stirrer. **2**. Crude reaction product in a 22-mL clear glass vial, after decatungstate- based photo-oxyfunctionalization of aliphatic amine substrate (high scale setup as described in 4.2.2., 6.1 and 6.2.). The blue color indicates photocatalyst inactivation and the end of the reaction. **3**. *N*-protection of intermediate oxo-product, upon basification and addition of Boc_2O, stirring for 2 h (as described in 4.2.2., 6.1. and 6.2.). **4**. Whole-cell biocatalytic reaction mixture, upon dilution of the crude *N*-protected substrate with addition of biocatalyst and the rest of reagents (as described in 6.1. and 6.2.).

7. Analytical methods

7.1.1. Gas chromatography

To a 50 µL-sample of the crude protected aminoketone or crude amine, 5 µL of NaOH 10 M were added to ensure quantitative extraction with an organic solvent. Reactions containing the crude alcohol products were sampled (50 µL), without further basification. Afterwards, the samples were extracted with a 2-fold volume of ethyl acetate containing 1 mM dodecane as internal standard. The samples were vortexed for 5 s and reversed 6-8 times. Phases were separated by centrifugation (13,000 *g*, RT, 5 min). The organic phase was transferred into a fresh 1.5 mL tube, where it was dried upon addition of a pinch of anhydrous magnesium sulfate. The tube was briefly vortexed and left to stand for 5 min, before pelleting the magnesium sulfate by centrifugation (13,000 *g*, RT, 15 min). The dried organic phase (50 µL sampled) was diluted 1:10 with the extraction solvent, vortexed thoroughly and 100 µL were transferred into a GC vial. Conversions and substrate consumption were determined by GC-MS or GC-FID. Verification of intermediates and products was performed with the use of commercial standards and supplementary identification via GC-MS, where needed. Enantiomeric excess determination was performed via chiral GC-MS for **10a** and **11a**, via chiral GC-FID for **11b,** and via chiral HPLC-UV for **10b**.

Table S2. Method 1| GS-MS method for the determination of 9a **and** 10a **concentrations and** 10a **enantiomeric excess.**

**Method parameters**		
**Column**	HYDRODEX BETA-TBDAc, (25 m x 0,25 mm, film thickness: 0.25 µm)	Macheray-Nagel
**Injector**	T (°C)<br>V (µL)<br>Split ratio	220<br>1<br>1/10
**Carrier gas**	Helium<br>Linear velocity (cm/sec)	 37.1
**MS**	Ion source T (°C)<br>Interface T (°C)	220<br>220
**Temperature program**		
**Rate (°C/min)**	**T (°C)**	**Hold time (min)**
-	140	8.00 (2.1 min: solvent cut)
5.00	150	10.00
20.00	220	1.50

Table S3. Method 2| GS-MS method for the determination of 9a and 11a concentrations and 11a enantiomeric excess.

**Method parameters**		
**Column**	**HYDRODEX BETA-TBDAc, (25 m x 0,25 mm, film thickness: 0.25 µm)**	**Macheray-Nagel**
**Injector**	T (°C)<br>V (µL)<br>Split ratio	220<br>1<br>1/10
**Carrier gas**	Helium<br>Linear velocity (cm/sec)	 37.1
**MS**	Ion source T (°C)<br>Interface T (°C)	220<br>220
**Temperature program**		
**Rate (°C/min)**	**T (°C)**	**Hold time (min)**
-	130	8 (2.0 min: solvent cut)
5	150	10
20	220	3.5

Table S4. Method 3| GS-MS method for determination of 8c and 9c concentrations.

**Method parameters**		
**Column**	HYDRODEX BETA-TBDAc, (25 m x 0,25 mm, film thickness: 0.25 µm)	Macheray-Nagel
**Injector**	T (°C)<br>V (µL)<br>Split ratio	220<br>1<br>1/10
**Carrier gas**	Helium<br>Linear velocity (cm/sec)	 37.1
**MS**	Ion source T (°C)<br>Interface T (°C)	220<br>220
**Temperature program**		
**Rate (°C/min)**	**T (°C)**	**Hold time (min)**
-	80	8.00 (3 min: solvent cut)
10	150	10.00
20	220	3.50

Table S5. Method 4| Non-chiral GS-MS method for the quantification of 9b **and** 10b.

**Method parameters**		
**Column**	HYDRODEX BETA-TBDAc, (25 m x 0,25 mm, film thickness: 0.25 µm)	Macheray-Nagel
**Injector**	T (°C)<br>V (µL)<br>Split ratio	220<br>1<br>10
**Carrier gas**	Helium<br>Linear velocity (cm/sec)	 37.1
**MS**	Ion source T (°C)<br>Interface T (°C)	220<br>220
**Temperature program**		
**T (°C)**	**Rate (°C/min)**	**Hold time (min)**
-	130	8 (2 min: solvent cut)
5	140	10
5	170	10
20	220	6.5

Table S6. Method 5| Non-chiral GS-MS method for the quantification of 9b **and** 11b.

**Method parameters**		
**Column**	HYDRODEX BETA-TBDAc, (25 m x 0,25 mm, film thickness: 0.25 µm)	Macheray-Nagel
**Injector**	T (°C)<br>V (µL)<br>Split ratio	220<br>1<br>10
**Carrier gas**	Helium<br>Linear velocity (cm/sec)	 37.1
**MS**	Ion source T (°C)<br>Interface T (°C)	220<br>220
**Temperature program**		
**T (°C)**	**Rate (°C/min)**	**Hold time (min)**
-	130	8 (2 min: solvent cut)
5	150	10
20	220	3.5

Table S7. Method 6| GC-FID method for determination of 9b and 11b concentrations, and 11b enantiomeric excess.

**Method parameters**		
**Column**	CP Chirasil Dex CB (25.0 m x 0.25 mm, 0.25 µm)	Agilent
**Injector**	T (°C)<br>V (µL)<br>Split ratio	220<br>1<br>17.4
**Carrier gas**	Hydrogen<br>Linear velocity (cm/sec)	 36.3
**FID**	T (°C)	220
**Temperature program**		
**Rate (°C/min)**	**T (°C)**	**Hold time (min)**
-	140	5
5	150	10
10	220	4

7.1.2. GC-based calibrations

Calibration curves were prepared for quantification of the *N*-Boc-azacyclic ketones, amines and alcohols, which were synthesized in this study. Quantification was performed via GC-MS or GC-FID, according to the above-described chromatographic methods (7.1).
For each set of conditions, calibrations were performed with normalization against dodecane, used as the internal standard. The response factor for dodecane was determined under the same experimental conditions, and all measured concentrations were calculated relative to its integrated signal. The calibration curves were prepared in three independent dilution series of the respective analyte.

7.2. HPLC

Chiral HPLC analytics was used for chiral separation of *N*-Boc-4-aminoazepanes (**10b**). The crude reaction amine product was sampled, basified, and extracted, in this case with ethyl acetate, as mentioned above. In a brown 1.5-mL Eppendorf tube, 2 µmol **10b** (as extract) were mixed with 40 µmol $NaHCO_3$ (from a 100 mM stock solution) and 2.8 µmol *N*-α-(2,4-dinitro-5-fluorophenyl)-L -alaninamide – Marfey's reagent – (from a 50 mM stock solution in 1% (v/v) acetone) [33]. The mixture was briefly vortexed and then placed for 1 h on the thermomixer at 37 °C and 600 rpm. Finally, the mixture was diluted to 2.5 mM with the mobile phase (0.1% (v/v) aq. TFA: MeCN at a 65:35 ratio), before 100 µL were transferred to a glass vial for HPLC analysis.

Table S8. Method 7| HPLC-UV method for the determination of 10b **enantiomeric excess**

**Method parameters**		
**Column**	LiChrospher® 100 RP-18e (25 cm × 4 mm, particle size: 5 µm)	Merck
**Injector**	T (°C)<br>V (µL)	25 °C<br>5
**Column oven**	T (°C)	25 °C
**UV**	345 nm	
**Mobile phase gradient program**		
**MeCN**	**0.1% (v/v) aq.TFA**	**Flow rate (mL/min)**
35	65	1
70	30	1
35	65	1
35	65	1

7.3. Thin layer chromatography

To assess product formation, as well as product isolation during column purification, thin layer chromatography (TLC) was employed. For **9a** detection from crude **9a** product samples, the TLC was developed with hexane/EtOAC 1:1. For visualization, the plate was stained with the permangantate stain[34] and following a short heating step with the heat gun, **9a** became visible (as an orange spot on a yellowish background). For **9a** purification, a 0-30% EtOAC gradient in hexane was employed, following the same procedure for TLC development and visualization. For **10a** detection from crude **10a** product samples, the TLC plate was developed with DCM containing 0.5% NH_3 and 3 % (v/v) MeOH. Afterwards, the TLC plate was stained with the ninhydrin stain[35] and following a short heating step, the amine product became visible (as an orange/pink spot on a lightly pink background). For **10a** purification, a 2-3% MeOH gradient in DCM was employed, following the same procedure for TLC development and visualization. For **9a** and **17a** detection from crude **17a** product samples, the TLC plate was developed with hexane/EtOAC 1:2. After elution, the TLC plate was stained with the *p*-anisaldehyde stain[35] and following a short heating step, the respective products became visible (as yellow spot for **9a** and white spot for **17a** on a lightly pink background). For **17a** purification, a hexane/EtOAC mobile phase 1:1 was employed, following the same procedure for TLC development and visualization.

8. Product characterization

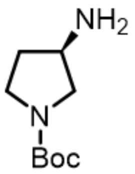

(*R*)-*N*-Boc-3-aminopyrrolidine, (*R*)-10a: Synthesized according to general procedure described in 6.1. 1.42 mmol **7a**, 0.0142 mmol NaDT and 300-320 mg ATA-117-Rd11 dry cells, afforded 91% (GC-yield) with > 98% ee. The product was isolated via flash column purification, as colorless/light yellow oil (80 mg, 40% yield). ^{1}H NMR (300 MHz, CDCl3) δ 3.60 – 3.32 (m, 4H), 3.02 (dd, J = 16.7, 8.8 Hz, 1H), 2.03 (ddt, J = 12.5, 7.8, 6.1 Hz, 1H), 1.67 – 1.56 (m, 1H).$^{13}C\{^{1}H\}$ NMR (101 MHz, CDCl3) δ 154.75, 79.26, 54.53, 54.18, 51.48, 50.65, 44.44, 44.07, 34.78, 34.33, 28.59, 28.48. The NMR-spectra are in agreement with literature [35]

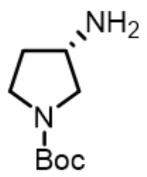

(*S*)-*N*-Boc-3-aminopyrrolidine, (*S*)-10a: Synthesized according to general procedure described in 6.1. 1.42 mmol **7a**, 0.0142 mmol NaDT and 300-320 mg 3HMU dry cells, afforded 90% (GC-yield) with > 99% ee. The product was isolated via flash column purification, as colorless/light yellow oil (88 mg, 45% yield). ^{1}H NMR (300 MHz, CDCl3) δ 3.57 – 3.23 (m, 4H), 3.06 – 2.91 (m, 1H), 2.08 – 1.91 (m, 1H), 1.62 (q, J = 6.0 Hz, 1H), 1.42 (s, 9H).$^{13}C\{^{1}H\}$ NMR (75 MHz, CDCl3) δ 154.75, 79.23, 54.58, 54.23, 51.51, 50.68, 44.45, 44.08, 34.84, 34.39, 28.60. The NMR-spectra are in agreement with literature [35]

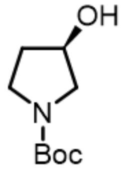

(*R*)-*N*-Boc-3-hydroxypyrrolidine, (*R*)-**11a:** Synthesized according to general procedure described in 6.1 1.42 mmol **7a**, 0.0142 mmol NaDT and 300-320 mg *Ls*KRED dry cells, afforded 85 (GC-yield) with > 95 ee.. The product was isolated via flash column purification, as white/ light yellow powder (80 mg, 40% yield). ^{1}H NMR (300 MHz, CDCl3) δ 3.60 – 3.32 (m, 4H), 3.02 (dd, J = 16.7, 8.8 Hz, 1H), 2.03 (ddt, J = 12.5, 7.8, 6.1 Hz, 1H), 1.67 – 1.56 (m, 1H). $^{13}C\{^{1}H\}$ NMR (101 MHz, CDCl3) δ 154.75, 79.26, 54.53, 54.18, 51.48, 50.65, 44.44, 44.07, 34.78, 34.33, 28.59, 28.48. The NMR-spectra are in agreement with literature. [22]

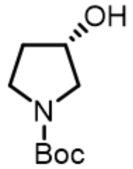

(*S*)-*N*-Boc-3-hydroxypyrrolidine, (*S*)-**11a:** Synthesized according to general procedure described in 6.1 1.42 mmol **7a**, 0.0142 mmol NaDT and 310-320 mg *Fs*KRED dry cells, afforded 70% (GC-yield) with > 99% ee. The product was isolated via flash column purification, as white/ light yellow powder (79 mg, 40% yield). ^{1}H NMR (400 MHz, DMSO) δ 4.88 (d, J = 3.4 Hz, 1H), 4.21 (tq, J = 7.4, 3.2 Hz, 1H), 3.26 (h, J = 4.5 Hz, 3H), 3.10 (dp, J = 11.3, 1.8 Hz, 1H), 1.83 (dhept, J = 17.7, 4.6 Hz, 1H), 1.76 – 1.66 (m, 1H), 1.39 (s, 9H). $^{13}C\{^{1}H\}$ (101 MHz, DMSO) δ 153.69, 78.03, 69.30, 68.46, 54.09, 53.85, 43.78, 43.55, 33.66, 32.90, 28.18. The NMR-spectra are in agreement with the literature.[22]

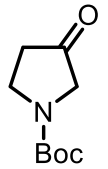

***N*-Boc-3-pyrrolidinone, (9a):** Synthesized according to general procedure described in Schultz et al.[32], 1 mmol **7a**, 0.010 mmol NaDT, afforded 92% (GC yield). The product was isolated via flash column purification, as light-yellow oil (78 mg, 42% yield). ^{1}H NMR (400 MHz, CDCl3) δ 3.80 – 3.71 (m, 4H), 2.57 (t, J = 7.8 Hz, 2H), 1.47 (s, 9H). The NMR-spectra are in agreement with literature [32]

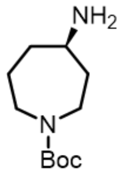

(*R*)-*N*-Boc-4-aminoazepane, (*R*)-**10b:** Synthesized according to general procedure described in 6.1. 1.42 mmol **7b**, 0.0142 mmol NaDT and 310-320 mg 3HMU dry cells, afforded 70% (GC-yield) with >99 % ee.

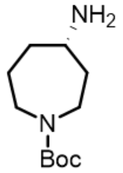

(*S*)-*N*-Boc-4-aminoazepane, (*S*)-**10b:** Synthesized according to general procedure described in 6.1. 1.42 mmol **7b**, 0.0142 mmol NaDT and 310-320 mg ATA-117 dry cells, afforded 51% (GC-yield), with 95 % ee.

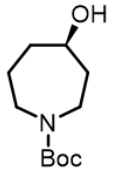

(*R*)-*N*-Boc-4-hydroxyazepane, (*R*)-**11b:** Synthesized according to general procedure described in 6.1. 1.42 mmol **7b**, 0.0142 mmol NaDT and 320-350 mg KRED-NADH-101, afforded 80%, GC-yield (average of triplicate runs) with 98 % ee.

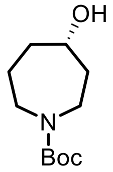

(*S*)-*N*-Boc-4-aminoazepane, (*S*)-**11b:** Synthesized according to general procedure described in 6.1. 1.42 mmol **7b**, 0.0142 mmol NaDT and 310-320 mg *Ls*KRED dry cells, afforded 82% (GC-yield) with > 98 ee.

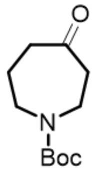

***N*-Boc-hexahydro-1H-azepin-4-one, (9b):** Synthesized according to general procedure described in 6.1. 1.42 mmol **7b**, 0.0142 mmol NaDT, afforded 94% (GC-yield, average of independent triplicates).

[a] GC yields are referring to average of independent triplicates. SD was < 10%.

9. Study of reaction conditions for photo-oxyfunctionalization and *N*-protection, before coupling to biocatalysis.

Table S9. Adaptation of the solvent conditions for the photochemical oxyfunctionalization and *N*-protection step before coupling to biocatalysis

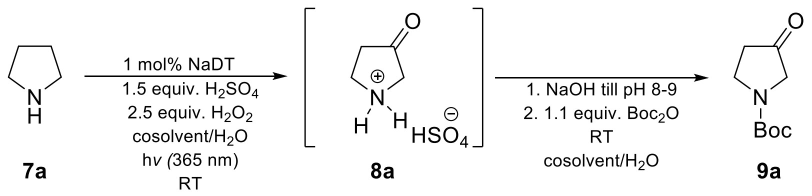

A	NADT-mediated pyrrolidine oxyfunctionalization		
**Entry**	**Organic solvent content**	**Conversion (%) [a]**	**Conversion (%) [b]**
1[c]	50% MeCN (0.7 M)	N/A	64 (refers to 0.7 M)
2[d]	50% MECN (0.7 M)	N/A	82 (refers to 0.7 M)
3	50% MeCN	95.1	89.0
3	40 % MeCN	95.2	92.2
4	30 % MeCN	94.0	92.1
5	20 % MeCN	90.2	65.5
6	50% DMSO	ND	N/A
7	50% MeOH	21.7	N/A
8	50% Acetone	ND	N/A
9	50% Isopropyl alcohol	ND	N/A
10	50% *t*-BuOH	80.1	N/A
**B**	***N*-Boc protection**		
**Entry**	**Organic solvent content**	**Conversion (%) [a]**	**Conversion (%) [b]**
1	50 % MeCN	99.5	99.1
2	40 % MeCN	99.8	99.5
3	30 % MeCN	99.9	99.4
4	20 % MeCN	97.5	90.5
5	10% MeCN	96.5	N/A
6	5% MeCN	95.1	N/A
7	50% DMSO	ND	N/A
8	50% MeOH	94.1	N/A
9	50% Acetone	65.6	N/A
10	50% Isopropyl alcohol	75.2	N/A
11	50% *t*-BuOH	95.2	N/A

[a] 100 mM substrate, [b] 400 mM substrate, [c] 700 mM substrate-result produced by Schultz et al.[32], [d] 700 mM substrate-result produced in this study | **A**. The effect of solvent conditions on oxyfunctionalization efficiency was studied on the substrate pyrrolidine (**7a**) and the evaluation was based on the quantification of the protected product, *N*-Boc-3-pyrrolidinone (**9a**). **B**. The effect of solvent conditions on the *N*-protection efficiency was studied on the substrate 2-pyrrolidinone (**7c**) and evaluation was based on the quantification of the *N*-Boc-2-pyrrolidinone (**9c**). Conversions (%) post 2h (100 mM substrate) and

post 3h (400 mM substrate) are shown. N/A: experiment not conducted, ND: desired product not detected. For all mentioned conditions, 0.5 mL-reactions in 1.5 mL clear glass vials, were conducted. The photoredox device was used as light source. Average values of independent triplicate GC-MS measurements are stated (standard deviation < 10%).

10. Biocatalyst activity screening

Table S10. Transaminase specific activities towards 9a **and** 9b

**Entry**	**TA**	**Specific activity (U/mg) ± SD** [a]		
		**9a**	**9b**	**Pyruvate**
1	3HMU	0.50±0.03	0.02±0.00	1.09±0.01
	*Vflu*TA	0.02±0.00	N/A	1.24±0.02
3	*At*TA	N/A	N/A	N/A
4	ATA-117	N/A	N/A	N/A
	ATA-117-Rd11	N/A	0.10±0.04	N/A
5	TA-01	ND	N/A	N/A
6	TA-05	ND	N/A	N/A
7	TA-10	0.36±0.01	N/A	1.01±0.02
8	CviTA	1.97±0.37	0.08±0.01	1.14±10
9	*Cvi*TA_M1	0.56±0.02	ND	1.14±0.03
10	*Cvi*TA_M2	ND	N/A	N/A
11	*At*TA_M1	0.02±0.01	N/A	0.70±0.02
12	*At*TA_M2	N/A	0.01±0.00	N/A
14	CD5TA	N/A	ND	N/A
14	*CD5*TA_M1	0.42±0.05	ND	0.42±0.05
15	TA-v2	0.02±0.01	N/A	0.27±0.00

[a] Specific activities and standard deviations (SD) for **9a** and pyruvate were determined based on the acetophenone assay, as described in 3.1. All measurements were performed in triplicates. Only pyruvate-accepting TAs are included here. N/A: experiment not conducted, ND: activity not detected.

Table S11. Transaminase-catalyzed conversions of 9a **and** 9b[a]

		9a		9b	
**Entry**	**TA**	**(%) Conversion[a] ± SD**	**ee**	**(%) Conversion[a] ± SD**	**ee**
1	TA-10	15±0.3	>99 (S)	N/A	N/A
2	CviTA	77±10	>99 (S)	64±10	>97 (R)
3	3HMU	63±1.0	>99 (S)	86±1	>99 (R)
4	ATA-117	N/A	N/A	35±2	>95 (S)
5	ATA-117-Rd11	90±1.0	99 (R)	19±1	50
6	AtTA_M2	7±0.02	>99 (R)	N/A	N/A
7	CD5TA	36±0.2	>99 (S)	N/A	N/A
8	CD5TA_M1	71±3.0	>99 (S)	N/A	N/A

[a] **9a** and **9b** conversions post 20h and their standard deviations (SD), as determined via GC-MS, are shown. Reactions consisted of 0.1 mg/mL enzyme, added as crude cell extract, as well as 1 M IPA, 1 mM PLP, 20 mM *N*-Boc-3-pyrrolidinone, and 2% (v/v) DMSO, in 50 mM HEPES (=pH 8.0), and were carried out as described in 5.1.1. All measurements were performed in triplicates. Only IPA-accepting TAs are included here. N/A: experiment not conducted.

Table S12. Activity comparison between different biocatalyst preparations [a]

Entry	Biocatalyst	Cell-free extracts	Resting cells	Lyophilized cells
1	3HMU	75±1.0	86±4.0	72±7.0
2	ATA-117-Rd11	75±2.0	87±3.0	81±10
3	*Fs*KRED	N/A	77±1.0	87±1.0
4	*Ls*KRED	N/A	80±2.0	87±4.0

[a] Conditions per setup are as described in 5.1 and 5.2. Conversions refer to starting substrate **9a** used at a concentration of 20 mM.

Table S13. Ketoreductase-catalyzed conversions of 9a **and** 9b

		9a		9b	
**Entry**	**KRED**	**(%) Conversion ± SD**	**ee**	**(%) Conversion ± SD**	**ee**
1	*Fs*KRED	75±5.0 [a]	>98 (*S*)	77±0.3 [b]	60 (R)
2	*Fp*KRED	30±3.0 [a]	83 (*S*)	12±0.4 [b]	65 (R)
3	*Lb*KRED	23±4.0 [a]	57 (*R*)	N/A	N/A
4	*Lk*KRED	12±0.3 [a]	>99 (*S*)	N/A	N/A
5	*Ls*KRED	87±4.0 [b]	>95 (R)	82±3.7 [b]	>98 (S)
6	KRED-NADH-101	N/A [c]	-	85±5.0 [c]	98 (R)

[a] Reactions consisted of 50% (v/v) resting cells of the respective overexpressed cell culture (final OD_{600}:10), 5% (v/v) glucose, 20 mM substrate and 0.4 % (v/v) DMSO in 50 mM sodium phosphate buffer pH 7.5, as described in 5.2.2.

[b] Reactions consisted of 15 mg/mL lyophilized cells of the respective *Ls*KRED overexpressing cell culture, 1 mM NAD or NADP, 10% *i*-PrOH, 50 mM substrate, 0.4 % (v/v) DMSO in 50 mM NaPi (= pH 7.5), as described in 5.2.3.

[c] Reactions consisted of 20 mg/mL lyophilized KRED-NADH-101, 1 mM NAD, 1.6 mM $MgSO_4$, 76 mM D-glucose, 4.1 units/ mL GDH, 50 mM substrate, 4.5 % (v/v) DMSO in 250 mM NaPi (= pH 7.0).

All reactions were carried out in 0.5 mL volumes at 30 °C. Reactions were extracted post 20h and analyzed via chiral GC-MS (as described in section 7.1). All measurements were performed in triplicates. N/A: experiment not conducted, ND: alcohol not detected.

Table S14. Compatibility of ATA and KRED towards components from the photocatalytic 9a **production**

Entry	Variation from standard condition [c]	Conversion (%)			
		3HMU	ATA-117-Rd11	*Fs*KRED	*Ls*KRED
1	None [a]	72±2	81±10	83±1	79±2
2	7 % DMSO	81±10	N/A	73±5	N/A
3	+ NaDT (0.1 mM) [b]	72±0.2	N/A	70±2	N/A
4	+ NaDT (1 mM) [b]	68±4	N/A	74±6	N/A
5	+Boc_2O (10 mM) [b]	78±1	N/A	70±5	N/A
6	+Boc_2O (100 mM) [b]	49±5	N/A	42±2	N/A
7	+NaDT (1 mM), Boc_2O (10 mM) [b]	71±1	N/A	75±2	N/A
8	2.5 % MeCN	62±2	95±4	N/A	N/A
9	5 % MeCN	70±1	N/A	83±3	76±1
10	7% MeCN	72±4	93±1	13±2	80±2
11	10% MeCN	49±2	96±0.4	N/A	72±2

[a] Standard ATA conditions: 0.1 mg/mL crude cell extract containing overexpressed 3HMU or ATA-117-Rd11, 1M IPA, 1 mM PLP, 20 mM **9a** and 2% (v/v) DMSO in 50 mM HEPES (=pH 8); standard KRED conditions: 50% (v/v) resting cells of *Fs*KRED or *Ls*KRED overexpressed cell culture (final OD600:10), 5% (v/v) glucose, 20 mM **9a** and 0.4% (v/v) DMSO, in 50 mM NaPi (= pH 7 (*Ls*KRED) or 8 (*Fs*KRED)). [b] Entries 3-7, refer to setups containing 7% (v/v) DMSO in addition to tested reagent. [c] In all shown cases, 0.5 mL-reactions were carried out at 30 °C. Reactions were extracted after 20 h, followed by GC-MS analysis. No effect on ee values from a variation of these conditions was observed. N/A: experiment not conducted.

Table S15. Comparison of ATA-biocatalytic conversions of crude 9a **and pure** 9a **under adapted conditions**

Entry	Biocatalyst	Conversion (%)	
		Crude 9a	Standard 9a
1	3HMU Crude cell extract	66±1.0	62±4.0
2	3HMU resting cells	63±1.0	62±2.0
3	ATA-117-Rd11 crude cell extract	67±0.3	68±2.0
4	ATA-117-Rd11 resting cells	74±2.0	60±3.0

Crude **9a** was diluted to 5 % (v/v) MeCN and was charged with crude cell extract or resting cells containing the overexpressed ATA of interest. In case of the pure standard **9a**, biocatalytic reactions were set up in presence of 5 % (v/v) MeCN. 0.5 mL-reactions were set up as described in the previous sections and were performed in triplicates. Final substrate concentration was 50 mM. reactions were extracted and analyzed via GC post-20 h.

Table S16. Biocatalytic conversions under adapted conditions[a] for chemobiocatalytic coupling

Entry	Biocatalyst	Conversion (%)
1	3HMU[b]	94±5.0
2	ATA-117-Rd11[b]	90±2.1
3	*Fs*KRED[b]	78±6.3
4	*Ls*KRED[b]	94±3.0
5	3HMU[c]	70±7.1
6	ATA-117[c]	58±5.0
7	*Ls*KRED[c]	86±4.0
8	KRED-NADH-107[c]	83±3.3

[a] Conditions refer to the optimized biocatalysis setup containing MeCN as cosolvent and employing lyophilized cells containing the overexpressed biocatalyst of interest or lyophilized enzyme in case of KRED-NADH-101, to a final concentration of 15-20 mg/ mL., as described in 5.1.3 and 5.2.3. [b]Conversions refer to starting **9a** substrate concentration of 50 mM, post 20 h. [c]Conversions refer to starting **9b** substrate concentration of 50 mM, post 20 h. All measurements were performed in triplicates.

11. Photoenzymatic setup evaluation

Table S17. Photooxyfunctionalization[a] of alternative substrates

Entry	Substrate	Desired product	Conversion (%)
1	**7a**	**9a**	90
2	1-butylamine	*tert*-butyl (3-oxobutyl) carbamate	92
3	3-phenyl-propanamine	*tert*-butyl (3-oxo-3-phenylpropyl) carbamate	85
4	azepane	**9b**	93
5	*N*-Bz-pyrrolidine	*N*-Bz-3-pyrrolidinone	ND
6	*N*-Cbz-pyrrolidine	*N*-Cbz-3-pyrrolidinone	ND
7	indane	1-indanone	68
8	1--phenylpentan-1-one	1-phenyl-1,4-pentandione	82
9	cyclohexene	cyclohexenone	5
10	cycloxehane	cyclohexanone	63
11	cyclopentane	cyclopentanone	ND

[a] Photo-oxyfunctionalization in 0.5 mL reaction setups and 100 mM substrate. The photo-oxyfunctionalization was performed as described in 4.2.1, but with 50% MeCN. Where needed, *N*-protection was performed upon basification of the crude photo-oxyfunctionalization product, and solvent-free addition of 1.1 equiv. Boc_2O. ND: desired product not detected.

12. Stereochemistry assignment via GC- and HPLC analysis

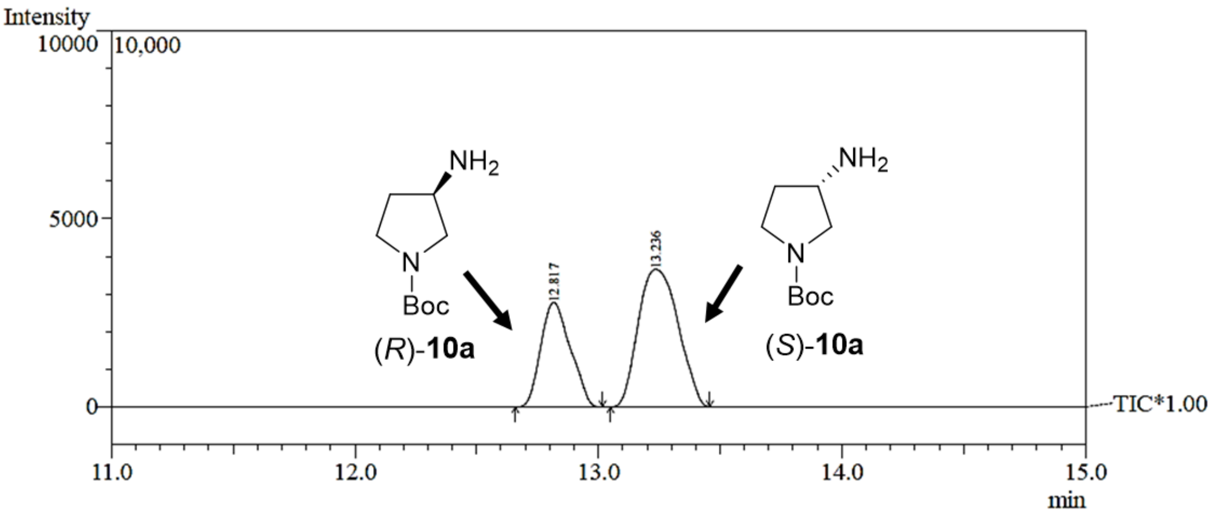

Analyte	RT[a] (min)	% Area
(*R*)-**10a**	12.817	35.9
(*S*)-**10a**	13.236	64.1

Figure S4. GC-chromatogram of mixture of standards (R)-**10a** and (S)-**10a** | Method 1. [a]RT: Retention time

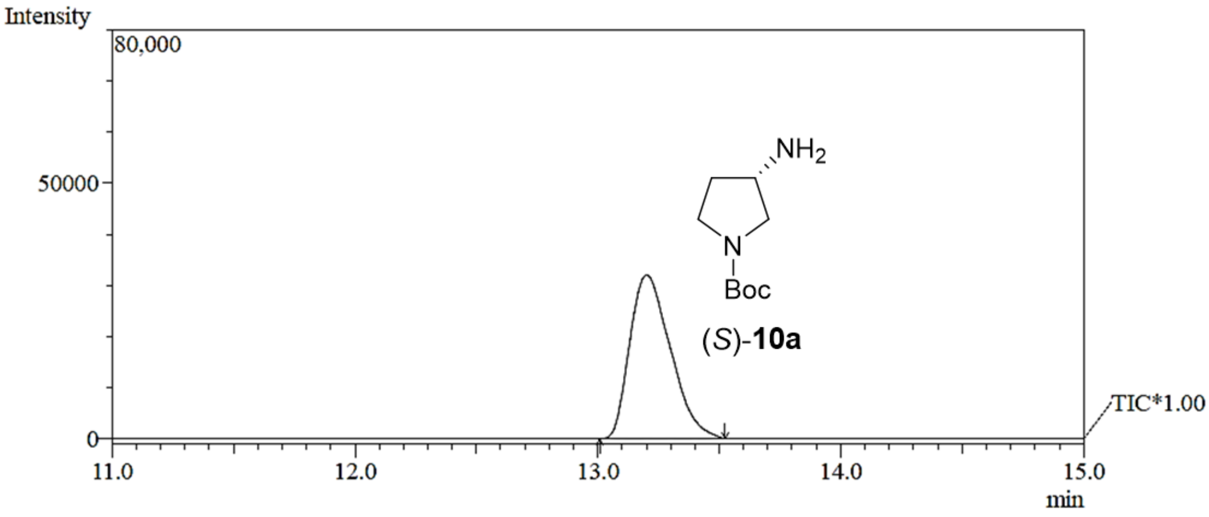

Analyte	RT (min)	% Area
(*S*)-**10a**	13.201	100

Figure S5. GC-chromatogram of standards (*S*)-**10a** | Method 1.

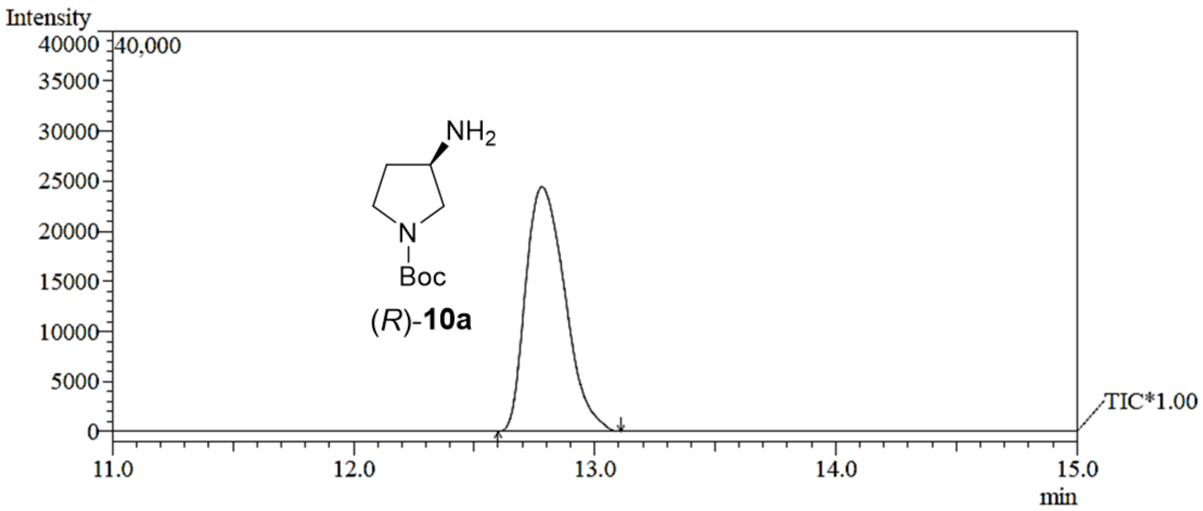

Analyte	RT (min)	% Area
(*R*)-**10a**	12.782	100

Figure S6. GC-chromatogram of standard (*R*)-**10a** | Method 1.

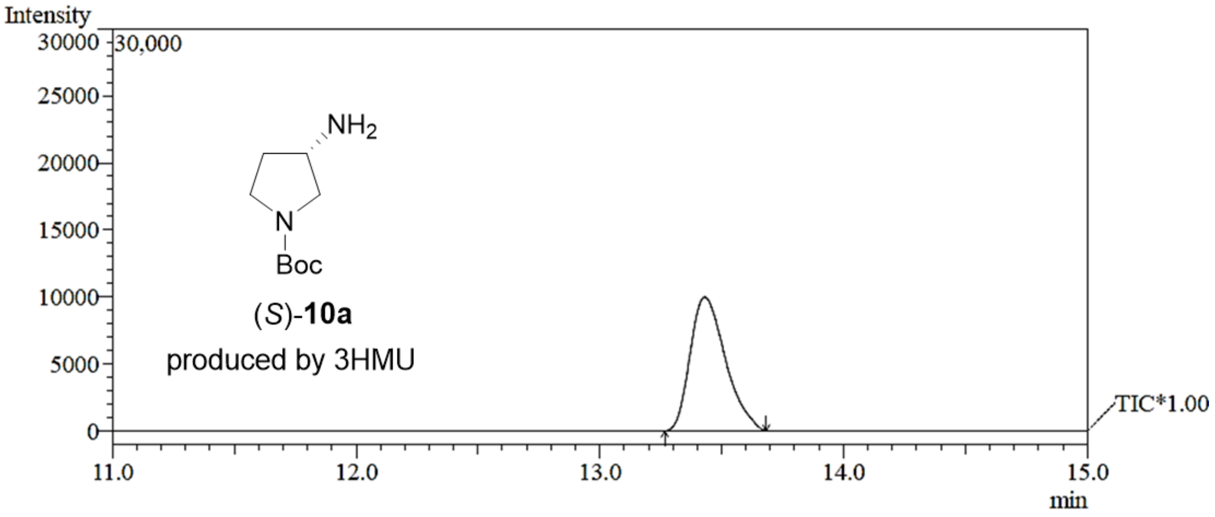

Analyte	RT (min)	% Area
(*S*)-**10a**	13.431	100

Figure S7. GC-chromatogram of enantioenriched **10a**, produced via 3HMU-mediated transamination | Method 1

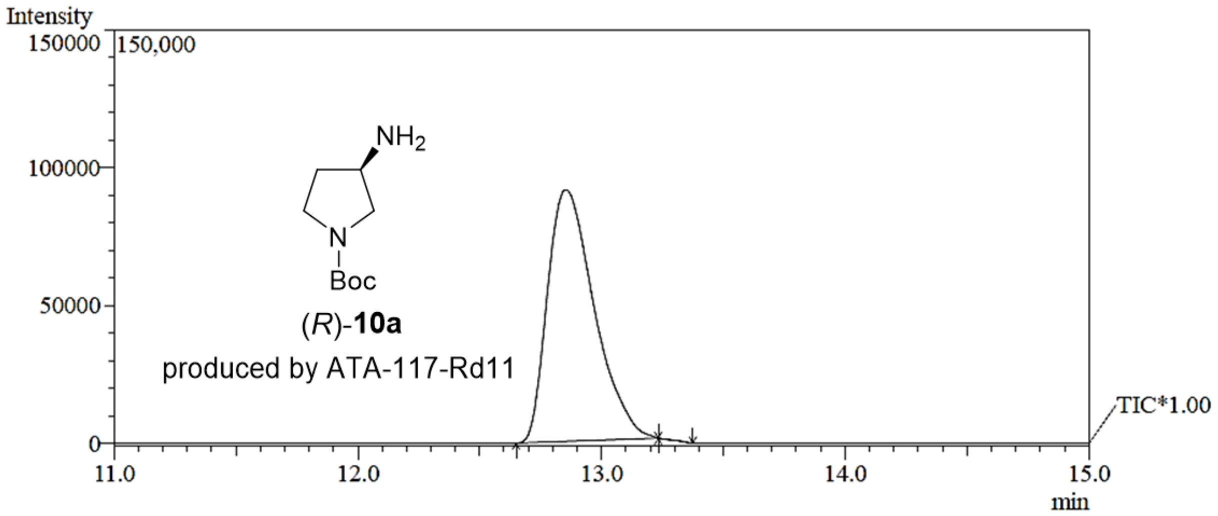

Analyte	RT (min)	% Area
(*R*)-**10a**	12.854	99.65
(*S*)-**10a**	13.285	0.35

Figure S8. GC-chromatogram of enantioenriched **10a**, produced via ATA-117-Rd11-mediated transamination | Method 1

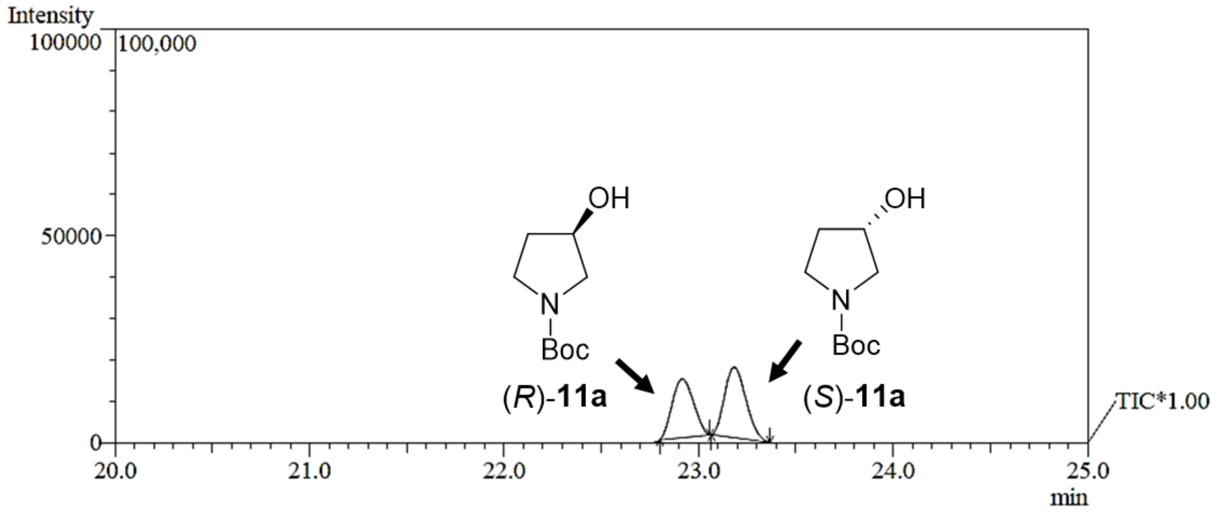

Analyte	RT (min)	% Area
(*R*)-**11a**	22.916	45.53
(*S*)-**11a**	23.182	54.47

Figure S9. GC-chromatogram of standard (*rac*)-**11a** | Method 2

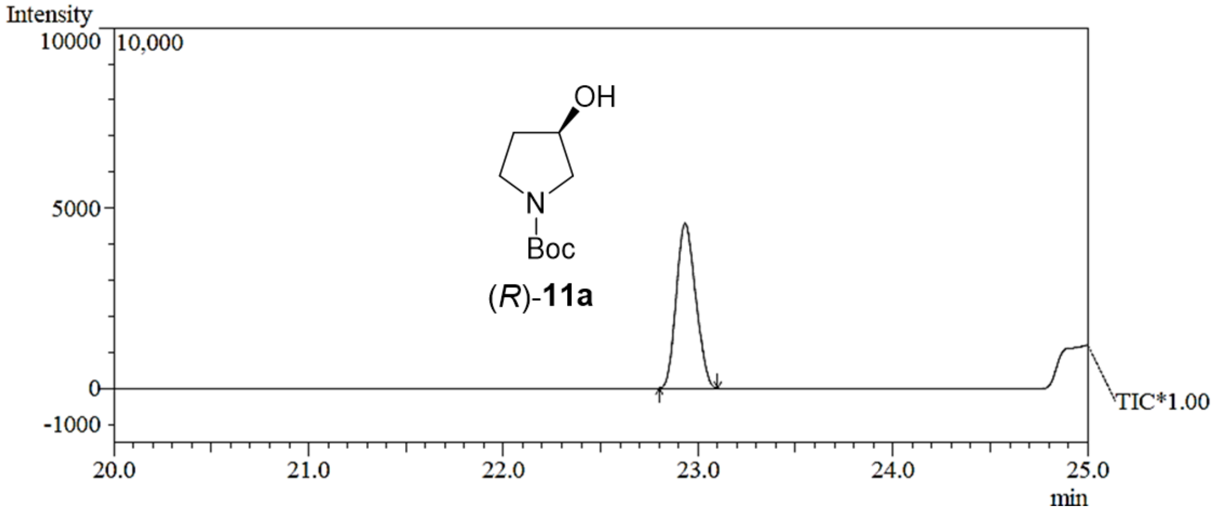

Analyte	RT (min)	% Area
(*R*)-**11a**	22.933	100

Figure S10. GC-chromatogram of standard (*R*)-**11a** | Method 2

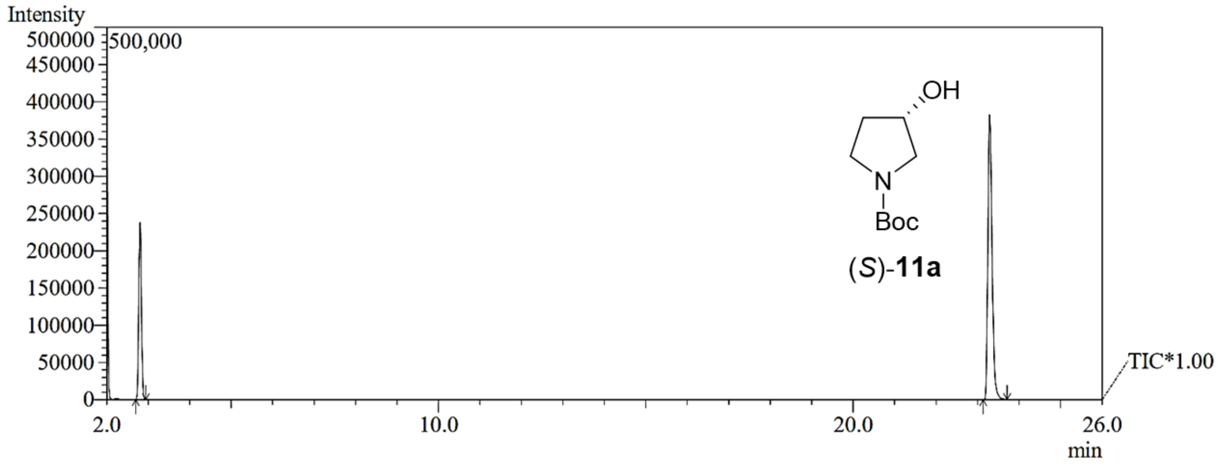

Analyte	RT (min)	% Area
**IS**[a]	2.880	39.18
(*S*)-**11a**	23.291	60.82

Figure S11. GC-chromatogram of standard (*S*)-**11a** | Method 2. [a]IS: Internal Standard; IS (dodecane) represents the internal standard used for calibration.

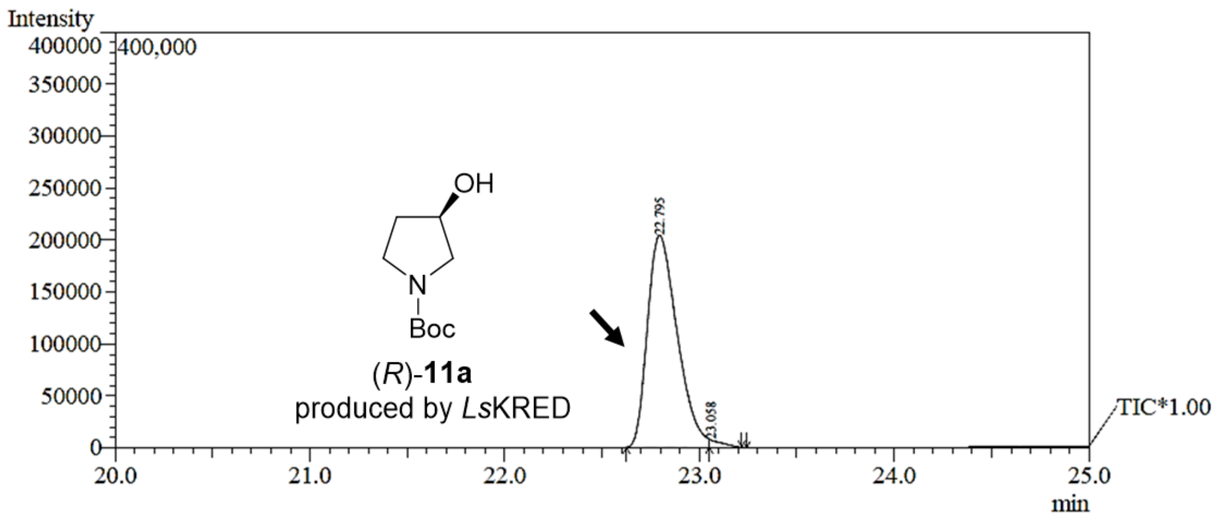

Analyte	RT (min)	% Area
(*R*)-**11a**	22.795	98.82
(*S*)-**11a**	23.058	1.18

Figure S12. GC-chromatogram of enantioenriched **11a**, produced via *Ls*KRED-mediated ketoreduction | Method 2

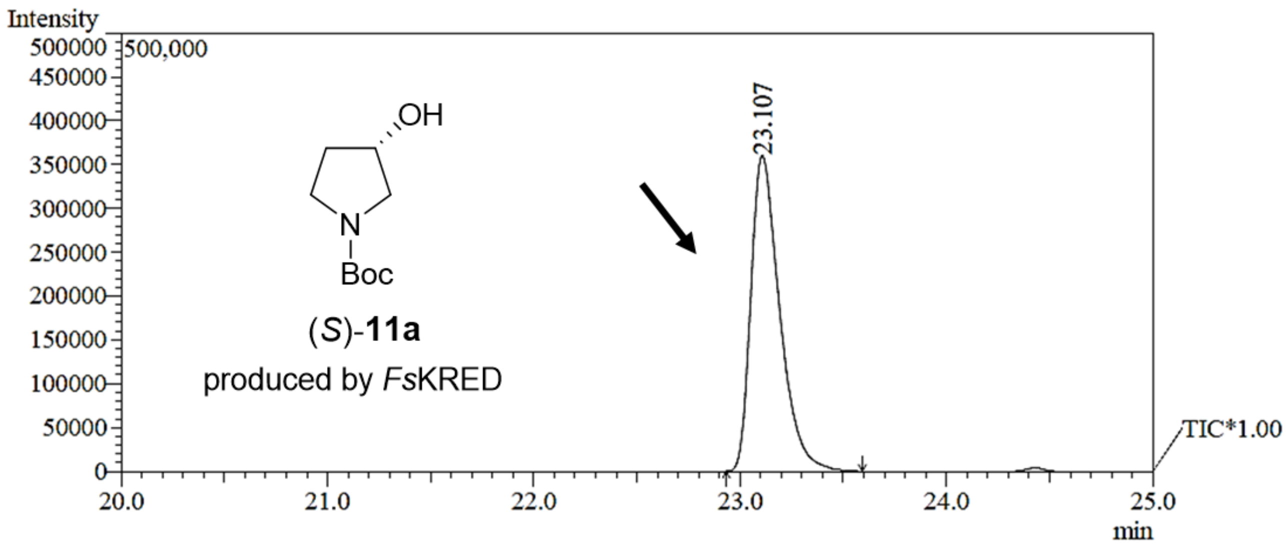

Analyte	RT (min)	% Area
(*S*)-**11a**	23.107	100

Figure S13. GC-chromatogram of enantioenriched **11a**, produced via *Fs*KRED-mediated ketoreduction | Method 2:

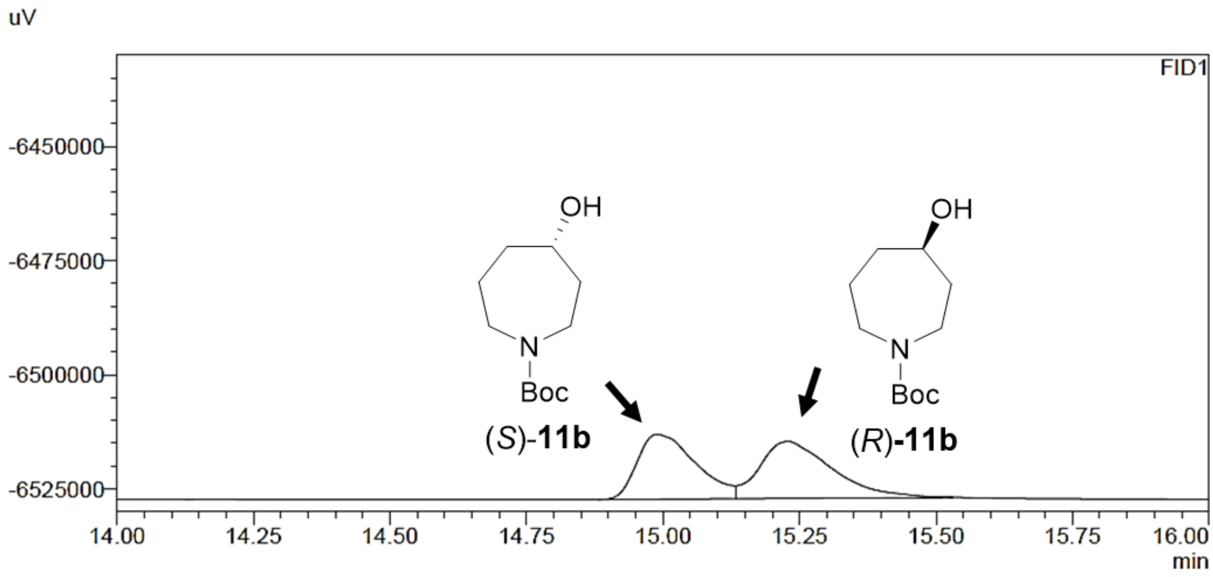

Analyte	RT (min)	% Area
(*S*)-**11b**	14.989	48.08
(*R*)-**11b**	15.229	51.92

Figure S14. GC-chromatogram of standard (*rac*)-**11b** | Method 7

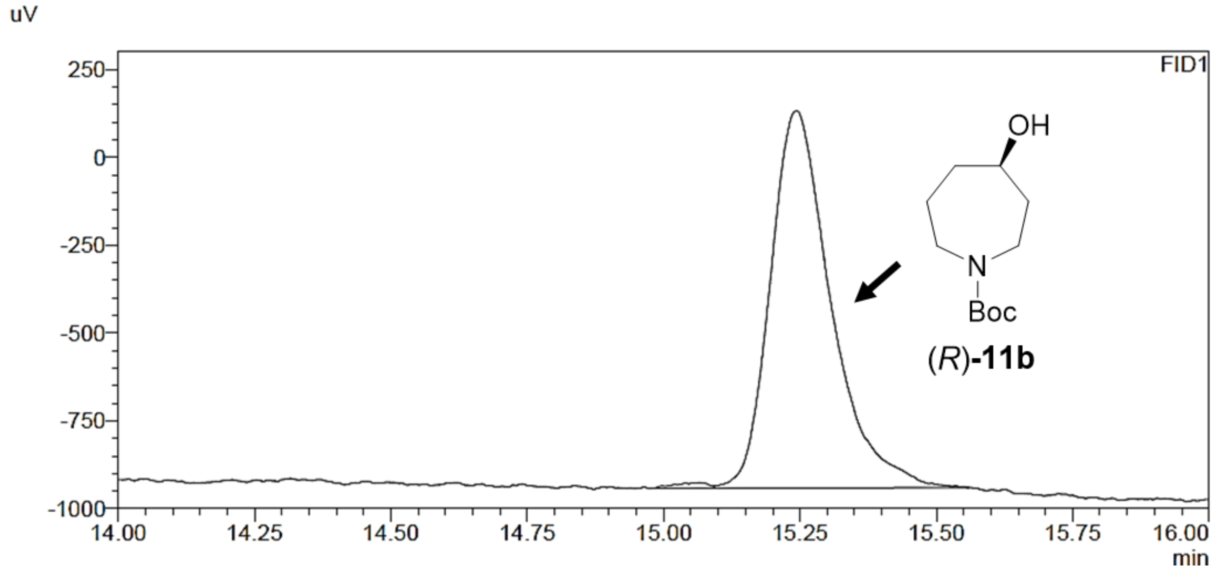

Analyte	RT (min)	% Area
(*S*)-**11b**	15.066	0.8
(*R*)-**11b**	15.243	99.2

Figure S15. GC-chromatogram of standard (*R*)-**11b** | Method 7

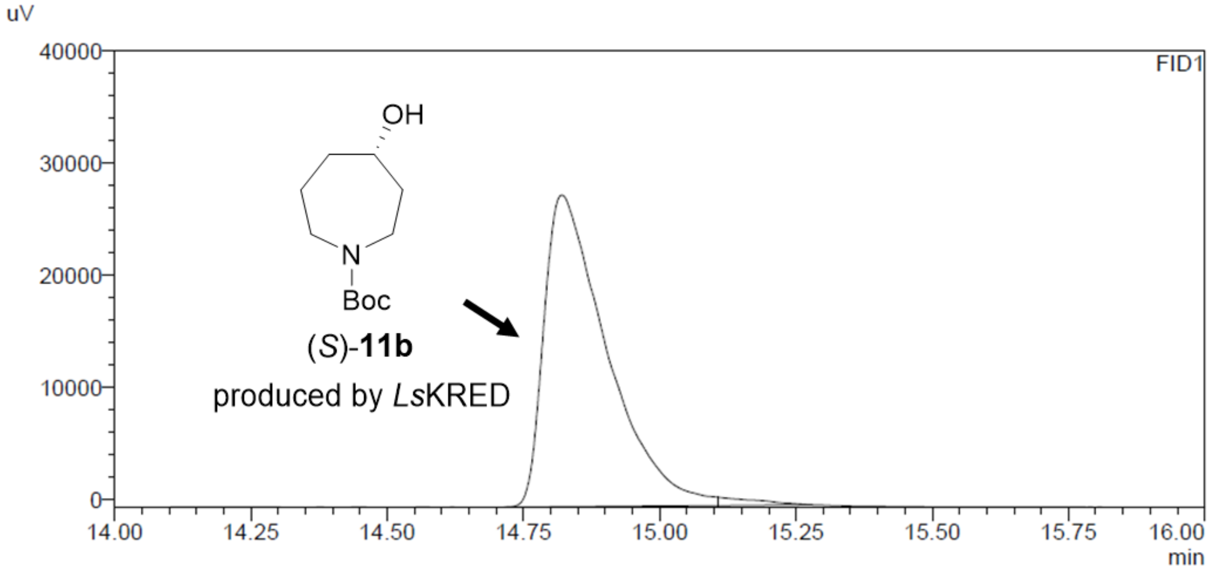

Analyte	RT (min)	% Area
(*S*)-**11b**	14.821	98.85
(*R*)-**11b**	15.140	1.15

Figure S16. GC-chromatogram of enantioenriched **11b**, produced via *Ls*KRED-mediated ketoreduction | Method 7

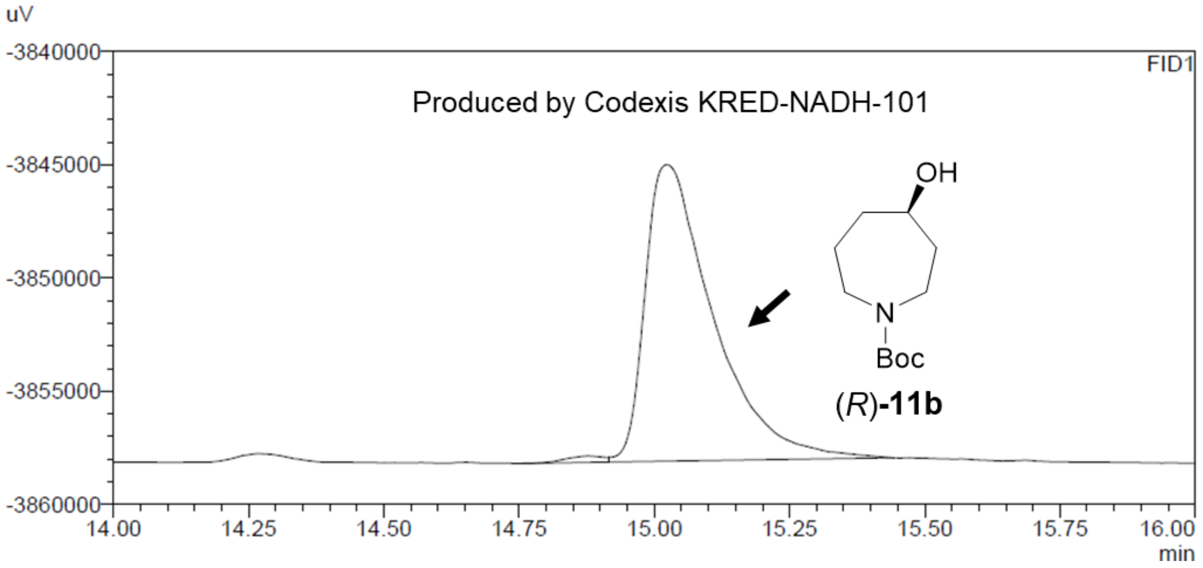

Analyte	RT (min)	% Area
(*S*)-**11b**	14.878	1.1
(*R*)-**11b**	15.023	98.9

Figure S17. GC-chromatogram of enantioenriched **11b**, produced via Codexis KRED-NADH-101-mediated ketoreduction | Method 7

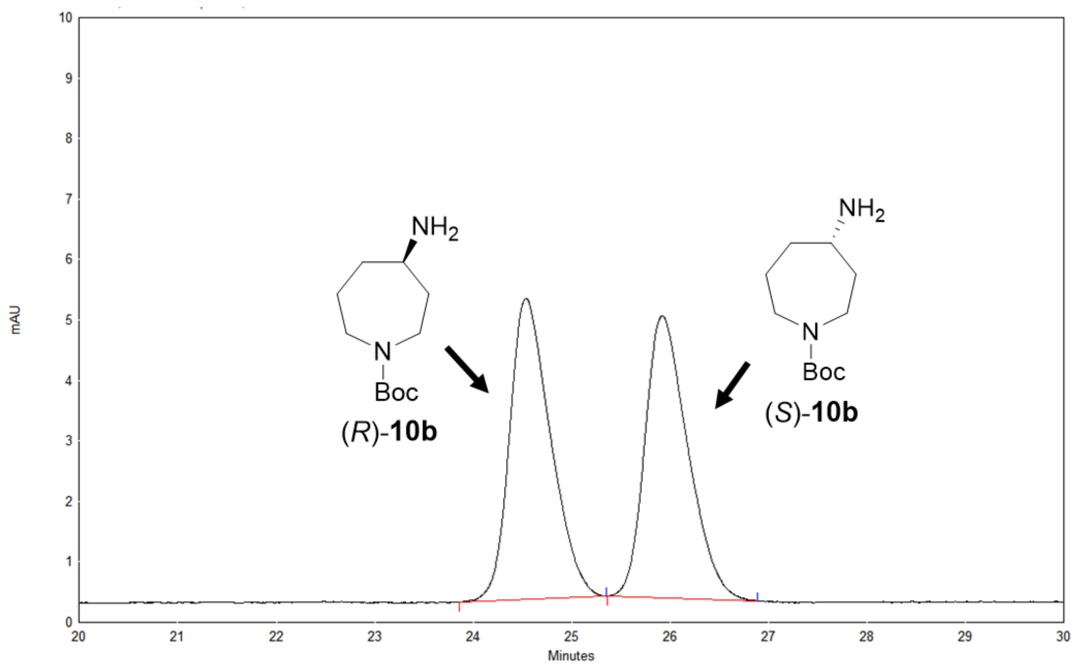

Analyte	RT (min)	% Area
(*R*)-**10b**	24.537	50.5
(*S*)-**10b**	25.920	49.5

Figure S18. HPLC chromatogram of standard (*rac*)-**10b** | Method 8

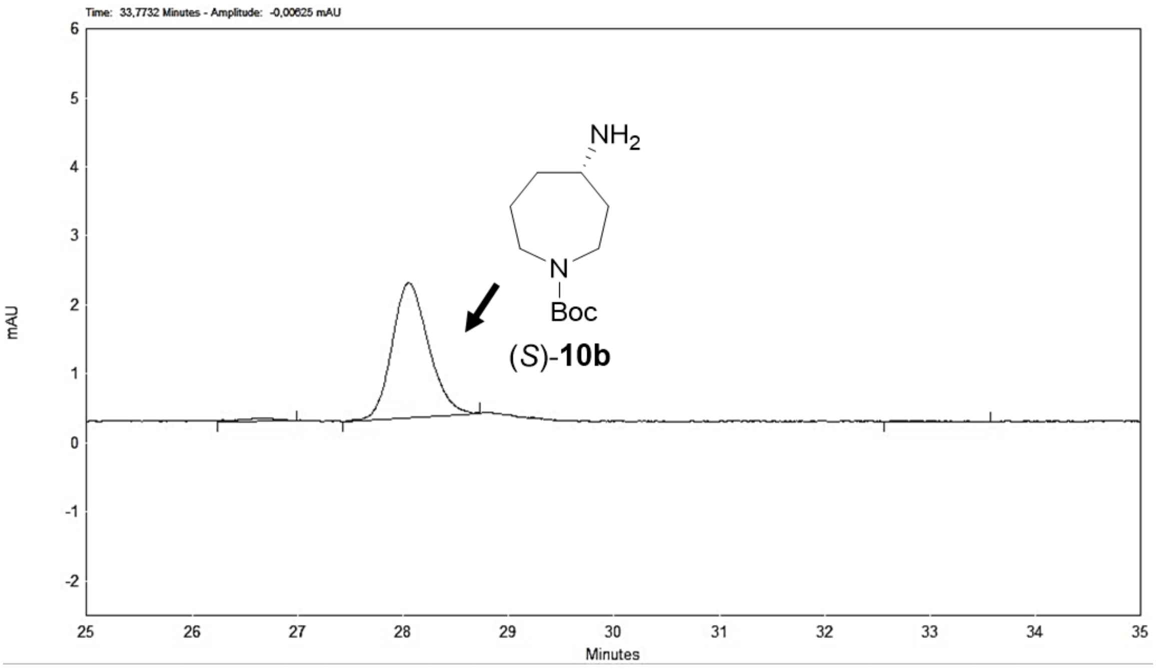

Analyte	RT (min)	% Area
(*R*)-**10b**	26.680	2.18
(*S*)-**10b**	28.057	97.82

Figure S19. HPLC chromatogram of standard (*S*)-**10b** | Method 8

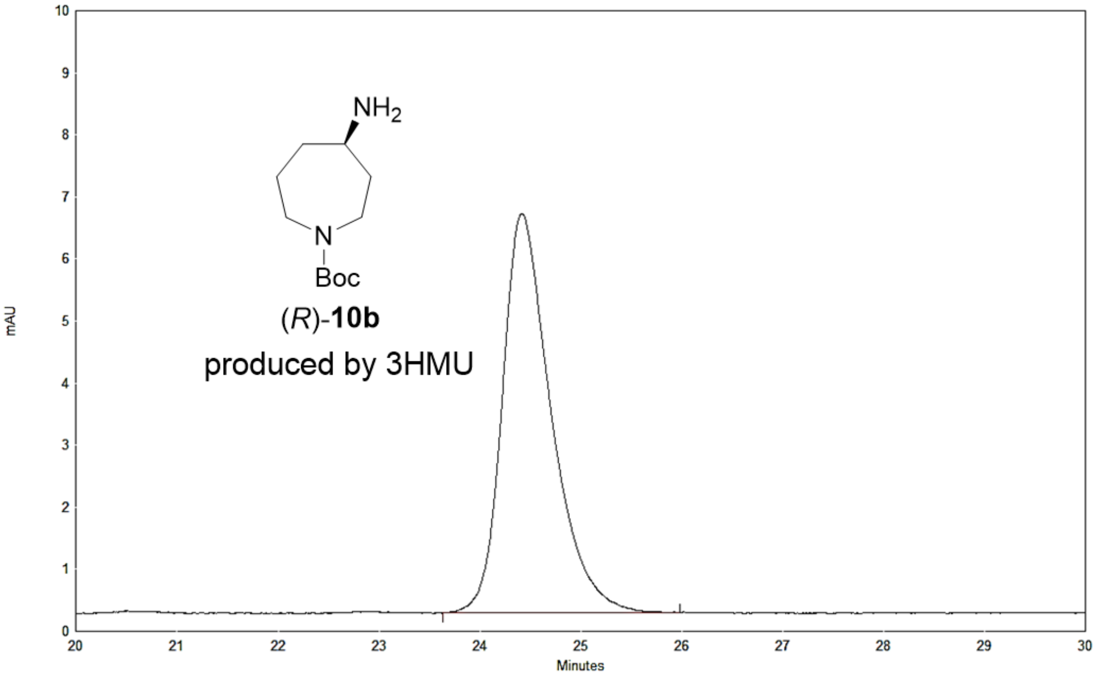

Analyte	RT (min)	% Area
(*R*)-**10b**	24.410	100

Figure S20. HPLC chromatogram of enantioenriched **10b**, produced via 3HMU-mediated transamination | Method 8:

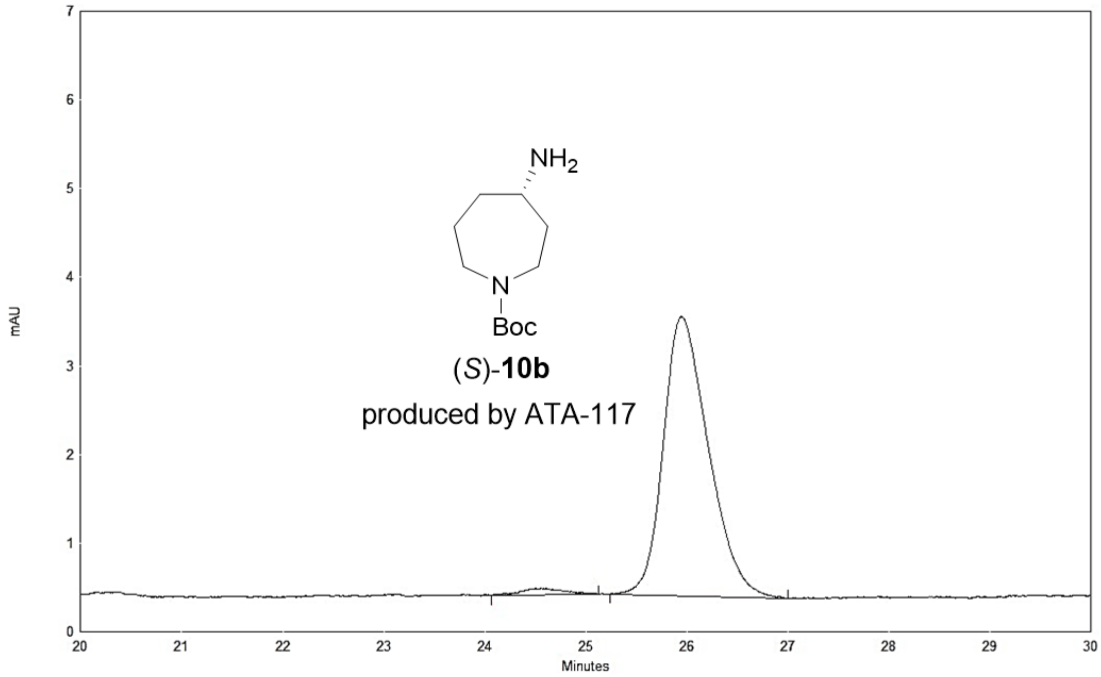

Analyte	RT (min)	% Area
(*R*)-**10b**	24.607	2.5
(*S*)-**10b**	25.940	97.5

Figure S21. HPLC chromatogram of enantioenriched **10b**, produced via ATA-117-mediated transamination | Method 8:

13. Standard compound GC-spectra and GC-based reaction analysis

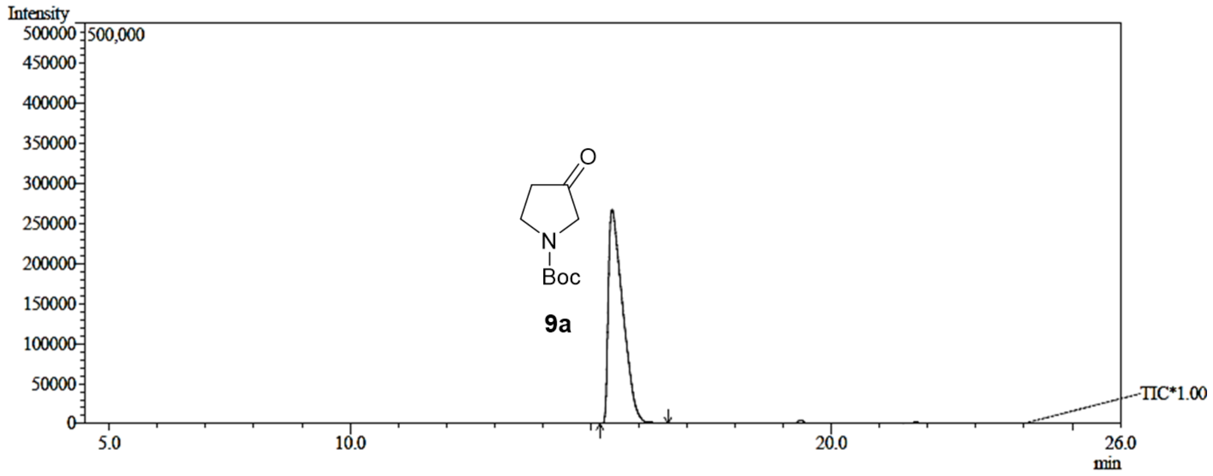

Analyte	RT (min)	% Area
**9a**	15.445	100

Figure S22. GC chromatogram of standard **9a** | Method 1

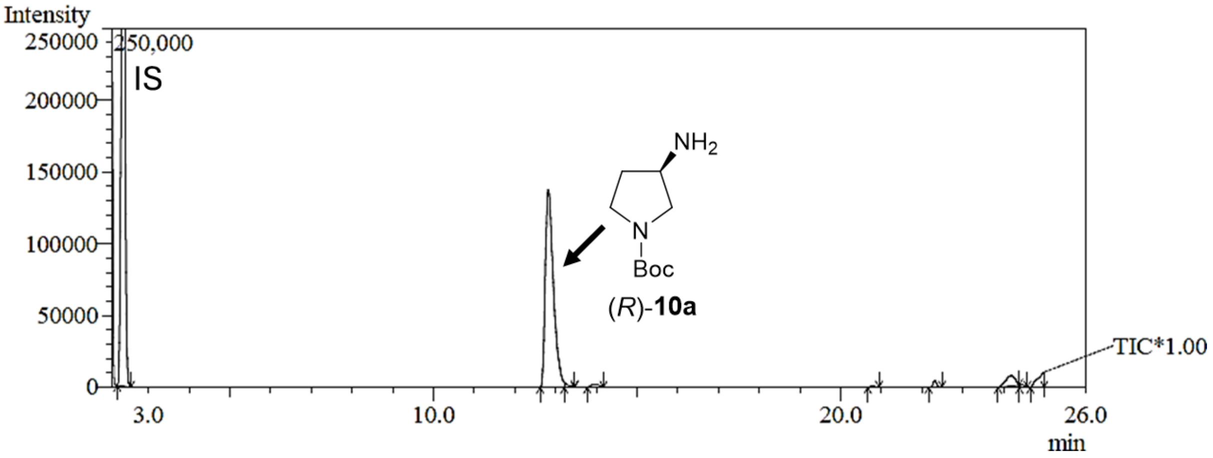

Analyte	RT (min)	% Area
**IS**	2.390	56.36
(*R*)-**10a**	12.822	37.25
(*S*)-**10a**	13.225	0.38
**9a**	13.955	0.55
impurities	20.738-24.992	5.46

Figure S23. GC chromatogram of the photochemoenzymatic conversion of **7a** into (*R*)-**10a** | Method 1

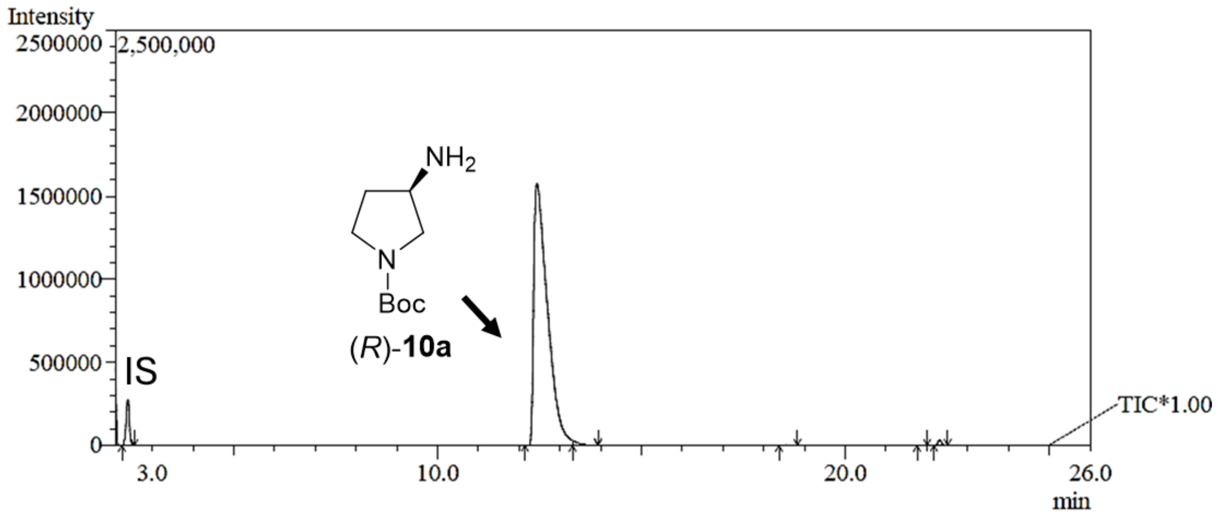

Analyte	RT (min)	% Area
**IS**	2.408	3.86
(*R*)-**10a**	12.436	94.83
(*S*)-**10a**	13.317	0.68
solvent impurities	18.550-22.311	0.65

Figure S24. GC chromatogram of Column-purified (*R*)-**10a** | Method 1

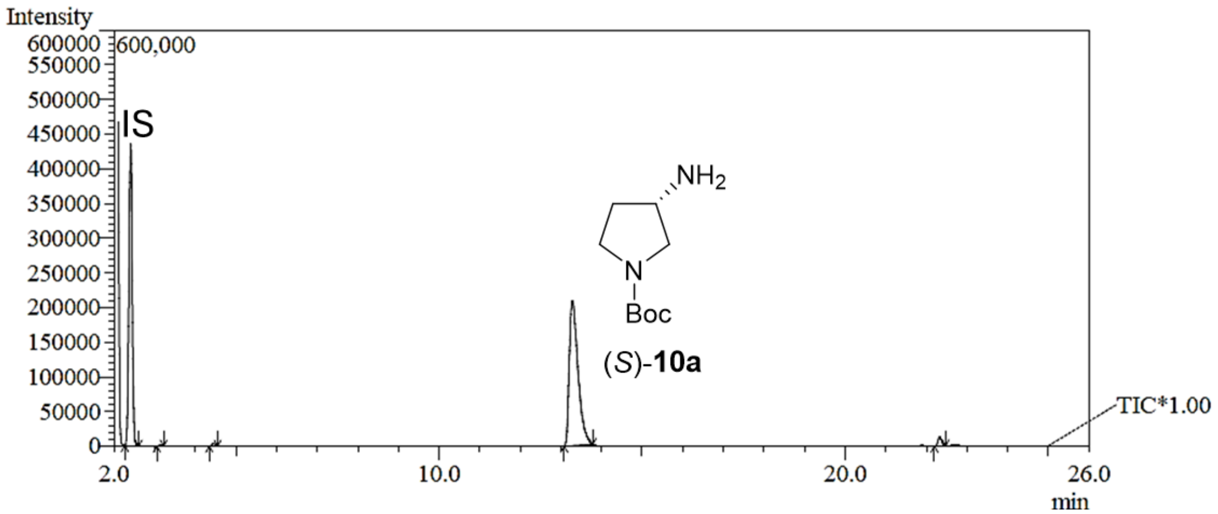

Analyte	RT (min)	% Area
**IS**	2.411	43.02
**impurity**	3.144	0.18
**impurity**	4.451	0.35
(*S*)-**10a**	13.287	54.97
**solvent impurity**	22.328	1.49

Figure S25. GC chromatogram of Column-purified (*S*)-**10a**| Method 1

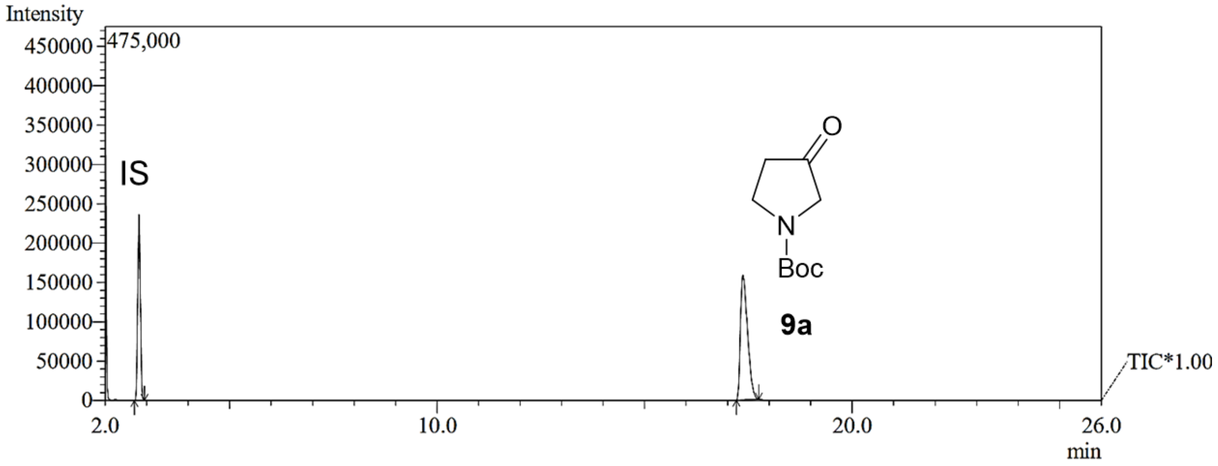

Analyte	RT (min)	% Area
**IS**	2.819	35.76
**9a**	17.368	64.24

Figure S26. GC chromatogram of standard **9a** | Method 2

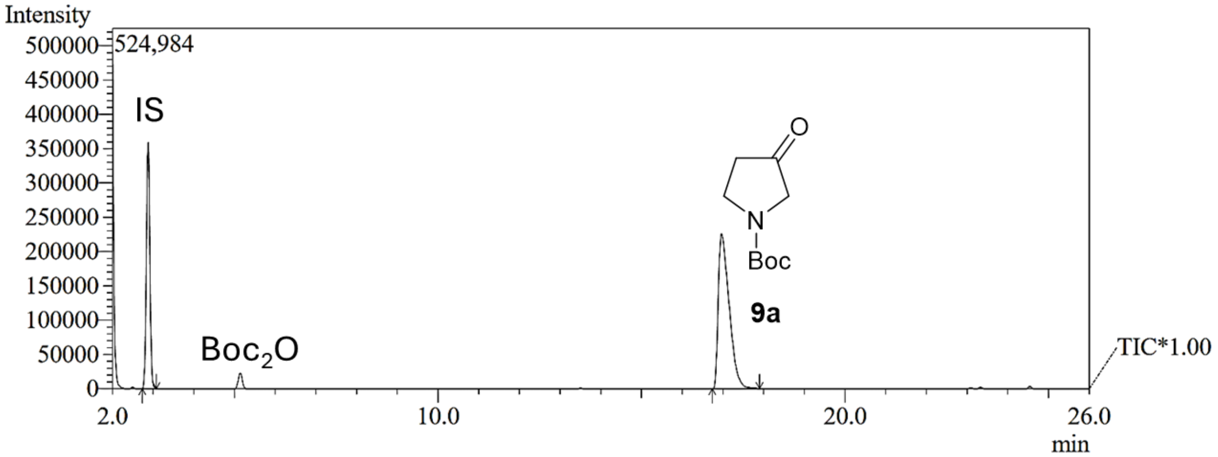

Analyte	RT (min)	% Area
**IS**	2.881	33.83
**Boc_2O**	5.105	7.84
**9a**	16.968	66.17

Figure S27. GC chromatogram of the photochemical conversion of **7a** into **9a** | Method 2

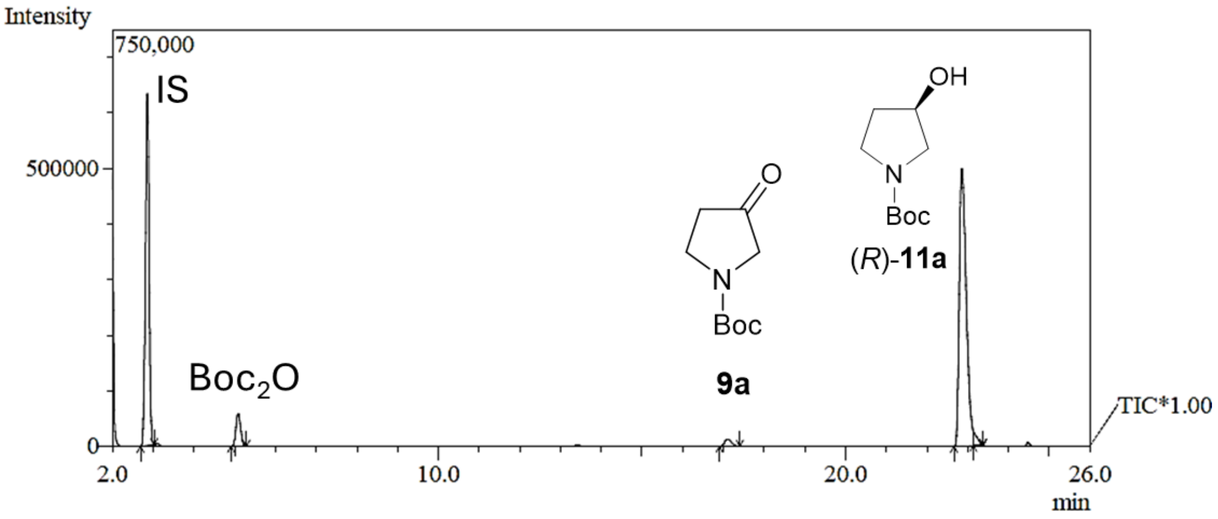

Analyte	RT (min)	% Area
**IS**	2.859	36.37
**Boc_2O**	16.211	4.39
**9a**	17.018	1.43
(*R*)-**11a**	22.861	56.45
(*S*)-**11a**	23.150	1.37

Figure S28. GC chromatogram of the photochemical conversion of **7a** into (*R*)-**11a** | Method 2

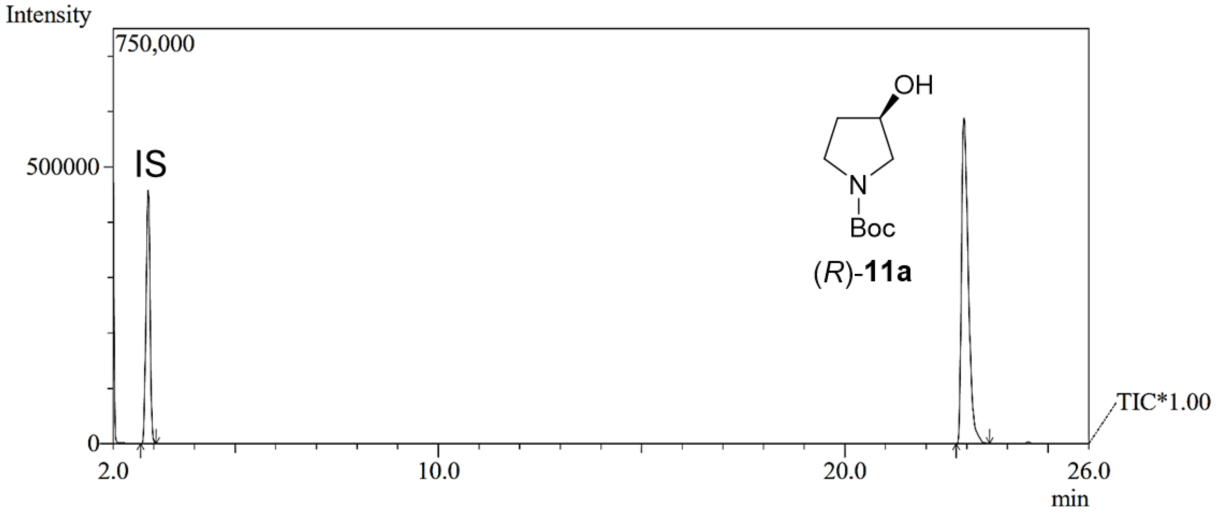

Analyte	RT (min)	% Area
**IS**	2.863	43.76
(*R*)-**11a**	22.932	56.24

Figure S29. GC chromatogram of column-purified (*R*)-**11a** | Method 2:

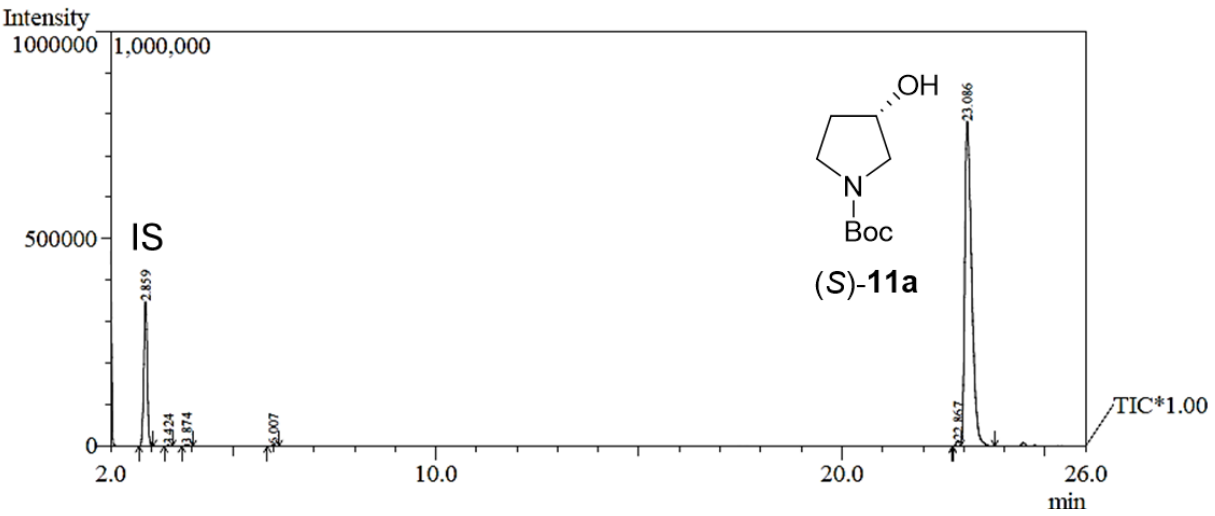

Analyte	RT (min)	% Area
**IS**	2.859	17.75
**impurity**	3.424	0.09
**impurity**	3.874	0.25
**impurity**	6.007	0.19
(*R*)-**11a**	22.867	0.80
(*S*)-**11a**	23.086	80.93

Figure S30. GC chromatogram of column-purified (*S*)-**11a** | Method 2:

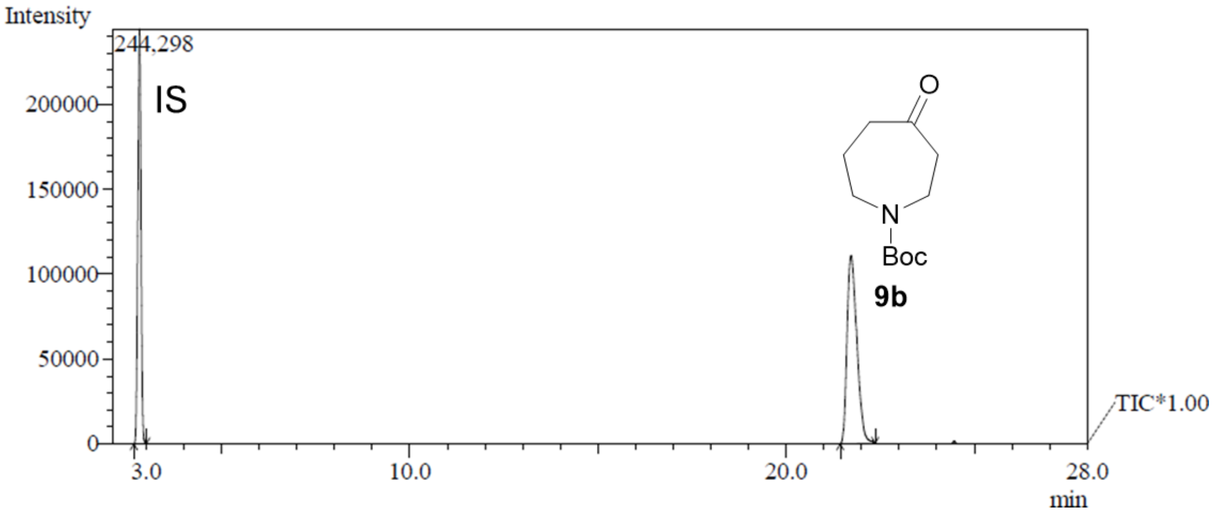

Analyte	RT (min)	% Area
**IS**	2.819	40.63
**9b**	21.728	59.37

Figure S31. GC chromatogram of standard **9b** | Method 7

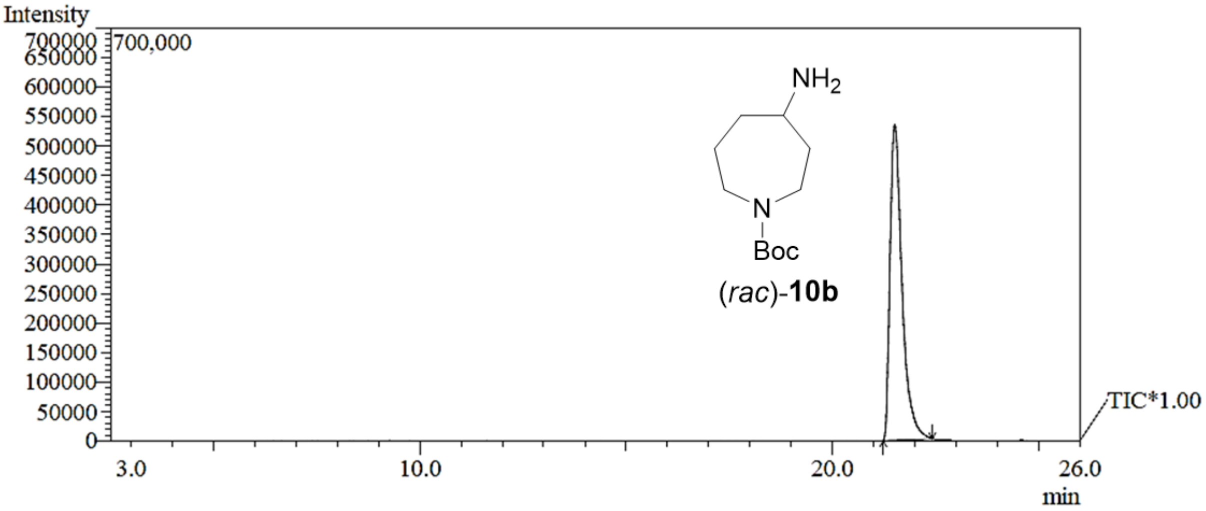

Analyte	RT (min)	% Area
(*rac*)-**10b**	21.516	100

Figure S32. GC chromatogram of standard *rac*-**10b** | stationary phase and GC-temperature program of Method 6 (non-chiral analysis) Here, the GC-temperature program and chiral column of GC Method 6 have been applied, however no chiral separation takes place, and the method has been used only for **10b** product identification.

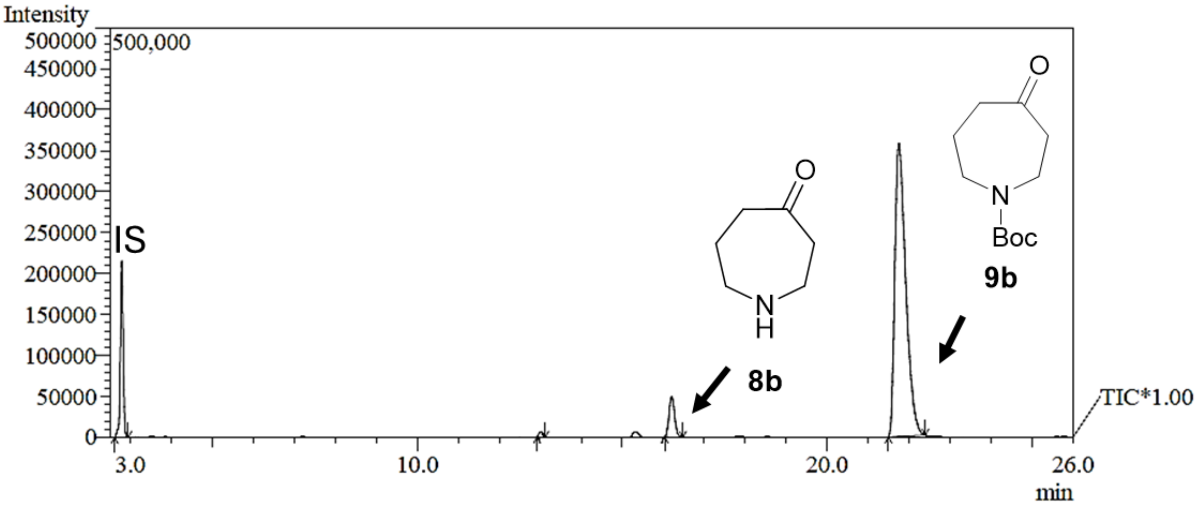

Analyte	RT (min)	% Area
**IS**	2.791	34.21
**8b**	16.211	7.84
**9b**	21.757	56.87

Figure S33. GC chromatogram of standard **9b |** stationary phase and GC-temperature program of Method 5 (non-chiral analysis). Here, the GC-temperature program and chiral column of GC Method 5 have been applied, however no chiral separation takes place, and the method has been used only for **8b** and **9b** product identification.

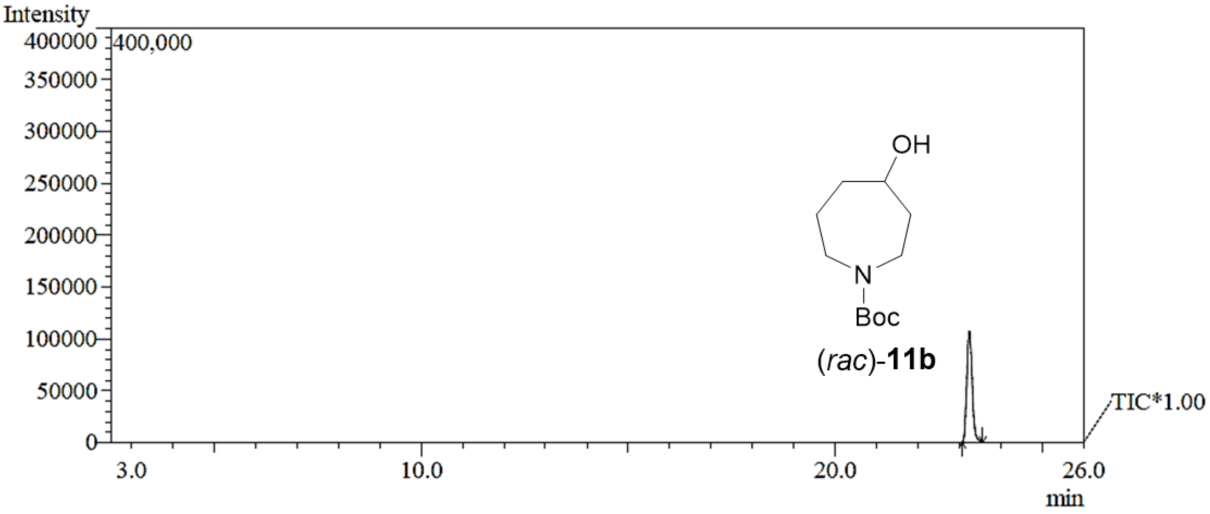

Analyte	RT (min)	% Area
(*rac*)-**11b**	23.243	100

Figure S34. GC chromatogram of standard (*rac*)-**11b** | stationary phase and GC-temperature program of Method 5 (non-chiral analysis). Here, the GC-temperature program and chiral column of GC Method 5 have been applied, however no chiral separation takes place, and the method has been used only for **11b** product identification.

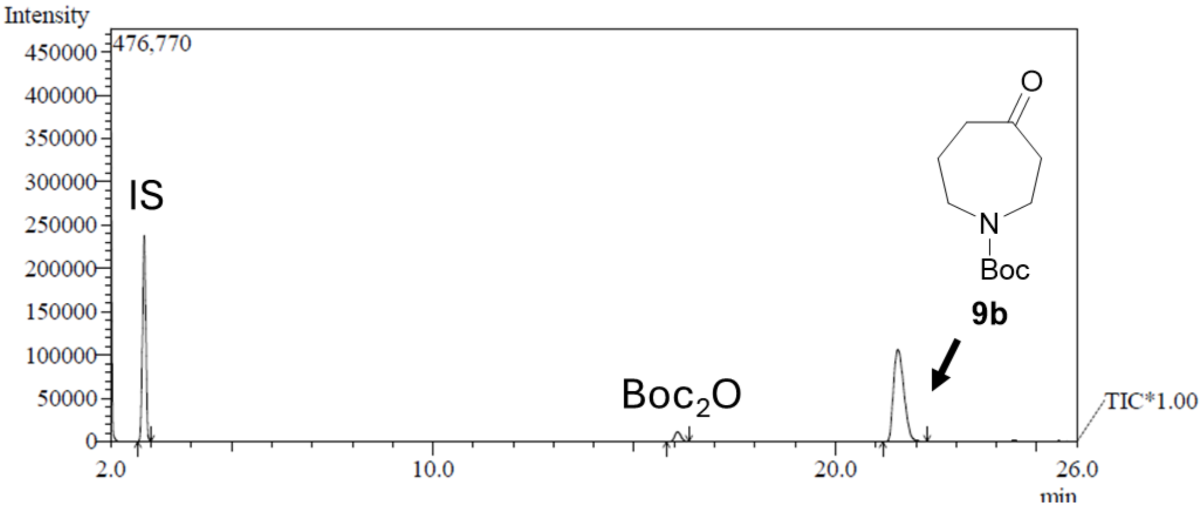

Analyte	RT (min)	% Area
**IS**	2.834	40.3
**Boc_2O**	16.082	3.5
**9b**	21.552	56.2

Figure S35. GC chromatogram photochemical conversion of **7b** into **9b |** stationary phase and GC-temperature program of Method 5 (non-chiral analysis)

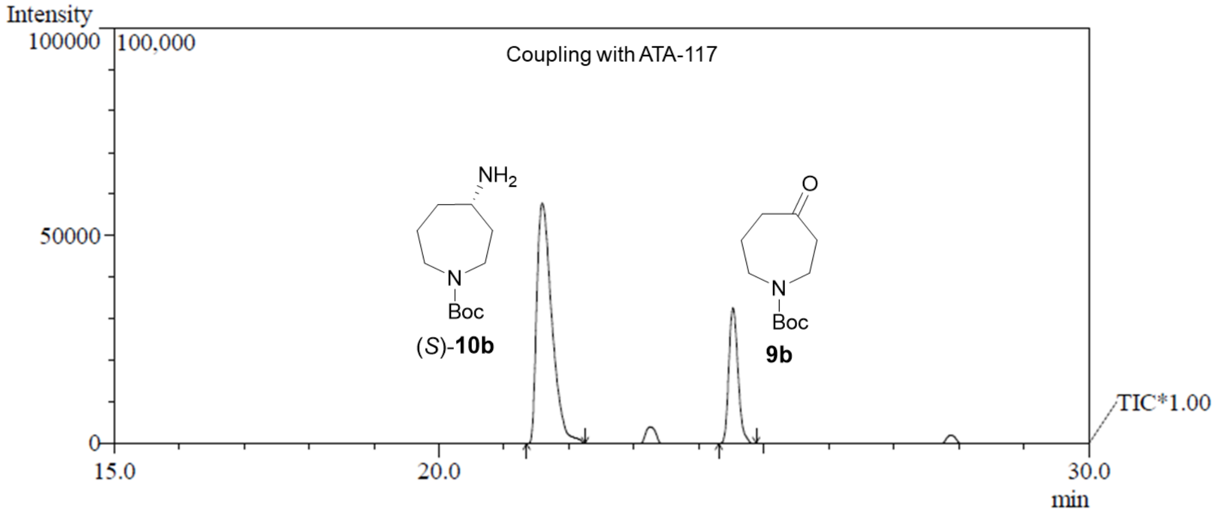

Analyte	RT (min)	% Area
(*S*)-**10b**	21.591	74.4
**9b**	24.524	25.6

Figure S36. GC chromatogram of the photochemoenzymatic conversion of **7b** into (*S*)-**10b** | Method 6. Here, **10b** chirality has been confirmed via (HPLC-) Method 7, but conversion has been detemined via (GC-) Method 6

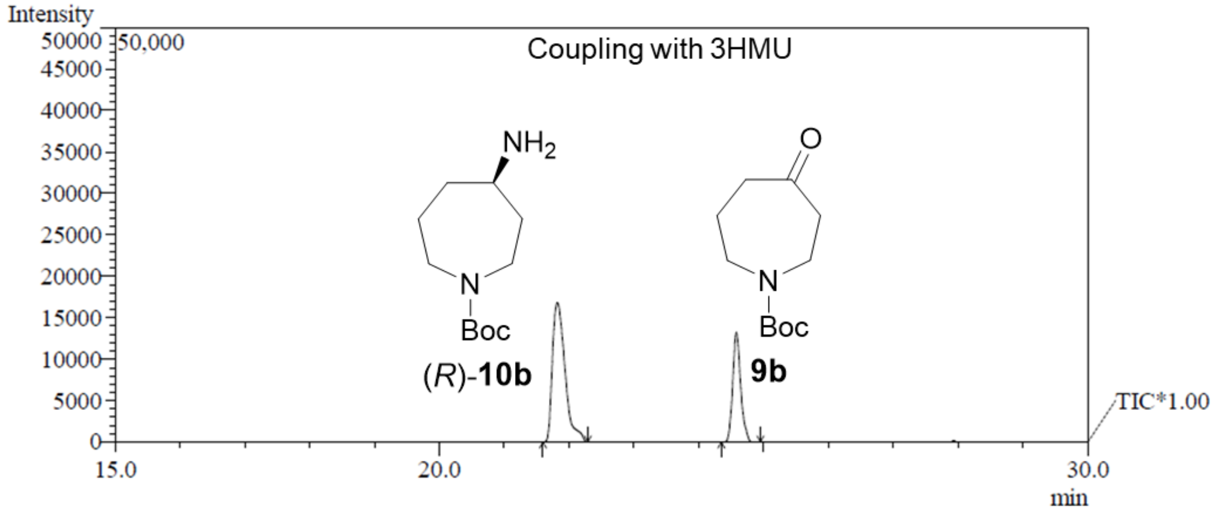

Analyte	RT (min)	% Area
(*R*)-**10b**	21.820	66.9
**9b**	24.581	33.1

Figure **S37.** GC chromatogram of the photochemoenzymatic conversion of **7b** into (*R*)-**10b** | Method 6. Here, **10b** chirality has been confirmed via (HPLC-) Method 7, but conversion has been detemined via (GC-) Method 6

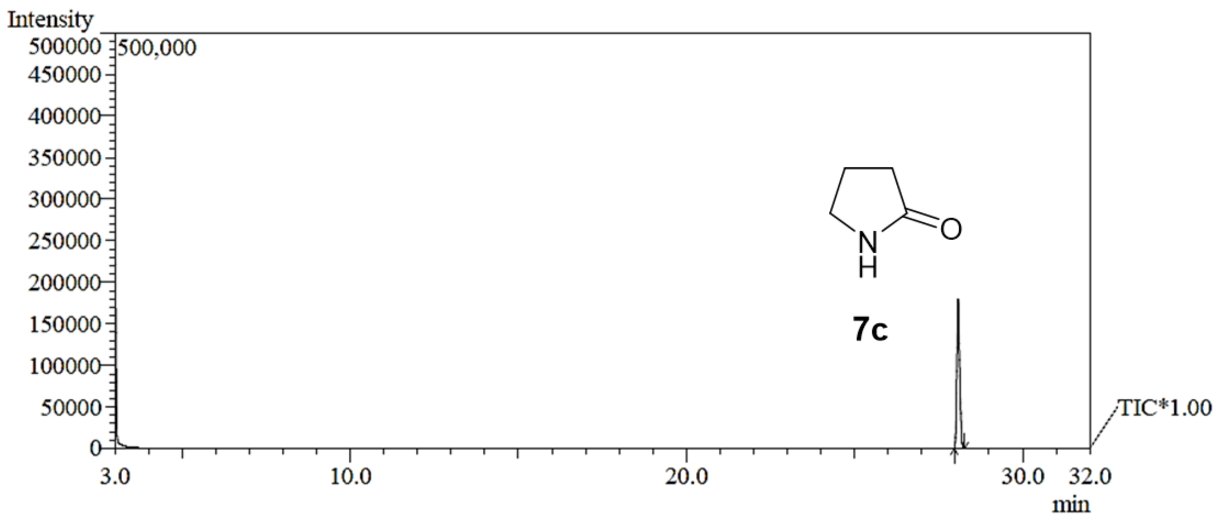

Analyte	RT (min)	% Area
**7c**	28.081	100

Figure S38. GC chromatogram of standard **7c** | Method 8

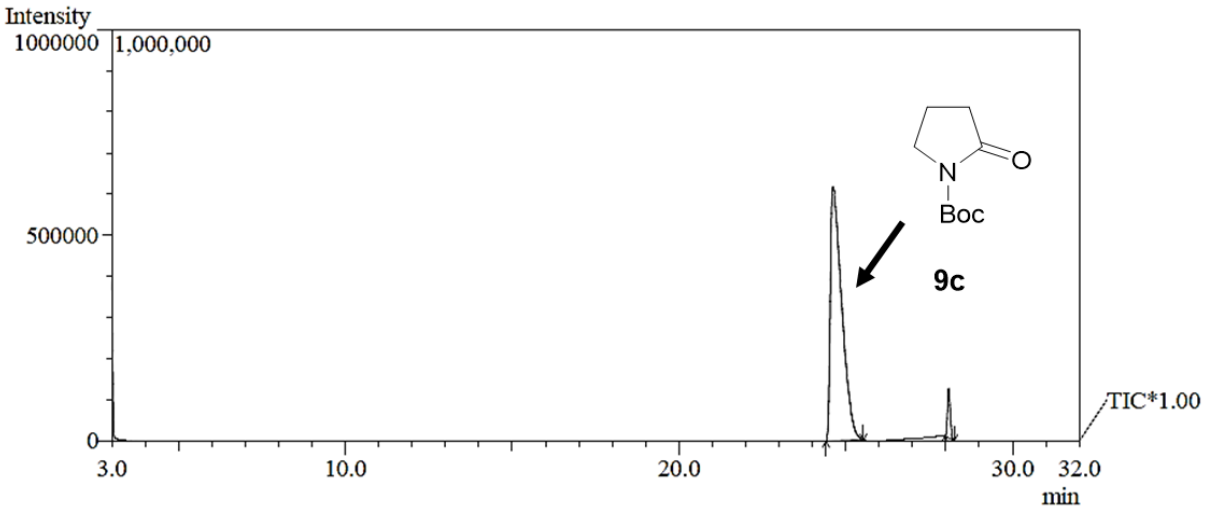

Analyte	RT (min)	% Area
**7c**	28.088	16.23
**9c**	24.623	83.77

Figure S39. GC chromatogram of standard **9c** | Method 8

14. MS-spectra

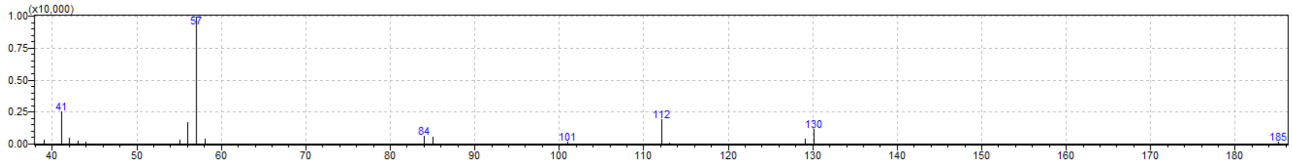

Figure S40. Mass spectrum of **9a**. Exact mass of **9a**: 185,11 g/mol, found: 185 m/z.

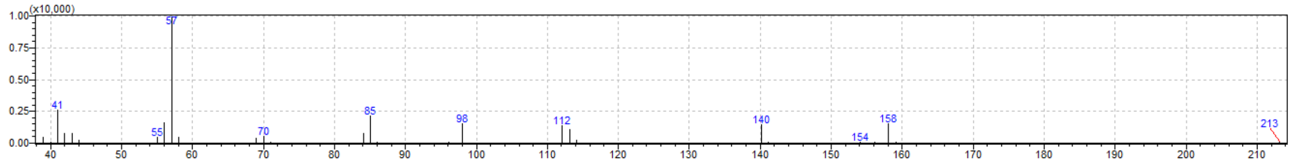

Figure S41. Mass spectrum of **9b.** Exact mass of **9b**: 213,14 g/mol, found: 213 m/z.

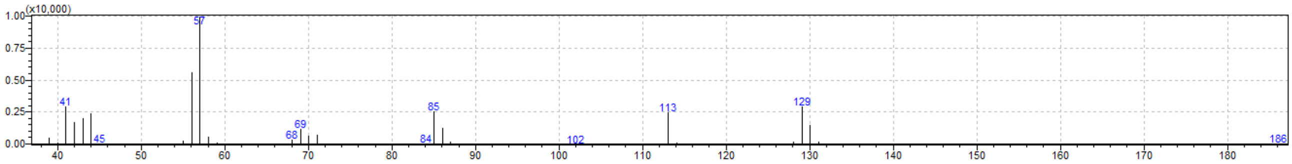

Figure S42. Mass spectrum of (*R*)-**10a.** Exact mass of **10a**: 186.14 g/mol, found: 186 m/z.

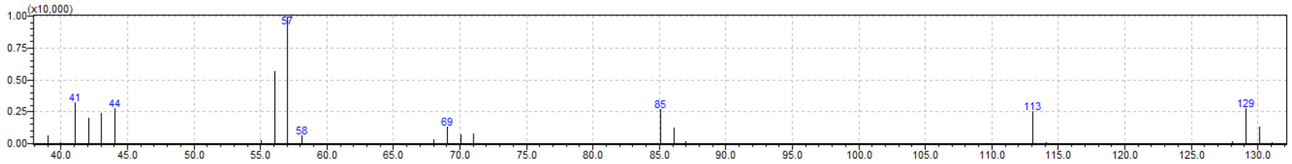

Figure S43. Mass spectrum of (*S*)-**10a.** Exact mass of **10a**: 186.14 g/mol. Signal186 m/z was below limit of detection.

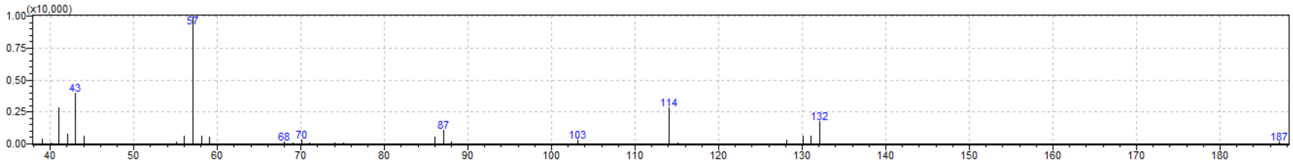

Figure S44. Mass spectrum of (*R*)-**11a.** Exact mass of **11a**: 187.12 g/mol, found: 187 m/z.

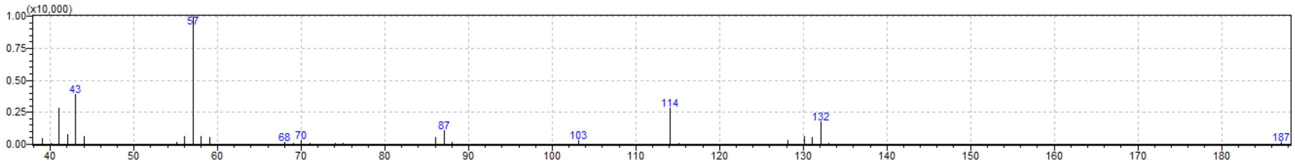

Figure S45. Mass spectrum of (*S*)-**11a.** Exact mass of **11a**: 187.12 g/mol, found: 187 m/z.

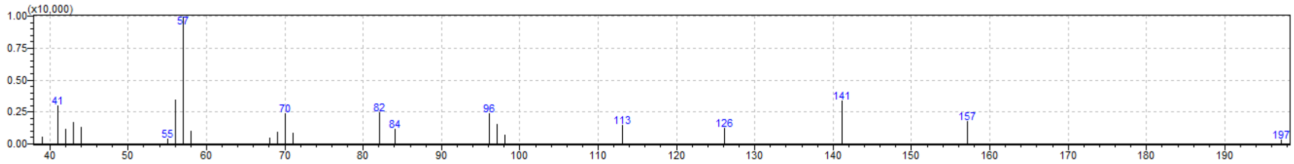

Figure S46. Mass spectrum of **10b**. Exact mass of **10b**: 215,15 g/mol, found: 197 m/z. ($C_{11}H_{19}NO_2{}^{2+}$)

14. NMR spectra

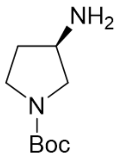

(*R*)-**10a**

^1H NMR (400 MHz, $CDCl_3$)

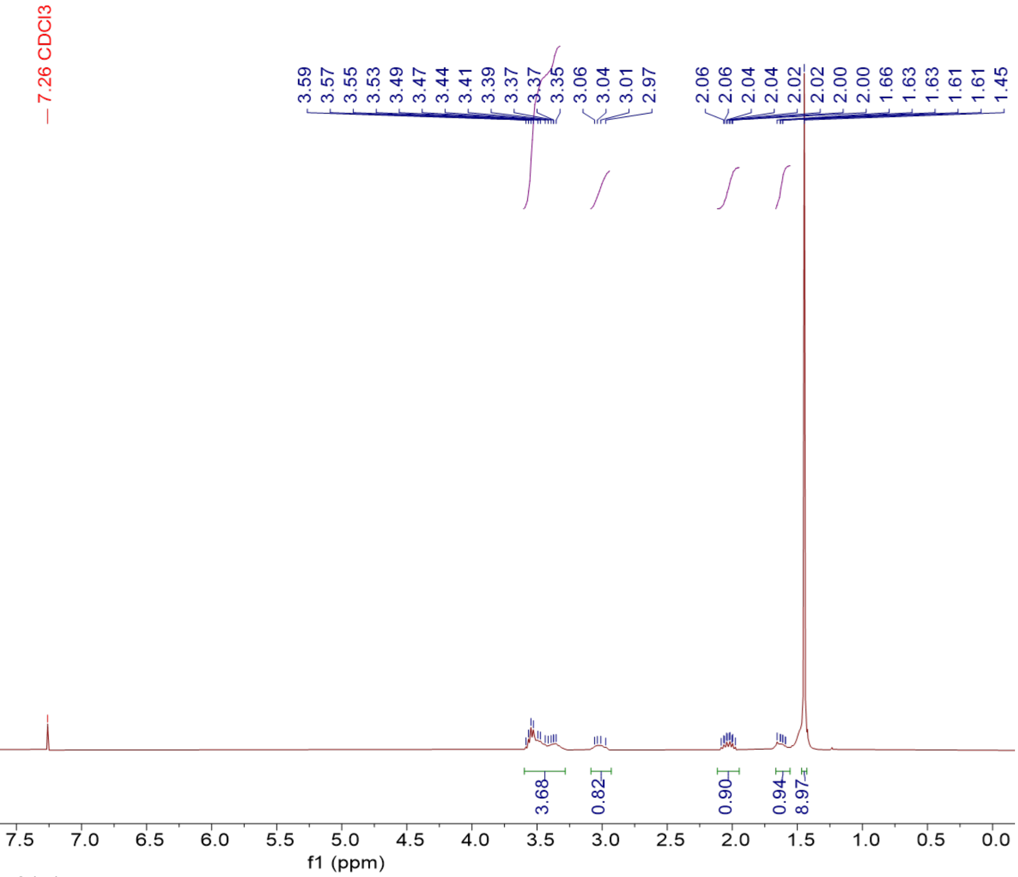

Figure S47. ^1H NMR spectrum of (*R*)-**10a**.

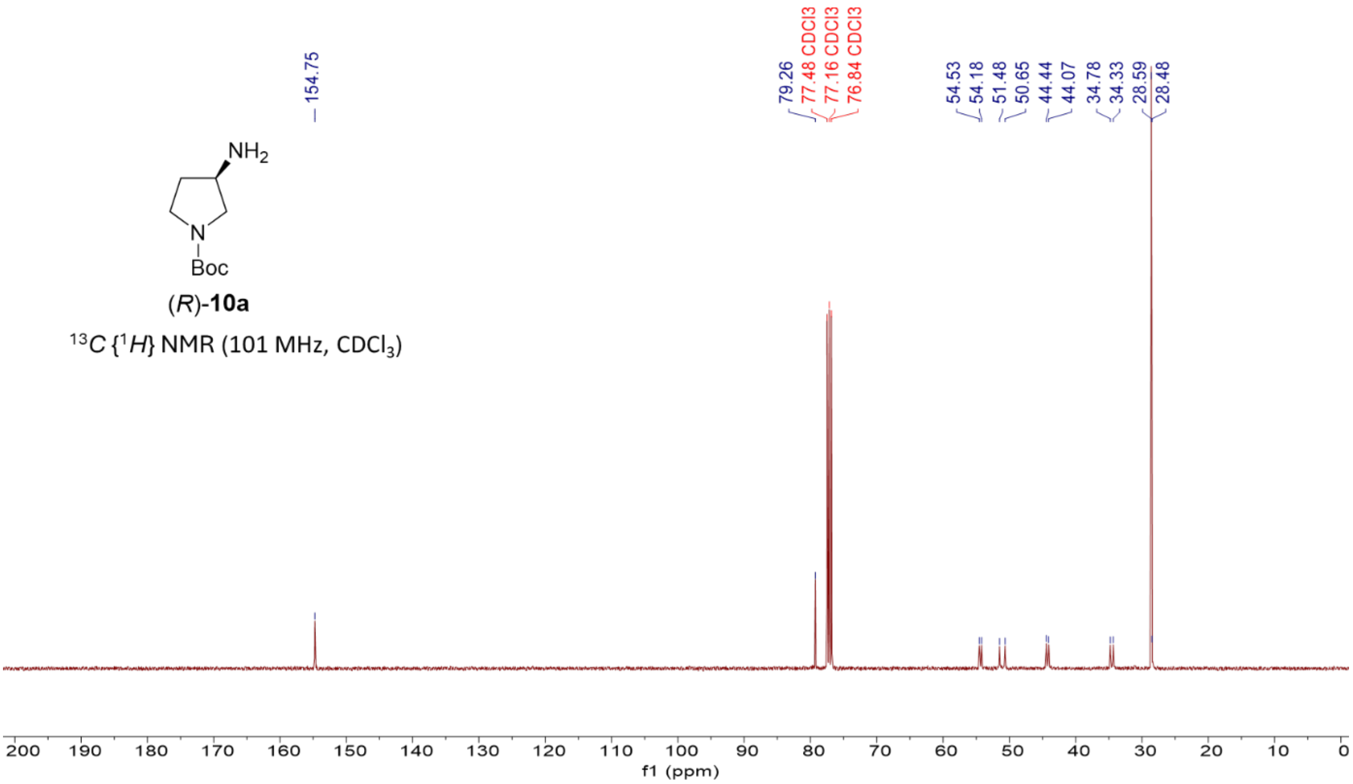

Figure S48. $^{13}C\{^{1}H\}$ NMR spectrum of (*R*)-**10a**.

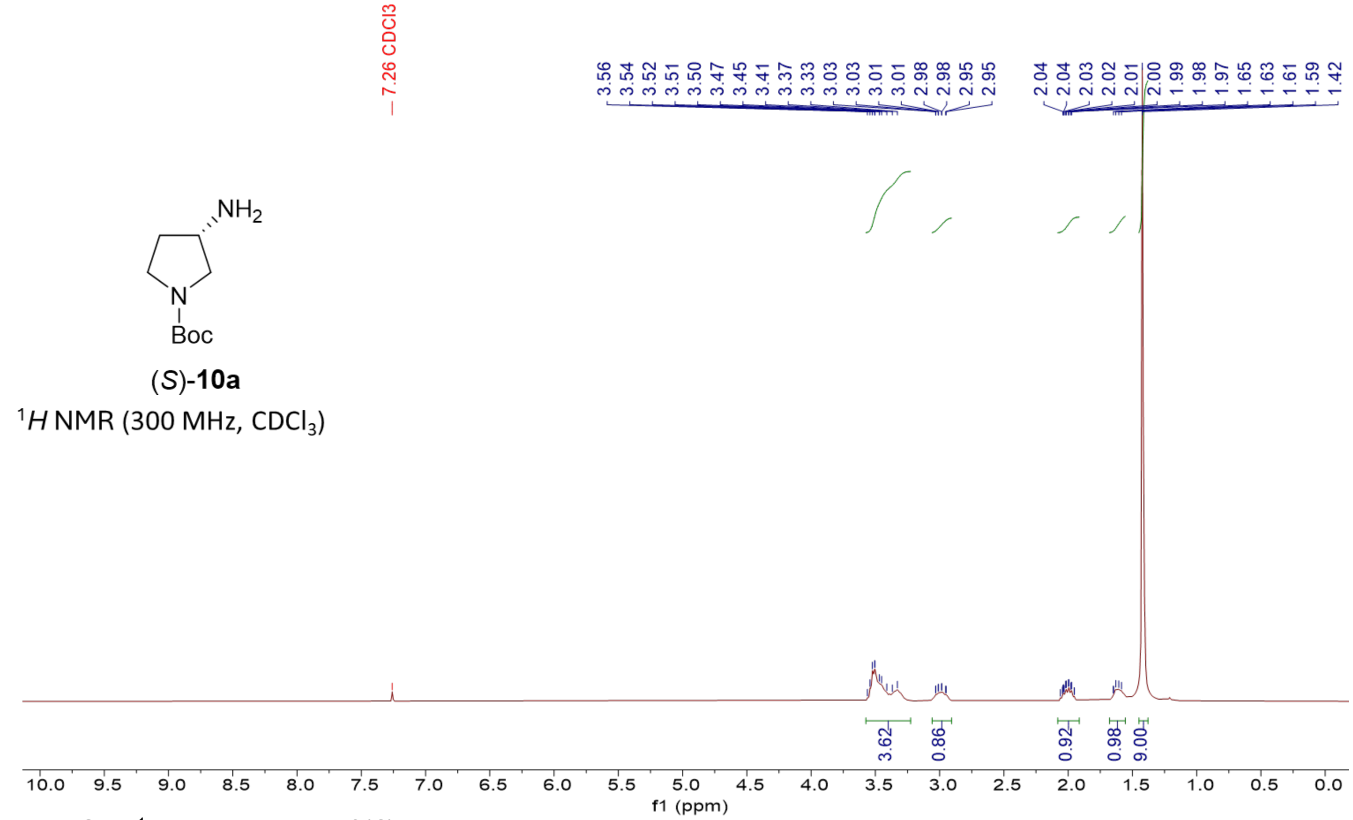

Figure S49. ^{1}H NMR spectrum of (*S*)-**10a**.

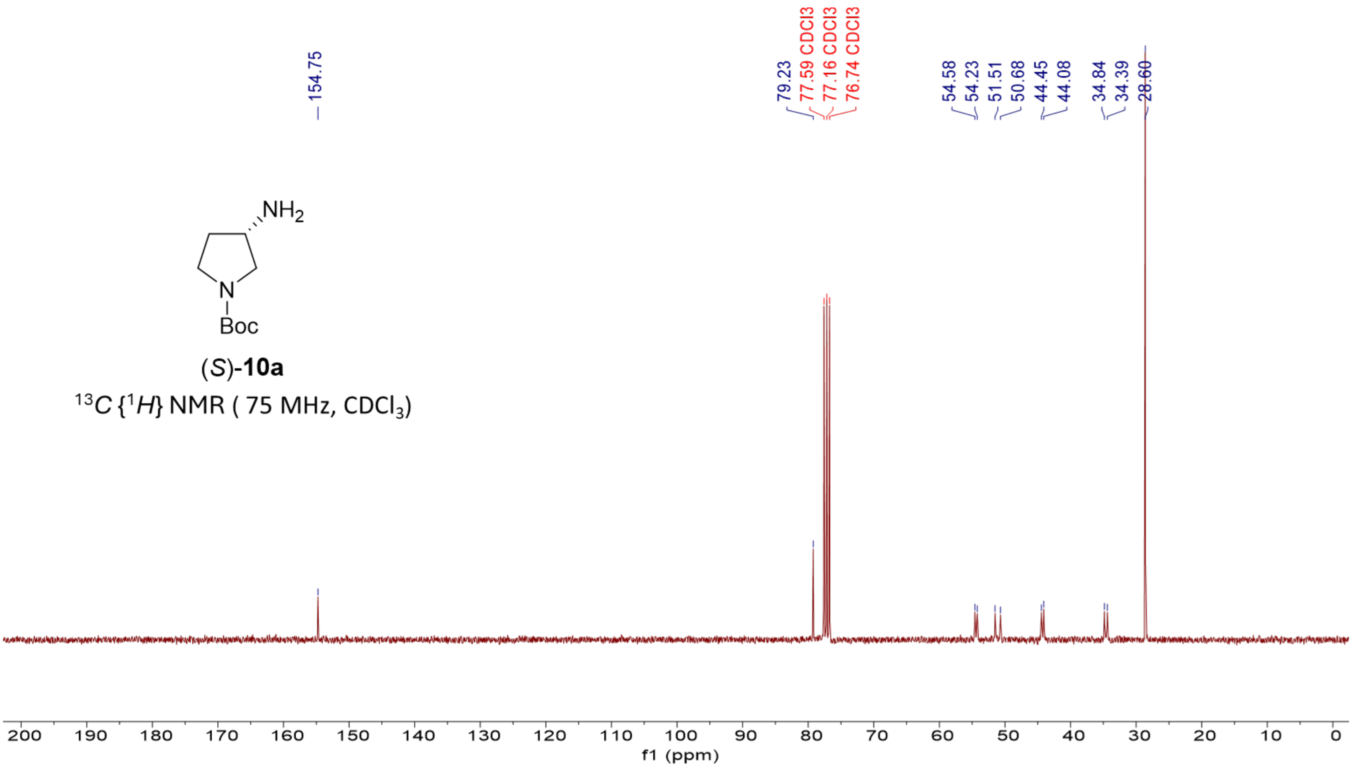

Figure S50. $^{13}C\{^1H\}$ NMR spectrum of (*S*)-**10a**.

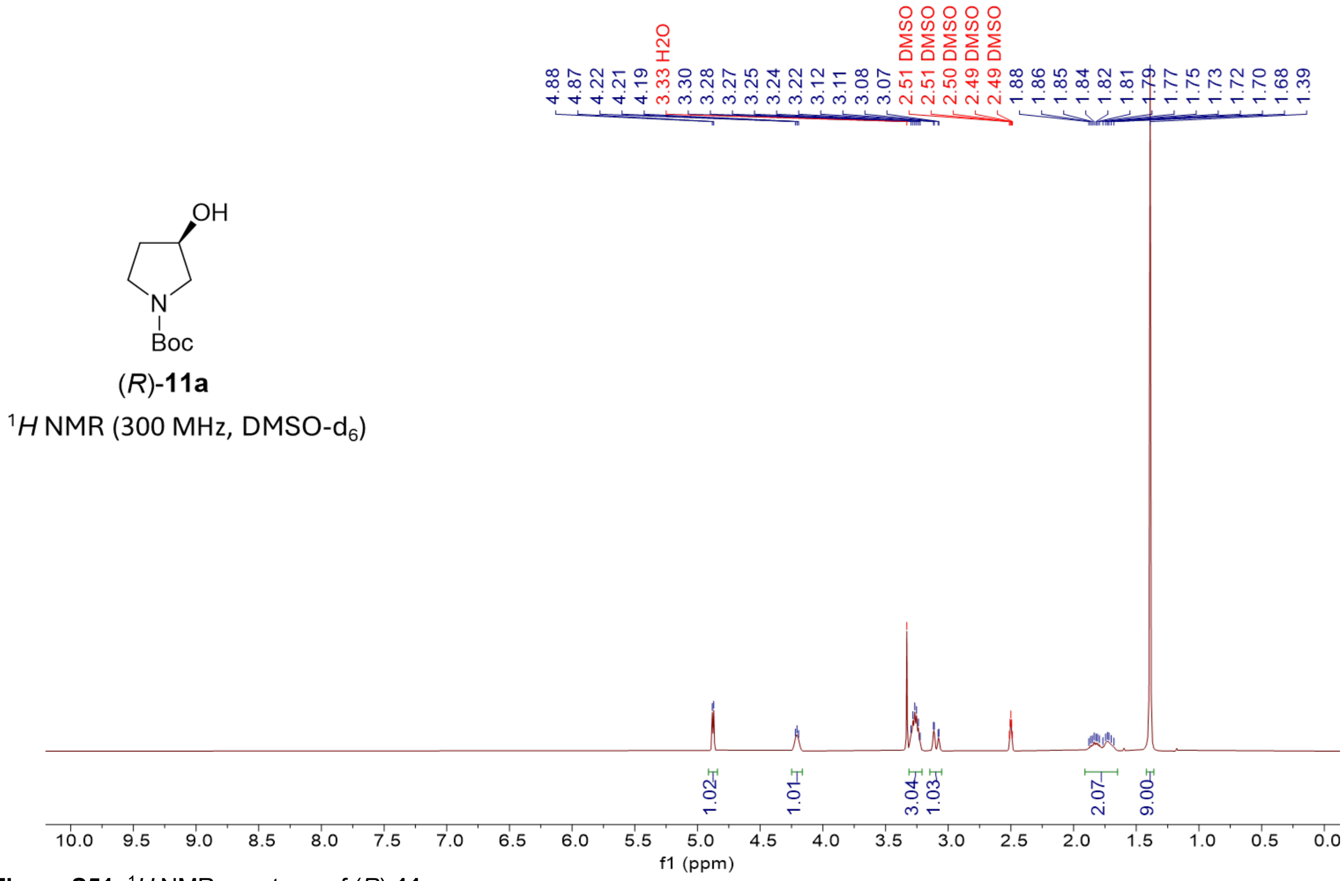

Figure S51. ^1H NMR spectrum of (*R*)-**11a**.

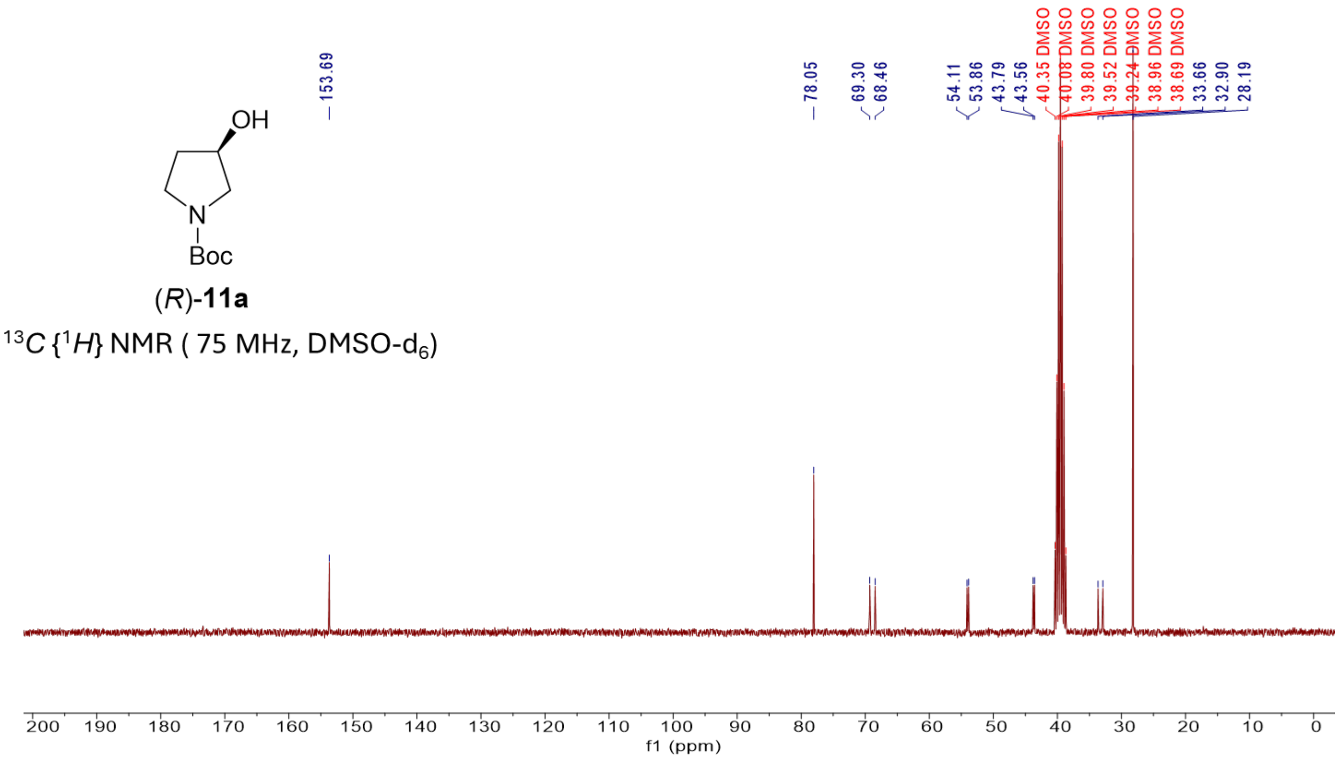

Figure S52. $^{13}C\{^{1}H\}$ NMR spectrum of (*R*)-**11a**.

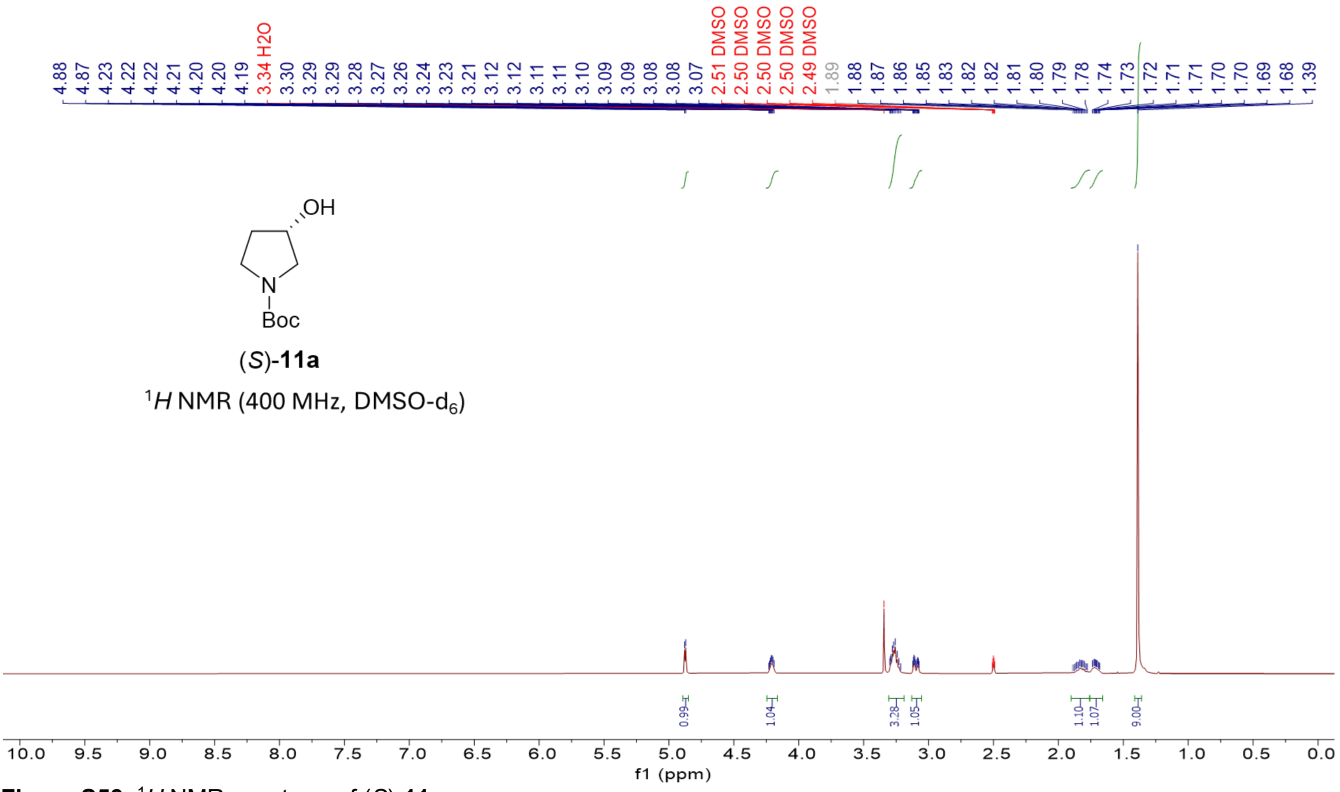

Figure S53. ^{1}H NMR spectrum of (*S*)-**11a**.

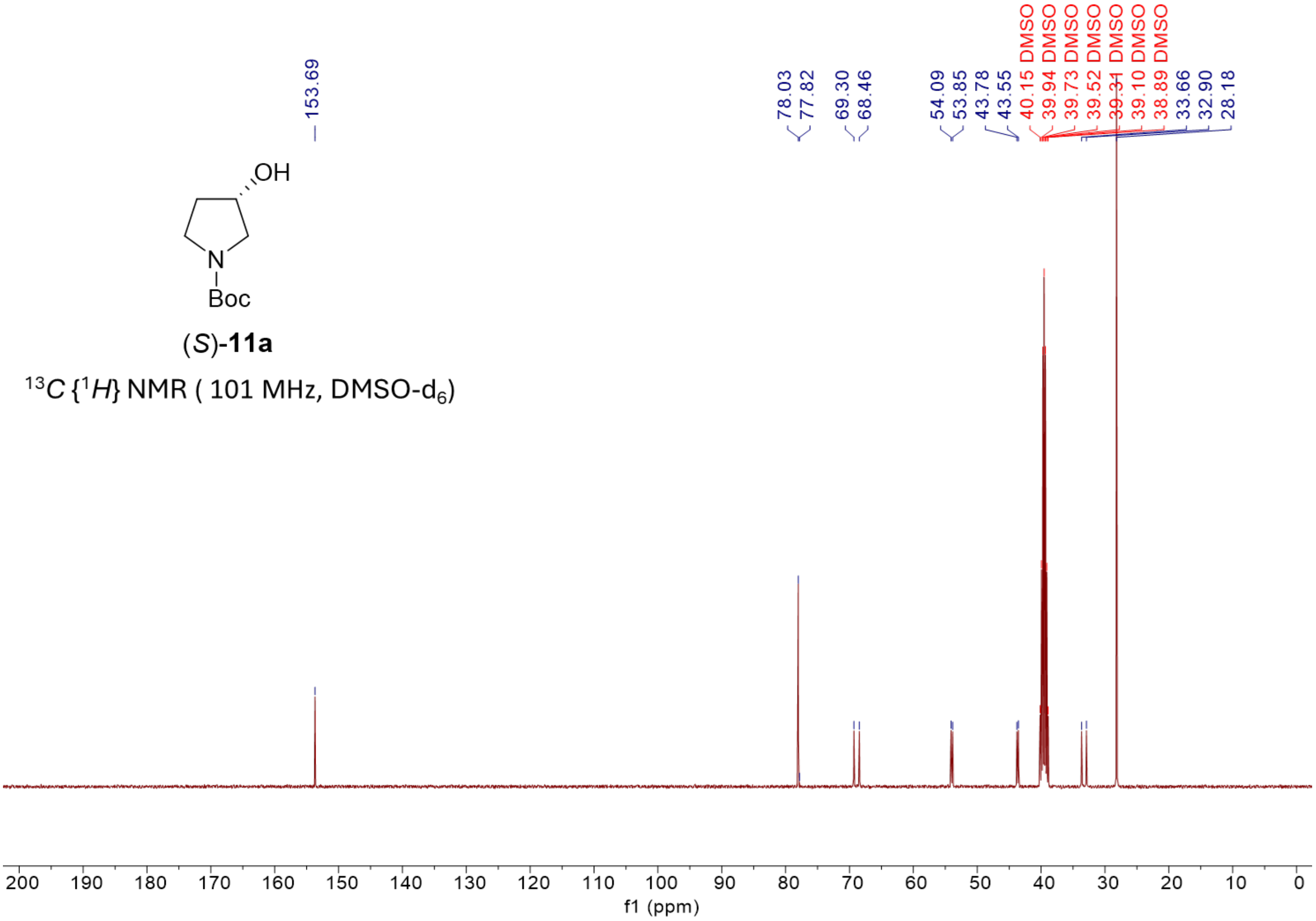

Figure S54. $^{13}C\{^{1}H\}$ NMR spectrum of (*S*)-**11a**.

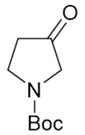

9a

^{1}H NMR (400 MHz, $CDCl_3$)

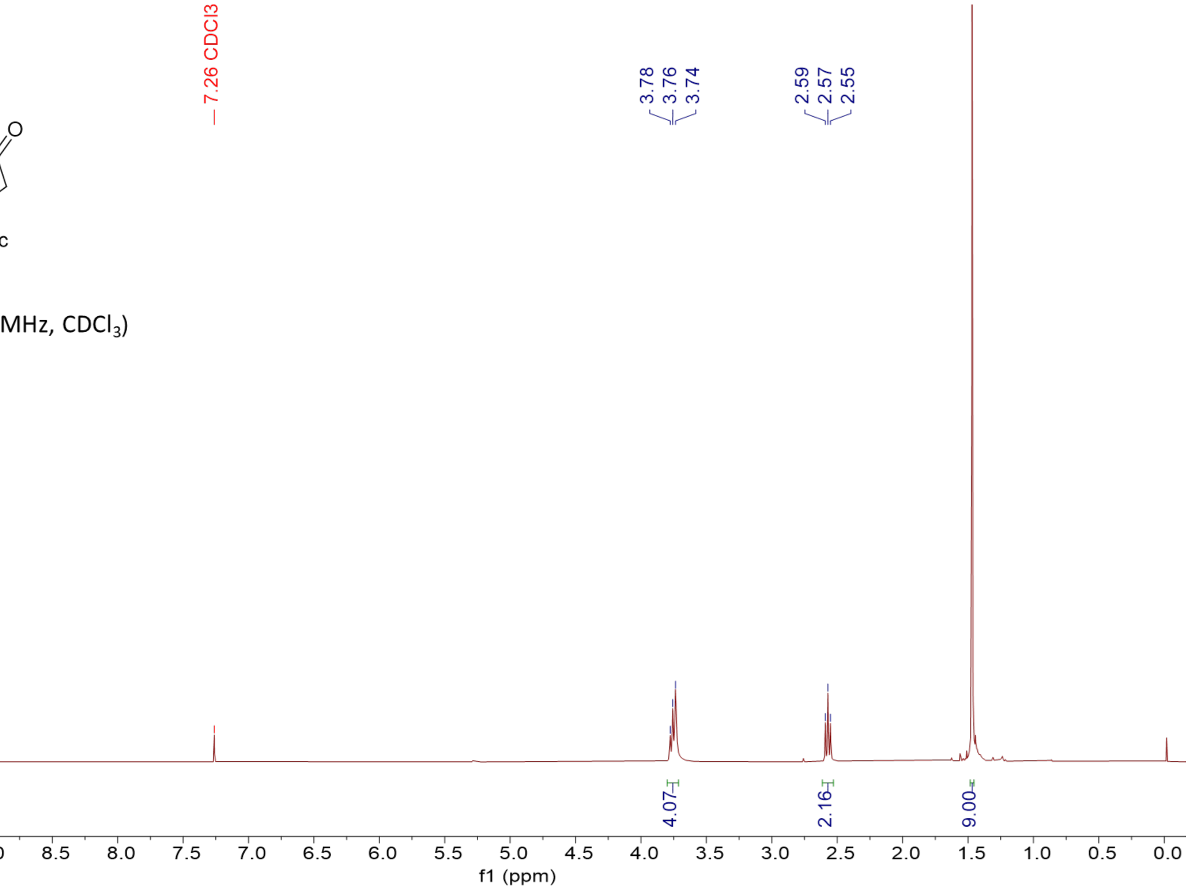

Figure S55. ^{1}H NMR spectrum of **9a**.

16. References

(1) Gottlieb, H. E.; Kotlyar, V.; Nudelman, A. NMR Chemical Shifts of Common Laboratory Solvents as Trace Impurities. *J. Org. Chem.* **1997**, *62* (21), 7512–7515. https://doi.org/10.1021/jo971176v.

(2) SnapGene Plasmids webpage. https://www.snapgene.com/plasmids/pet_and_duet_vectors_(novagen)/pET-28b(%8b) (accessed 2024-07-02)

(3) SnapGene Plasmids webpage. https://www.snapgene.com/plasmids/pet_and_duet_vectors_(novagen)/pET-210b(%8b) (accessed 2024-07-02)

(4) SnapGene Plasmids webpage. https://www.snapgene.com/plasmids/pet_and_duet_vectors_(novagen)/pET-28b(%8b) (accessed 2024-07-02)

(5) SnapGene Plasmids webpage. https://www.snapgene.com/plasmids/pet_and_duet_vectors_(novagen)/pET-28a(%8b) (accessed 2024-07-02)

(6) Steffen-Munsberg, F.; Vickers, C.; Thontowi, A.; Schätzle, S.; Tumlirsch, T.; Svedendahl Humble, M.; Land, H.; Berglund, P.; Bornscheuer, U. T.; Höhne, M. Connecting Unexplored Protein Crystal Structures to Enzymatic Function. *ChemCatChem* **2013**, *5* (1), 150–153. https://doi.org/10.1002/cctc.201200544.

(7) Iwasaki, A.; Matsumoto, K.; Hasegawa, J.; Yasohara, Y. A Novel Transaminase, (R)-Amine:Pyruvate Aminotransferase, from Arthrobacter Sp. KNK168 (FERM BP-5228): Purification, Characterization, and Gene Cloning. *Appl. Microbiol. Biotechnol.* **2012**, *93* (4), 1563–1573. https://doi.org/10.1007/s00253-011-3580-0.

(8) Savile, C. K.; Janey, J. M.; Mundorff, E. C.; Moore, J. C.; Tam, S.; Jarvis, W. R.; Colbeck, J. C.; Krebber, A.; Fleitz, F. J.; Brands, J.; Devine, P. N.; Huisman, G. W.; Hughes, G. J. Biocatalytic Asymmetric Synthesis of Chiral Amines from Ketones Applied to Sitagliptin Manufacture. *Science* **2010**, *329* (5989), 305–309. https://doi.org/10.1126/science.1188934.

(9) Guan, L.-J.; Ohtsuka, J.; Okai, M.; Miyakawa, T.; Mase, T.; Zhi, Y.; Hou, F.; Ito, N.; Iwasaki, A.; Yasohara, Y.; Tanokura, M. A New Target Region for Changing the Substrate Specificity of Amine Transaminases. *Sci. Rep.* **2015**, *5* (1), 10753. https://doi.org/10.1038/srep10753.

(10) Thomsen, M.; Skalden, L.; Palm, G. J.; Höhne, M.; Bornscheuer, U. T.; Hinrichs, W. Crystallographic Characterization of the (*R*)-Selective Amine Transaminase from Aspergillus Fumigatus. Acta Crystallogr. D Biol. Crystallogr. **2014**, 70 (4), 1086–1093. https://doi.org/10.1107/S1399004714001084.

(11) Skalden, L.; Thomsen, M.; Höhne, M.; Bornscheuer, U. T.; Hinrichs, W. Structural and Biochemical Characterization of the Dual Substrate Recognition of the (*R*)-selective Amine Transaminase from *Aspergillus Fumigatus*. *FEBS J.* **2015**, *282* (2), 407–415. https://doi.org/10.1111/febs.13149.

(12) Łyskowski, A.; Gruber, C.; Steinkellner, G.; Schürmann, M.; Schwab, H.; Gruber, K.; Steiner, K. Crystal Structure of an (R)-Selective ω-Transaminase from Aspergillus Terreus. *PLoS ONE* **2014**, *9* (1), e87350. https://doi.org/10.1371/journal.pone.0087350.

(13) Wu, S.; Xiang, C.; Zhou, Y.; Khan, M. S. H.; Liu, W.; Feiler, C. G.; Wei, R.; Weber, G.; Höhne, M.; Bornscheuer, U. T. A Growth Selection System for the Directed Evolution of Amine-Forming or Converting Enzymes. *Nat. Commun.* **2022**, *13* (1), 7458. https://doi.org/10.1038/s41467-022-35228-y.

(14) Shin, J.-S.; Kim, B.-G. Exploring the Active Site of Amine:Pyruvate Aminotransferase on the Basis of the Substrate Structure−Reactivity Relationship: How the Enzyme Controls Substrate Specificity and Stereoselectivity. *J. Org. Chem.* **2002**, *67* (9), 2848–2853. https://doi.org/10.1021/jo016115i.

(15) Shin, J.-S.; Yun, H.; Jang, J.-W.; Park, I.; Kim, B.-G. Purification, Characterization, and Molecular Cloning of a Novel Amine:Pyruvate Transaminase from Vibrio Fluvialis JS17. *Appl. Microbiol. Biotechnol.* **2003**, *61* (5–6), 463–471. https://doi.org/10.1007/s00253-003-1250-6.

(16) Humble, M. S.; Cassimjee, K. E.; Håkansson, M.; Kimbung, Y. R.; Walse, B.; Abedi, V.; Federsel, H.; Berglund, P.; Logan, D. T. Crystal Structures of the *Chromobacterium Violaceum* Ω-transaminase Reveal Major Structural Rearrangements upon Binding of Coenzyme PLP. *FEBS J.* **2012**, *279* (5), 779–792. https://doi.org/10.1111/j.1742-4658.2012.08468.x.

(17) Ruggieri, F.; Campillo-Brocal, J. C.; Chen, S.; Humble, M. S.; Walse, B.; Logan, D. T.; Berglund, P. Insight into the Dimer Dissociation Process of the Chromobacterium Violaceum (S)-Selective Amine Transaminase. *Sci. Rep.* **2019**, *9* (1), 16946. https://doi.org/10.1038/s41598-019-53177-3.

(18) Kollipara, M.; Matzel, P.; Sowa, M.; Brott, S.; Bornscheuer, U.; Höhne, M. Characterization of Proteins from the 3N5M Family Reveals an Operationally Stable Amine Transaminase. *Appl. Microbiol. Biotechnol.* **2022**, *106* (17), 5563–5574. https://doi.org/10.1007/s00253-022-12071-1.

(19) Schneider, G.; Käck, H.; Lindqvist, Y. The Manifold of Vitamin B6 Dependent Enzymes. *Structure*, **2000**, *8* (1), R1–R6. https://doi.org/10.1016/S0969-2126(00)00085-X.

(20) Wang, H.; Xie, Y.; Wang, J.; Fan, H.; Wei, D. S type ω-transaminase ATA-W12 and gene and application thereof. CN106520719A, 2017

(21) Matassa, C.; Romani, A.; Ormerod, D.; Bornscheuer, U. T.; Höhne, M.; Satyawali, Y. Jeffamine® ED-600: A Polyether Amine Donor for Enzymatic Transamination in Organic Solvent/Solvent-free Medium with Membrane-assisted Product Extraction. *J. Chem. Technol. Biotechnol.* **2020**, *95* (3), 604–613. https://doi.org/10.1002/jctb.6241.

(22) Yang, L.; Zhang, Y.; Liu, Y.; Wang, H.; Wei, D. Highly Efficient Synthesis of Pharmaceutically Relevant Chiral 3-N-Sub stituted-Azacyclic Alcohols Using Two Enantiocomplementary Short Chain Dehydrogenases. *Biochem. Eng. J.* **2022**, *178*, 108300. https://doi.org/10.1016/j.bej.2021.108300.

(23) Inoue, K.; Makino, Y.; Itoh, N. Purification and Characterization of a Novel Alcohol Dehydrogenase from Leifsonia Sp. Strain S749: A Promising Biocatalyst for an Asymmetric Hydrogen Transfer Bioreduction. *Appl. Environ. Microbiol.* **2005**, *71* (7), 3633–3641. https://doi.org/10.1128/AEM.71.7.3633-3641.2005.

(24) Inoue, K.; Makino, Y.; Dairi, T.; Itoh, N. Gene Cloning and Expression of *Leifsonia* Alcohol Dehydrogenase (LSADH) Involved in Asymmetric Hydrogen-Transfer Bioreduction to Produce (*R*)-Form Chiral Alcohols. *Biosci. Biotechnol. Bio chem.* **2006**, *70* (2), 418–426. https://doi.org/10.1271/bbb.70.418.

(25) Weckbecker, A.; Hummel, W. Cloning, Expression, and Characterization of an (*R*)-Specific Alcohol Dehydrogenase from Lactobacillus Kefir. Biocatal. Biotransformation **2006**, 24 (5), 380–389. https://doi.org/10.1080/10242420600893827.
(26) Rodríguez, C.; Borzęcka, W.; Sattler, J. H.; Kroutil, W.; Lavandera, I.; Gotor, V. Steric vs. Electronic Effects in the Lacto bacillus Brevis ADH-Catalyzed Bioreduction of Ketones. *Org Biomol Chem* **2014**, *12* (4), 673–681. https://doi.org/10.1039/C3OB42057D.
(27) Hummel, W.; Riebel, B. Isolation and biochemical characterization of a new NADH oxidase from Lactobacillus brevis *Biotechnol. Lett.* **2003**, *25* (1), 51–54. https://doi.org/10.1023/A:1021730131633.
(28) Wilkins, M. R.; Gasteiger, E.; Bairoch, A.; Sanchez, J.-C.; Williams, K. L.; Appel, R. D.; Hochstrasser, D. F. Protein Identi fication and Analysis Tools in the ExPASy Server. In 2-D Proteome Analysis Protocols; Humana Press: New Jersey, 1998; Vol. 112, pp 531–552. https://doi.org/10.1385/1-59259-584-7:531.
(29) Schätzle, S.; Höhne, M.; Redestad, E.; Robins, K.; Bornscheuer, U. T. Rapid and Sensitive Kinetic Assay for Characteri zation of ω-Transaminases. *Anal. Chem.* **2009**, *81* (19), 8244–8248. https://doi.org/10.1021/ac901640q.
(30) Truppo, M. D.; Rozzell, J. D.; Moore, J. C.; Turner, N. J. Rapid Screening and Scale-up of Transaminase Catalysed Reac tions. *Org Biomol Chem* **2009**, *7* (2), 395–398. https://doi.org/10.1039/B817730A.
(31) Sarver, P. J.; Bissonnette, N. B.; MacMillan, D. W. C. Decatungstate-Catalyzed C(Sp3)–H Sulfinylation: Rapid Access to Diverse Organosulfur Functionality.
(32) Schultz, D. M.; Levesque, F.; DiRocco, D. A.; Reibarkh, M.; Ji, Y.; Joyce, L. A.; Dropinski, F. J.; Sheng, H.; Sherry, B.D.; Davies., I.W. Oxyfunctionalization of the Remote C-H Bonds of Aliphatic Amines by Deca-tungstate Photocatalysis. *Angew. Chem. Int. Ed.* **2017**, *56*, 15274 –15278
(33) Marfey, P. Determination ofD-Amino Acids. II. Use of a Bifunctional Reagent, 1,5-Difluoro-2,4-Dinitrobenzene. *Carlsberg Res. Commun.* **1984**, *49* (6), 591–596. https://doi.org/10.1007/BF02908688.
(34) *Handbook of Thin-Layer Chromatography*, 0 ed.; Sherma, J., Fried, B., Eds.; CRC Press, 2003. https://doi.org/10.1201/9780203912430.
(35) Han, Z.-J.; Li, Y.-B.; Gu, B.-H.; Li, Y.-M.; Chen, H. Economical Synthesis of *Tert*- Butyl (*S*)-3-Aminopyrrolidine-1-Carboxylate from L-Aspartic Acid. *Synth. Commun.* **2018**, *48* (18), 2452–2456. https://doi.org/10.1080/00397911.2018.1509093.
